# Carboxyl Dissociation Degree (*α*) and p*K*_a_ of Weak Polyelectrolyte Membranes in Dilute and Concentrated External Salt Solutions for Sustainable Technologies

**DOI:** 10.1021/acs.macromol.5c02531

**Published:** 2026-05-04

**Authors:** Yongha Kim, Michael A. Shaqfeh, Charleen M. Rahman, Riley B. Kracaw, Steven D. Marotta, Nikitha S. Kanumuru, Ania Chandra, Andrew J. Lukaszewski, Lauren Collins, Hee Jeung Oh

**Affiliations:** † Department of Chemical Engineering, 311285 The Pennsylvania State University, University Park, Pennsylvania 16802, United States; ‡ Department of Chemistry, The Pennsylvania State University, University Park, Pennsylvania 16802, United States; § Department of Materials Science and Engineering, The Pennsylvania State University, University Park, Pennsylvania 16802, United States; ∥ Department of Biomedical Engineering, The University of Michigan, Ann Harbor, Michigan 48104, United States; ⊥ Institute of Energy and Environment (IEE), The Pennsylvania State University, University Park, Pennsylvania 16802, United States; # Advanced Manufacturing and Design, The Pennsylvania State University, University Park, Pennsylvania 16802, United States

## Abstract

Understanding the dissociation process in weakly charged polymer membranes is essential to design innovative charged polymer membranes with desirable transport properties for broader applications in sustainability. Previously, we designed a library of weakly charged polymer membranes, i.e., cross-linked acrylic acid (AA)–poly­(ethylene glycol) diacrylate (PEGDA) (AA–PEGDA) random copolymer networks with a wide ion-exchange capacity (IEC = 0–4 mequiv/g) range and limited water swelling. Here, we report the dissociation process of the weakly charged AA–PEGDA series in dilute (DI water) and concentrated (1 M NaCl (aq)) external salt solutions by probing dissociation in both 1) a solution phase (via potentiometric (POT) titration) and 2) a polymer phase (via ATR–FTIR analysis). Both POT titration and ATR–FTIR analysis describe the dissociation process well, showing similar dissociation parameters (degree of ionization (*α*), p*K*_a_). As the external pH increases (pH = 3–12), the degree of ionization (*α*) increases between 0 and 1, following the modified Henderson–Hasselbalch equation. The pre-titration analysis also confirms that our AA–PEGDA series shows a very small dissociation degree (*α* = 0–4%) before adding a strong base (NaOH). To determine the dominant molecular factors affecting the electrostatic interaction (thus, dissociation) in our system, we compare four relevant physical length scales, i.e., (1) the average charged group distance in a polymer (*r_c_*), (2) the average spacing between salt ions in an external solution (*r_ion_*), and the respective (3) Bjerrum length (*l_B_*) and (4) Debye screening length (*r_D_*) in dilute and concentrated salt solutions. In the dilute solution (DI water), the enhanced electrostatic interaction (*r_c_* ≲ *l_B_* ≪ *r_D_* < *r_ion_*) is the dominant factor for suppressing the dissociation and thus moving p*K*_a_ to a higher range. The influence of varying polymer compositions (mIEC and PEGDA cross-linker length, *n*) has a relatively smaller impact on dissociation and p*K*_a_ in dilute conditions. In contrast, in the concentrated salt solution (1 M NaCl (aq)), the screened electrostatic interaction (*r_D_* ≪ *r_c_* ≲ *l_B_* ≈ *r_ion_*) via added external salts is the governing factor for promoting dissociation and thus substantially lowering p*K*_a_. Subsequently, changing polymer compositions (mIEC, *n*) shows greater influences on dissociation and p*K*_a_. If an external salt condition is fixed, varying polymer compositions (mIEC, *n*) in the AA–PEGDA series leads to different degrees of water swelling (*ϕ_w_*) (high *ϕ_w_* favors more dissociation), altering the dissociation trend and p*K*_a_. In our system, varying external salt conditions and water swelling (*ϕ_w_*) ratios in the polymers are the major parameters for controlling dissociation behavior and p*K*_a_. Overall, our AA–PEGDA series follows a similar trend to that of other AA-based polymers in the literature, showing higher p*K*_a_ values in dilute solutions and lower p*K*_a_ range in concentrated salt solutions. To the best of our knowledge, this is the first time that the dissociation process has been systematically studied using a film form with systematically varied polymer compositions and external conditions. Our multiscale analysis of dissociation using the AA–PEGDA platform can help us understand the molecular-level physical picture of dissociation in weakly charged polymers and thus tune the dissociation of this material to achieve desired transport properties.

## Graphical abstract

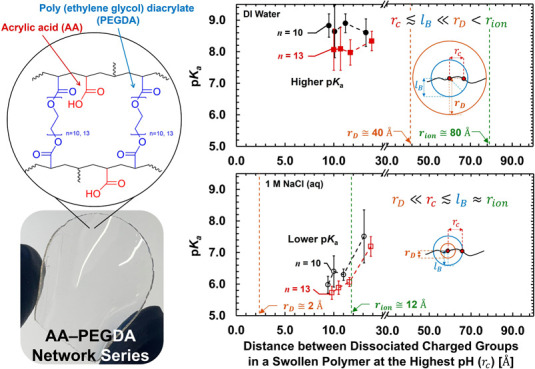

## 1 Introduction

A charged polymer membrane serves as a versatile component in a wide range of applications in energy, environment, and health because of its selective and controlled transport of small molecules across the membrane.^1–6^ Representative applications include energy generation (e.g., batteries and fuel cells), water purification, resource recovery, environmental remediation, and health-related devices,^5–15^ to name a few. To design an innovative charged polymer membrane offering desirable transport properties for a target application, it is essential to understand the effects of the charged group content (type and concentration) on ion and water transport in a polymer. However, it is often difficult to decouple the effects of charged group content on ion transport from the influences of other related variables, such as polymer morphology and high water swelling, originating from the varied charged group content in the polymer.^16–21^ For instance, altering the charged group concentration in a polymer often requires different processing parameters (e.g., solvent, formation methods), simultaneously leading to different morphologies. Also, increasing charged group contents results in high water swelling in the polymer. High water swelling often overshadows any influences arising from tuning the charged group content in highly swollen polymers. These coupled effects make a mechanistic understanding of ion transport in charged polymers challenging.

To overcome these current limitations, we previously designed a library of weakly charged polymer membranes, i.e., cross-linked acrylic acid (AA)–poly­(ethylene glycol) diacrylate (PEGDA) (AA–PEGDA) random copolymer networks with a wide ion-exchange capacity (IEC = 0–4 mequiv/g) range and limited water swelling (*ϕ_w_* = 0.07–0.69).^22^ Weakly acidic acrylic acid (AA) monomer was chosen as a charged block (p*K*_a_ of AA monomer is between 4.24 and 4.60 ^23–26^ ). Poly­(ethylene glycol) diacrylate (PEGDA) cross-linkers with different molecular weights were used to control cross-linking densities in the networks and thus limit high water swelling. Using this model polymer, in one fixed chemical structure, the charged group concentration can be systematically changed (degree of ionization, *α* = 0–1) as the external pH is varied between pH = 3–12. If pH ≪ p*K*_a_, almost all of the dissociable charged groups (COOH in this study) do not dissociate (degree of ionization, *α* = 0), and the polymer behaves like a neutral polymer. If pH = p*K*_a_, one-half of the dissociable charged groups (COOH) dissociates and presents as carboxylate groups (COO^–^ in this study), and thus, *α* is 0.5. If pH ≫ p*K*_a_, all of the dissociable charged groups (COOH) dissociate (*α* = 1), and the polymer is fully ionized. Therefore, in the same polymer chemical structure, we can systematically control the amount of charged group concentration without changing the polymer architecture or morphology while limiting high water swelling.

As the first stepping stone toward building a mechanistic understanding of ion transport, it is important to understand the dissociation process vs pH in our weakly charged AA–PEGDA polymer system. While the dissociation (or neutralization) process of nonpolymeric acids and bases has been extensively investigated, quantifying the dissociation process in ion-exchange resins and polyelectrolyte polymers is still largely underdeveloped.^23,27,28^ Dissociation parameters, such as the degree of ionization (*α*) and the negative logarithm of the apparent acid dissociation constant (p*K*_a_), are strongly dependent on polymer composition, morphology, form factors, and external conditions. Thus, it is necessary to use a systematic platform to understand the dissociation process in polyelectrolyte polymers. However, in the literature, polyelectrolyte polymers, including acrylic acid (AA)-based polymers, were studied using different forms (e.g., polymer solutions, porous beads with different pore sizes and porosities, films, and/or composite structures) with varied cross-linker contents (type and concentration) in different external conditions (salt type and concentration),^24,29–31^ making proper comparison difficult among them. In contrast, our AA–PEGDA series was prepared in thin-film form of uniform thickness with systematically altered polymer composition (i.e., AA monomer content—the amount of dissociable charged groups—and PEGDA cross-linker concentration with different cross-linker lengths (*n =* 10 and 13)). This model system enables a systematic understanding of the dissociation process in weak polyelectrolyte polymers.

In this context, we previously reported the degree of ionization (*α*) and p*K*_a_ in the AA–PEGDA series in dilute solution (i.e., deionized (DI) water) via three rigorous analytical methods, which measure dissociation: 1) in a solution phase (via potentiometric (POT) titration), 2) in a polymer phase (via ATR–FTIR analysis), and 3) through surface hydrophilicity (via contact angle measurement).^32^ All three analytical methods reasonably describe the molecular-level physical picture of the dissociation process in the polymers. In all compositions, as pH increases (pH = 5–12), the degree of ionization (*α*) increases between 0 and 1 in the dilute conditions, following the modified Henderson–Hasselbalch equation. Compared to p*K*_a_ ranges of the AA monomer (p*K*_a_ ∼ 4.24–4.60)^23–25^ as well as linear AA polymers (p*K*_a_ ∼ 5.41–5.86)^23,24^ dissolved in solutions, our cross-linked AA-PEGDA series shows a significantly increased p*K*_a_ value (p*K*_a_ ∼ 7.98–9.00) because of its higher cross-linking density in covalently bonded polymer network and dilute test conditions (further discussed in Section 4.6). Restricted chain conformation due to cross-linking, as well as dilute test conditions, enhances the electrostatic repulsion between charged groups in the polymers, leading to suppressed dissociation and increased p*K*_a_.

While our previous report serves as a control baseline to understand the dissociation of weakly charged polymers in dilute conditions (in DI water) (note that NaOH (a strong base) concentration range during pH titration was between *C_s_* = 0.002 M and 0.005 M and below the saturation limit $\left(C_{s}^{*}\right)$ ^32^), most real-life membrane-based applications are performed in concentrated salt solution conditions.^6,10,33,34^ For instance, in reverse-osmosis (RO)-based desalination, the salt concentration of seawater is approximately 0.6 M (salinity is 3.5%).^35^ Since NaCl is the most abundant salt in nature, a 1 M aqueous NaCl salt solution is widely adopted to report transport properties of polymer membranes in the literature. Extensive transport property data sets of polymer membranes have been accumulated using 1 M NaCl (aq) solution and are available for comparison.^16–21^ Therefore, it is necessary to understand the dissociation process of the AA–PEGDA series in the representative concentrated salt solution, i.e., 1 M NaCl (aq), for systematic studies.

In this paper, we report the dissociation process of weakly charged AA–PEGDA series in a concentrated salt solution condition (1 M NaCl (aq)) through systematically probing dissociation in both 1) a solution phase (via POT titration) and 2) a polymer phase (via ATR–FTIR analysis). The new dissociation data in the concentrated salt solution are then compared with the dissociation behavior in dilute solutions (DI water) from our previous study.^32^ By systematically changing the external pH (pH = 3–12), we record the degree of ionization (*α*) and p*K*_a_ as a function of polymer composition (i.e., maximum ion-exchange capacity (mIEC) and PEGDA cross-linker length (*n*)) and external salt concentrations (i.e., dilute (DI water) and concentrated (1 M NaCl (aq)) solutions). In addition, to understand the dissociation baseline before titration, we record the dissociation degree (*α*) before adding a strong base (NaOH (aq) in this study) vs polymer composition and external salt conditions. Although it is assumed that the dissociation before titration is negligible, pre-titration dissociation data before adding a strong base is underdeveloped in the literature.^23,27–30^ To analyze our dissociation data and obtain p*K*_a_ values, we used the modified Henderson–Hasselbalch equation.

Our thorough interpretation of the dissociation process is performed in three-fold. First, to determine the governing molecular factors of the polymers on dissociation, we investigate the dissociation parameters (*α*) and p*K*_a_ as a function of representative polymer variables, i.e., charged group concentration $\left(C_{C}^{m}\right)$ , cross-linking density (*v_t_*), and equilibrium water swelling (*ϕ_w_*). Second, to determine the dominant molecular factors on the electrostatic interaction (and thus, dissociation) in our system, we compare four relevant physical length scales, i.e., the average charged group distance in a polymer (*r_c_*), the average spacing between salt ions in an external solution (*r_ion_*), and the respective Bjerrum length (*l_B_*) and Debye screening length (*r_D_*) in dilute and concentrated salt solutions. Third, we compare the p*K*_a_ trend in our AA–PEGDA series with other AA-based polymers in the literature to build a comprehensive understanding of dissociation among similar weakly charged polymers.

The pre-titration analysis confirmed that our AA–PEGDA series shows a very small dissociation degree (*α* = 0–4 %) before adding a strong base. While the extent of the dissociation degree is very small, the dissociation trend is consistent with respect to polymer composition and external salt conditions. When titrated, the dissociation process in our AA–PEGDA series follows the modified Henderson–Hasselbalch equation. Both POT titration (probing a solution phase) and ATR–FTIR analysis (detecting a polymer phase) describe the dissociation process well, showing similar ranges of dissociation parameters (*α*, p*K*_a_). The overall dissociation trend was clearly described by comparing relevant molecular-level physical length scales (*r_c_*, *r_ion_*, *l_B_*, and *r_D_*) in our system. Compared to other AA-based polymers in the literature, our AA–PEGDA series shows higher p*K*_a_ values in dilute conditions and lower p*K*_a_ range in concentrated salt conditions, following the general trend of other polymers.

In our system, varying external salt conditions and water swelling (*ϕ_w_*) ratios in the polymers are the major parameters for controlling the dissociation behavior and p*K*_a_. Our multscale analysis of dissociation can help us tune the dissociation behavior of weakly charged polymers to achieve desired transport properties. To the best of our knowledge, this is the first time that the dissociation process has been systematically studied using a film form with systematically varied polymer composition and external conditions. Using this platform, our subsequent paper will further discuss the effect of external salt concentrations on the dissociation process using a fixed chemical structure in order to advance our understanding of dissociation. Together, this system can offer a versatile foundation to design next-generation polymer membranes for sustainable technologies.

## 2 Experiments

### 2.1 Materials

We synthesized a series of cross-linked acrylic acid (AA)–poly (ethylene glycol) diacrylate (PEGDA) (AA–PEGDA) random copolymer networks via UV-induced free radical polymerization, as shown in Figure 1. Acrylic acid (AA, 8.00181, Millipore Sigma, Burlington, MA) was used as a monomer with a weakly charged, dissociable carboxylic acid group (COOH). Poly­(ethylene glycol) diacrylates (PEGDAs, 437441 and 455008, Millipore Sigma, Burlington, MA) with different cross-linker lengths (*n* = 10, *M̅_n_* = 575 g/mol and *n* = 13, *M̅_n_* = 700 g/mol) were used as cross-linkers. Deionized (DI) water (18.2 MΩ·cm, Millipore Direct-Q 5 UV system, Merck, Germany) was added as a solvent, and 2,2-dimethoxy-2-phenylacetophenone (DMPA, 196118, Millipore Sigma, Burlington, MA) was used as a photoinitiator for the polymerization. All polymerization reactants were purchased and used as received. Sodium chloride (NaCl, S9888, Millipore Sigma, Burlington, MA) was used for preparing an aqueous 1 M NaCl solution. Sodium hydroxide (NaOH, S5881, Millipore Sigma, Burlington, MA) was used to prepare aqueous NaOH solutions for titration.

**Figure 1:**
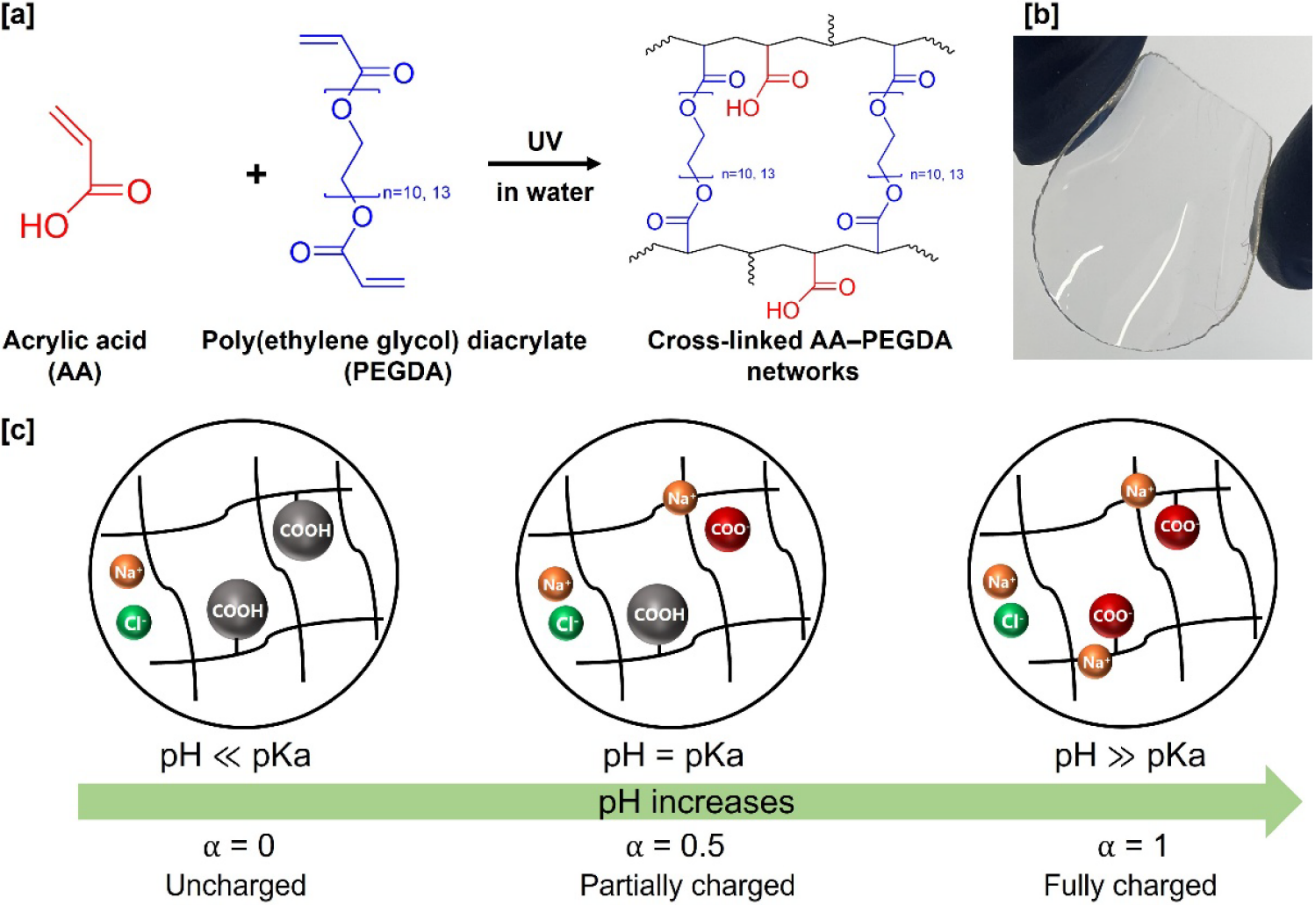
**[a]** Chemical structure of cross-linked acrylic acid (AA)–poly­(ethylene glycol) diacrylate (PEGDA) (AA–PEGDA) random copolymer networks via UV-induced free radical polymerization. **[b]** Picture showing a transparent, uniform-thickness, free-standing thin film of 10–2 AA–PEGDA network. The 10–2 formulation has the maximum ion-exchange capacity (mIEC) = 2 mequiv/g with PEGDA cross-linker length, *n* = 10. **[c]** Schematic describes the dissociation process vs pH in AA–PEGDA series.

### 2.2 Membrane Preparation

Transparent, uniform-thickness thin films of cross-linked AA–PEGDA polymer network series were prepared via UV-induced free-radical polymerization (see Figure 1a-b), as reported previously.^22,32^ The desired amount of AA monomer, PEGDA cross-linker, solvent (DI water), and DMPA photoinitiator (see Table S1) was well-mixed for 30 min to prepare the homogeneous prepolymer mixture solution in a glass jar covered by aluminum foil. After gentle stirring for 30 min, 3 mL of the prepolymer mixture solution was poured onto a leveled quartz plate (CGQ-0620–20, Chemglass Life Sciences, Vineland, NJ). Four spacers with a thickness of 305 μm (19470, Precision Brand, Downers Grove, IL) were located at the edges between two quartz plates to form a uniform-thickness thin film. Then, the prepolymer mixture solution between the two plates was placed under a UV lamp (254 nm) (Spectrolinker XL-1000 UV Cross-linker, Spectronics Corporation, Melville, NY) for 90 s to form a transparent film with a uniform thickness, as shown in Figure 1b. The film was soaked in DI water for 2–3 days, while DI water was changed frequently to remove unreacted reactants from the film. Circular polymer coupons with a diameter of 2.2 cm (7/8 in.) were produced using a punch die (66002, Mayhew Steel Products, Turners Falls, MA) for characterization. The average conversion rate of the AA–PEGDA series is 98.4 ± 1.2 %, as reported previously,^22^ confirming successful polymerization.

Nomenclature of AA–PEGDA network is *n*–mIEC, where *n* denotes the number of ethylene oxide (EO) repeating units in a PEGDA cross-linker (i.e., *n* = 10 or 13), and mIEC is the maximum ion-exchange capacity of the polymer. For instance, 10–2 AA–PEGDA network has an average of 10 EO repeating units between two network junctions (in the PEGDA cross-linker of *M̅_n_* = 575 g/mol), and its mIEC is 2 mequiv/g.

### 2.3 Theoretical Cross-Linking Density

Theoretical cross-linking density (*v_t_*) of a polymer was determined from the concentration of vinyl (C=C) groups in a PEGDA cross-linker in an AA–PEGDA network as^36,37^

vt[Molesofvinylgroups[mol]Volumeof⁢⁢adrypolymer[cm3]]=2·mcM̅nmdρp 1

where *m_c_* is the mass of a PEGDA cross-linker [g], *m_d_* is the mass of a dry polymer sample [g], *M̅_n_* is the number-average molecular weight of the PEGDA cross-linker [g/mol], and *ρ_p_* is the polymer density [g/cm^3^]. The prefactor of 2 accounts for the fact that each PEGDA cross-linker contains two vinyl groups that contribute to cross-linking. In this study, theoretical cross-linking density (*v_t_*) was used to broadly compare the polymer network nature (e.g., tighter or looser network) of AA–PEGDA series as a function of PEGDA cross-linker content. Note that, because of wasted cross-links or self-loops, which are likely present in a network, theoretical cross-linking density (*v_t_*) is higher than effective cross-linking density (*v_e_*), which only counts the concentration of network junctions contributing to the network elasticity.^36,38,39^

### 2.4 Potentiometric (POT) Titration

Potentiometric (POT) titration was conducted to control the amount of dissociated charged carboxylate groups (COO^–^) in AA–PEGDA network films. A circular polymer coupon (diameter: 2.2 cm) was dried at 50 °C for 2 days in a vacuum oven (281A, Thermo Fisher Scientific, Waltham, MA) to remove water from the polymer sample. Dry masses (*m_d_*) were measured using an analytical balance (MS304TS/00, Mettler Toledo, Columbus, OH) until an equilibrium dry mass was reached. Then, the polymer coupon was soaked in 100 mL of a desired solution (DI water or 1 M NaCl (aq) solution). Wet masses (*m_w_*) of the polymer coupon were frequently measured for 2 days to reach equilibrium water swelling. The pH of the external solution equilibrated with the polymer coupon was also measured by using a pH meter (SevenDirect SD50, Mettler Toledo, Columbus, OH). Note that, prior to solution preparation, DI water was equilibrated in air at ambient temperature (21–22 °C) for 24 h to reach the equilibrium amount of dissolved carbon dioxide (CO_2_ (g)) in the DI water. Before the polymer coupon was soaked, the pH value of the solution was measured. The pH values of the DI water and 1 M NaCl (aq) solution were in the range of pH = 6.0 ± 0.2 and pH = 6.1 ± 0.2, respectively. Our measured pH values before immersing the coupon are in a similar range (pH = 5.65) as those reported in the literature.^22,32,40,41^

A varied amount of 0.1 M NaOH (aq) solution (*x*_*NaOH*_ [mequiv/g]) was added to the solution equilibrated with the polymer coupon to reach a desired pH in the external solution (see Table S2). The jar containing the polymer coupon and the solution was placed on an orbital shaker (Labnique, Hunt Valley, MD) for 24 h to complete the titration. Once the titration was completed, the final pH of the solution and the equilibrium wet mass of the polymer coupon were measured. The wet polymer coupon was then dried at 50 °C overnight in a vacuum oven. The polymer density (*ρ_p_*) of the dry polymer coupon was measured before and after the titration. ATR–FTIR analysis was performed on the wet and dry polymer coupons at each step to confirm that no chemical degradation occurred during the pH titration (see Figures S9–S12). Detailed procedures have been reported in our previous papers^22,32^ (see Figures S1–S2).

### 2.5 Polymer Characterization

#### 2.5.1 Polymer Density

Polymer density (*ρ_p_* [g/cm^3^]) was measured using a density measurement kit (ML-DNY-43, Mettler Toledo, Columbus, OH) via Archimedes’ principle as^42,43^

2 $$\rho_{p}=\frac{m_{d}}{m_{d}-m_{l}}\times \rho_{l}$$

where *m_d_* and *m_l_* are the dry masses of a polymer sample in air and an auxiliary liquid, respectively, and *ρ_l_* is the density of the auxiliary liquid. Dry masses were measured after drying the polymer sample at 50 °C in a vacuum oven for 2 days, before and after pH titration (see Section 2.4). In this study, the auxiliary liquid was *n*-heptane (34873, Millipore Sigma, Burlington, MA), since the AA–PEGDA series shows negligible uptake of *n*-heptane in the polymers.^44^

#### 2.5.2 Equilibrium Water Swelling (*ϕ_W_*)

Equilibrium water swelling (*ϕ_w_*) of AA–PEGDA networks was measured by a gravimetric water uptake experiment. A polymer coupon (diameter of 2.2 cm) was dried in a vacuum oven at 50 °C for 2 days. Dry masses (*m_d_*) were frequently measured to obtain equilibrium values. Subsequently, the coupon was soaked in a desired solution (DI water or 1 M NaCl (aq) solution) at ambient temperature (21–22 °C) before and after titration. Wet masses (*m_w_*) of the coupon were measured until equilibrium water swelling was reached. Prior to the measurement, excess water on the coupon surface was gently removed using dust-free tissue papers. Equilibrium water swelling or water uptake [grams of sorbed water/grams of a dry polymer] is expressed as^45–47^

$$w_{u}\;\left[\frac{Mass\;of\;sorbed\;water\;[g]}{Mass\;of\,\;\,a\;dry\;polymer\;[g]}\right]=\frac{m_{w}-m_{d}}{m_{d}}$$ 3

Equilibrium water volume fraction in a swollen polymer [cm^3^/cm^3^, *ϕ_w_*], is the ratio of the volume of sorbed water in a swollen polymer to the volume of a swollen polymer as^45–47^

$$\phi_{W}\;[\frac{Volume\;of\;sorbed\;water\;in\;a\;swollen\;polymer\;[cm^{3}]}{Volume\;of\;a\;swollen\;polymer\;[cm^{3}]}]=\frac{\left(m_{w}-m_{d}\right)/\rho_{w}}{\left(m_{w}-m_{d}\right)/\rho_{w}+m_{d}/\rho_{p}}$$ 4

where *ρ_w_* and *ρ_p_* are the densities of water (1.0 g/cm^3^) and the dry polymer, respectively. We assumed the ideal mixing behavior and the volume additivity of water in a swollen polymer.

#### 2.5.3 ATR–FTIR

Chemical structure and dissociated charged group (COO^–^) contents of AA–PEGDA networks were determined using an attenuated total reflection mode Fourier transform infrared spectroscopy (ATR–FTIR, NICOLET iS50 FTIR, Thermo Fisher Scientific, Waltham, MA). Before titration, a polymer coupon (diameter of 2.2 cm) was soaked in a desired solution (DI water or 1 M NaCl (aq) solution) for 1–2 days, and ATR–FTIR spectra of the polymer coupon were collected. Prior to the measurement, residual water on the coupon surface was gently dabbed using dust-free tissue paper. Then, the polymer coupon was dried at 50 °C overnight in a vacuum oven, and its ATR–FTIR spectra were collected.

During titration, ATR–FTIR was measured at each step to determine the amount of dissociated carboxylate groups (COO^–^) and chemical stability of the polymer coupon. The polymer coupon was titrated by immersing it in a desired solution (DI water or 1 M NaCl (aq) solution) with a varied amount of 0.1 M NaOH (aq) (*x*_*NaOH*_) for 1 day to reach a desired pH. After the titration, ATR–FTIR spectra of the polymer coupon were collected after gently removing residual water from the surface. Then, the polymer coupon was dried at 50 °C overnight in a vacuum oven, and its ATR–FTIR spectra were measured. Background and sample spectra were collected using 64 scans at a resolution of 4 cm^–1^ between 500 and 4000 cm^–1^ for all measurements. The background spectrum was subtracted from the sample spectrum by using software (OMNIC, Thermo Fisher Scientific, Waltham, MA) to obtain the data.

## 3 Theory

### 3.1 Quantification of Dissociated Charged Group Content

Dissociation process in a weakly charged polymer with carboxylic acid groups (COOH) can be expressed as^27^

5 $$R-\mathrm{COOH}⇆R-{\mathrm{COO}}^{-}+H^{+}$$

Degree of ionization (*α*) can be defined as the ratio between the moles of dissociable carboxylic acid groups (COOH) and dissociated carboxylate groups (COO^–^) in a polymer as^27^

$$\alpha =\frac{\left[\mathrm{RCO}O^{-}\right]}{\left[R-\mathrm{COOH}\right]+\left[\mathrm{RCO}O^{-}\right]}\;\left(0\leq \alpha \leq 1\right)$$ 6

In this study, the amount of dissociated charged groups (COO^–^) is quantified using the degree of ionization (*α*) and ion-exchange capacity (IEC, mmol equivalent of H^+^ per grams of a dry polymer [mequiv/g]) of a polymer.^22,27,32^ The maximum ion-exchange capacity (mIEC) is defined as the total amount of *dissociable* charged groups (COOH and COO^–^ in this study) per grams of a dry polymer. The mIEC value of a polymer is solely dependent on polymer composition, i.e., the dissociable charged monomer (AA) content in the polymer. On the other hand, effective ion-exchange capacity (eIEC) represents the amount of *dissociated* charged groups (COO^–^ in this study) in the polymer. The eIEC value of the polymer depends on both polymer composition (i.e., the dissociable charged monomer (AA) content in the polymer) and the external pH. Thus, mIEC and eIEC of the polymer are correlated with the degree of ionization (*α*) as

eIEC=mIEC·α(0≤α≤1) 7

In addition, the amount of dissociated charged groups (COO^–^) in a polymer can be expressed as the charged group concentration in a swollen polymer $(C_{c}^{m}$ [mol/L], where the subscript *c* represents charged groups and the superscript *m* denotes a membrane phase) as^16,18,48^

$$C_{c}^{m}=\frac{Moles\;of\;dissociated\;charged\;grou\mathrm{ps}\;\mathrm{in}\;a\;swollen\;polymer\;[\mathrm{mol}]}{Volume\;of\;sorbed\;water\;in\;a\;swollen\;polymer\;[L]}=\frac{eIEC\cdot \rho_{w}}{w_{u}}$$ 8

where *ρ_w_* is the water density and *w_u_* is the water uptake of the polymer.

### 3.2 Degree of Ionization (*α*) Before Titration

Before adding a strong base, i.e., NaOH for titration, the degree of ionization (*α*) of a polymer coupon was determined by measuring pH changes in an external solution (DI water or 1 M NaCl (aq) solution) equilibrated with the polymer coupon. The detailed procedures have been reported in our previous papers.^22,32^ If a fraction of dissociable carboxylic acid groups (COOH) in the polymer is dissociated and presented as carboxylate groups (COO^–^), H_3_O^+^ concentration in the external solution increases accordingly. Thus, the H_3_O^+^ concentration in the external solution can be converted to the degree of ionization (*α*) before titration as^22,32^

$$\alpha =\frac{Moles\;of\;released{\;H}_{3}O^{+}\;from\;a\;polymer\;coupon\;[\mathrm{mol}]}{Moles\;of\;total\;dissociable\;charged\;groups\;(COOH)\;in\;a\;polymer\;coupon\;[\mathrm{mol}]}=\frac{V_{s}\cdot \left(10^{-pH\;of\;a\;solution\;after\;soaking\;a\;coupon}-10^{-pH\;of\;a\;solution\;before\;soaking\;a\;coupon}\right)}{Moles\;of\;total\;dissociable\;charged\;groups\;(COOH)\;in\;a\;polymer\;coupon\;[\mathrm{mol}]}$$ 9

where *V_s_* is the volume of an external solution (0.1 L).

### 3.3 Degree of Ionization (α_pH_) via POT Titration

Degree of ionization (*α_pH_*) of a polymer was determined during POT titration (Subscript *_pH_* notes that the degree of ionization is estimated via potentiometric pH titration). When a strongly basic NaOH (aq) (*x*_*NaOH*_ [mequiv/g]) is added, the anion (OH^–^) from the NaOH can react with released H^+^ from dissociated charged groups (COO^–^) to form water (H_2_O). As the amount of added NaOH (aq) increases, the amount of dissociated charged groups (COO^–^) correspondingly increases. Thus, the degree of ionization (*α_pH_*) is assumed to be *x*_*NaOH*_/*mIEC*. When the amount of added NaOH (aq) equals the total amount of dissociable charged groups (i.e., mIEC [mequiv/g]) in the polymer, the degree of ionization (*α_pH_*) reaches 1. Thus, the degree of ionization (*α*) can be estimated from the amount of added NaOH (aq) as^22,32^

10 $$\alpha_{pH}\approx \{\begin{array}{l} x_{NaOH}/mIEC\;if\;x_{NaOH}<mIEC\\ 1,if\;x_{NaOH}\geq mIEC \end{array}$$

### 3.4 Degree of Ionization (α_IR_) via ATR–FTIR Analysis

Degree of ionization (*α_IR_*) of a polymer was also estimated using ATR–FTIR analysis, following the methods reported previously.^23,32,49–51^ (Subscript *_IR_* indicates that the degree of ionization was obtained via IR analysis.) Using the Beer–Lambert law, degree of ionization, *α_IR_* was determined as

11 $$\alpha_{IR}=\frac{A_{COO^{-}}}{A_{COO^{-}}+A_{COOH}\cdot F}$$

where *A_COO_*– is the area under the curve at 1575 cm^–1^ (peak associated with dissociated COO^–^ groups), *A*_COOH_ is the deconvoluted area under the curve at 1700 cm^–1^ from AA monomer’s COOH groups, and *F* is the ratio of *A_COO_*– at the highest pH (pH = 11–12, where all COOH groups are dissociated and presented as COO^–^ groups) to *A*_COOH_ at the lowest pH (pH = 3–6, where all COOH groups are not dissociated).

### 3.5 p*K*_a_ via the Modified Henderson–Hasselbalch Equation

The negative logarithm of apparent acid dissociation constant in a polymer, p*K*_a_ is defined as^27^

12 $$pK_{a}=-\log\frac{\left[H^{+}\right]\left[R-COO^{-}\right]}{\left[R-COOH\right]}$$

pH is the negative logarithm of H_3_O^+^ concentration ([H_3_O^+^]) in a solution as^27^

13 $$pH=-\log\;[H_{3}O^{+}]$$

The Henderson–Hasselbalch equation relates the degree of ionization (*α*) with p*K*_a_ and pH in a system as^27^

14 $$pH=pK_{a}+\log\left(\alpha /\left(1-\alpha \right)\right)$$

The modified Henderson–Hasselbalch equation is used to best fit the data as^28^

15 $$pH=pK_{a}+B\cdot \log\left(\alpha /\left(1-\alpha \right)\right)$$

where *B* is the fitting parameter. The degrees of ionization, *α_pH_* and *α_IR_* via POT titration and ATR–FTIR analysis, were used to obtain p*K*_a_ and *B* values.

### 3.6 Molecular-Level Physical Length Scales

To describe the molecular picture of the dissociation process in AA–PEGDA series, we compared four relevant molecular-level physical length scales in our system, i.e., (1) the average distance between dissociated charged groups in a swollen polymer (*r_c_*, subscript *c* notes charged groups), (2) the average distance between salt ions in an external solution (*r_ion_*), and the respective (3) Bjerrum length (*l_B_*) and (4) Debye screening length (*r_D_*).

The average distance between dissociated charged groups (COO^–^) in a swollen polymer (*r_c_*) is determined using effective ion-exchange capacity (eIEC) and equilibrium water swelling (*w_u_*, *ϕ_w_*). We assume the uniform volumetric distribution of charged groups in sorbed water in a swollen polymer as

16 $$r_{c}\;[Å]={\left(\frac{w_{u}\cdot 1000}{\rho_{w}\cdot eIEC\cdot \phi_{w}\cdot N_{A}}\right)}^{1/3}\cdot 10^{8}$$

where *eIEC* is the amount of dissociated charged groups in the dry polymer (see eqs 7 and 8 in Section 3.1), *w_u_* is the water uptake of the polymer, *ρ_w_* is the water density (1 g/cm^3^), *ϕ_w_* is the water volume fraction of the polymer, and *N_A_* is the Avogadro number (6.022 × 10^23^). A charged group (in the polymer) and a salt ion (in an external solution) in a system are treated as a point charge in this simple first approximation.

The average distance between dissociated charged groups in a dry polymer (*r*_*c*,*dry*_) is similarly estimated by assuming the uniform volumetric distribution of charged groups in the polymer as

17 $$r_{c,dry}\;[Å]={\left(\frac{1000}{\rho_{p}\cdot eIEC\cdot N_{A}}\right)}^{1/3}\cdot 10^{8}$$

where *ρ_p_* is the polymer density [g/cm^3^].

Salts used in this study are NaCl and NaOH. The concentration of NaCl (aq) solution (*C_NaCl_*) is 1 M. The added amount of NaOH (aq) (*x_NaOH_* [mequiv/g]) varies from 0 to mIEC [mequiv/g] of a polymer coupon (see Table S2). NaOH (aq) concentration (*C_NaOH_*) is calculated as

$$C_{NaOH}\;\left[M\right]=\frac{m_{d}}{x_{NaOH}\cdot 1000\cdot V_{s}}$$ 18

where *m_d_* is the dry mass of the polymer coupon [g] and *V_s_* is the volume of an external solution (0.1 L). In DI water, the total salt concentration (*C_S_*) during titration is the NaOH (aq) concentration (*C*_S_ = *C_NaOH_*). In 1 M NaCl (aq) solution, the total salt concentration (*C_S_*) in the external solution is the sum of NaCl (aq) and NaOH (aq) concentrations (*C_S_* = *C_NaCl_* + *C*_NaOH_). Using this information, the average distance between salt ions in the external solution (*r_ion_*) is calculated by assuming the uniform volumetric distribution of salt ions in the external solution as

$$r_{ion}\;\left[Å\right]={\left(\frac{1000}{C_{s}\cdot N_{A}}\right)}^{1/3}\cdot 10^{8}$$ 19

Bjerrum length (*l_B_*) represents the distance at which the electrostatic interaction energy between two-point charges (e.g., the charged group in a polymer and the mobile ion in an external solution in our system) equals the thermal energy (*kT*), i.e., the natural energy scale.^52–56^ If the distance (*r*) between the charged group (in a polymer) and the mobile ion (in an external solution) is shorter than the Bjerrum length (*r* < *l_B_*), the electrostatic interaction energy is larger than the thermal energy, and thus, the ion is strongly associated with the charged group (suppressing dissociation). If the distance (*r*) between the charged group and the mobile ion is longer than the Bjerrum length (*r* > *l_B_*), the electrostatic interaction energy is smaller than the thermal energy, and thus, the ion can freely move (escape) from the charged group (promoting dissociation) like noninteracting molecules. Thus, the Bjerrum length (*l_B_*) indicates the strength of electrostatic interaction between the charged group in the polymer and the surrounding mobile ions in the solution and is expressed as

$$l_{B}\;\left[Å\right]=\frac{e^{2}}{4\pi \varepsilon_{p}\varepsilon_{0}k_{B}T}$$ 20

where *e* is the electrostatic charge (1.60218 × 10^–19^ C), *ε_p_* is the dielectric constant of the polymer, *ε*_0_ is the vacuum permittivity (8.8542 × 10^–12^ F/m), *k*_B_ is the Boltzmann constant (1.38065 × 10^–23^ J/K), and *T* is an absolute temperature [K]. In AA–PEGDA series, the *ε_p_* of a swollen polymer is estimated by assuming the volume additivity of the polymer and sorbed water in the polymer as^18,52,53^

21 $$\varepsilon_{p,swollen\;polymer}=\left(1-\phi_{w}\right)\cdot \varepsilon_{p,dry\;polymer}+\phi_{w}\cdot \varepsilon_{w}$$

where *ϕ_w_* is the water volume fraction of the polymer, *ε_w_* is the dielectric constant of water (78.2), and *ε*_*p*,*dry* *polymer*_ is the dielectric constant of the dry polymer (∼12).^18,38,57^ *ε_p,dry polymer_* value was adapted from similar AA- and PEGDA-based polymers in the literature.^18,52,53^

Debye screening length (*r_D_*) illustrates the distance at which the electrostatic potential energy of the charged group in a polymer to randomly fluctuating mobile ions in an external solution equals the thermal energy (*kT*).^53,54,58,59^ If the distance (*r*) between the charged group (in a polymer) and a mobile ion (in an external solution) is closer than the Debye screening length (*r* < *r_D_*), the electrostatic potential energy required for the random fluctuation of mobile ions is smaller than the thermal energy. Consequently, mobile ions can randomly fluctuate, and more oppositely charged ions are present closer to the charged group in the polymer. In this range (*r* < *r_D_*), the electrostatic interaction energy exerted by the charged group is dominant over the thermal energy. The electrostatic interaction energy regulates and influences the movement and arrangement of nearby mobile ions, overcoming the random thermal motion, and electroneutrality is not preserved below the Debye screening length (*r_D_*).

If the distance (*r*) between the charged group and a mobile ion is longer than the Debye screening length (*r* > *r_D_*), the electrostatic potential energy required for the ions’ random fluctuation is larger than the thermal energy, and thus, the charge separation of mobile ions is not permitted. In this range, the electrostatic interaction energy exerted by the charged group is significantly weakened and screened by the redistribution of mobile ions in the solution, and electroneutrality is preserved beyond the Debye screening length (*r_D_*). Therefore, the Debye screening length (*r_D_*) represents the distance where the electrostatic interaction by the charged group is effectively screened by the mobile ions in the solution as

22 $$r_{D}\left[Å\right]=\sqrt{\frac{\varepsilon_{p}\varepsilon_{0}k_{B}T}{N_{A}e^{2}\overset{n}{\underset{i=1}{\sum }}c_{i}z_{i}^{2}}}$$

where $\overset{n}{\underset{i=1}{\sum }}c_{i}z_{i}^{2}$ is expressed as

23 $$\overset{n}{\underset{i=1}{\sum }}c_{i}z_{i}^{2}=2\cdot I$$

where

$$I=\frac{1}{2}\left[C_{Na^{+}}\times {\left(+1\right)}^{2}+C_{Cl^{-}}\times {\left(-1\right)}^{2}+C_{OH^{-}}\times {\left(-1\right)}^{2}\right]=C_{S}\;\left[M\right]$$

## 4 Results and Discussion

### 4.1 Degree of Ionization (*α*) Before Titration

To understand the complete picture of the dissociation process in the weakly charged AA–PEGDA network series, it was necessary to quantify the amount of dissociated charged groups (COO^–^) in the polymer before adding a strong base (i.e., NaOH in this study). The weakly acidic, carboxylic acid group (COOH) in the acrylic acid (AA) monomer slightly dissociates (ionizes) in water and 1 M NaCl (aq) solution as

24 $$R-\mathrm{COOH}+H_{2}O⇄R-{\mathrm{COO}}^{-}+H_{3}O^{+}$$

to form carboxylate anion (R–COO^–^) and hydronium cation (H_3_O^+^), and thus lowers the pH of the solutions. The p*K*_a_ of the AA monomer is 4.24∼4.60 in dilute condition.^23–26,29^

Prior to soaking an AA–PEGDA polymer coupon in two representative regimes of external salt solutions in this study, i.e., dilute (DI water) and concentrated (1 M NaCl (aq)) salt solutions, the solution pH values were close but moderately lower than pH = 7. In DI water, the pH was in the range of pH = 6.0 ± 0.2, whereas in the 1 M NaCl (aq) solution, the pH was around 6.1 ± 0.2. This is due to an equilibrium amount of dissolved carbon dioxide (CO_2_) in water (see Section 2.4). CO_2_ (g) of air dissolves in water to form carbonic acid (CO_2_(g) + H_2_O ⇄ H_2_CO_3_(aq)). The formed carbonic acid (H_2_CO_3_(aq)) can further partially dissociate into a bicarbonate anion (HCO_3_^–^) and a hydronium cation (H_3_O^+^) (H_2_CO_3_(aq) + H_2_O ⇄ HCO_3_^–^ + H_3_O^+^), lowering the pH of the water. As a result, water in equilibrium with CO_2_ (g) of atmospheric air shows a slightly acidic pH of 5.65.^22,32,40,41^ Our measured pH values (pH ∼ 6) in both DI water and 1 M NaCl (aq) solution are in a similar range (pH = 5.65) as reported in the literature. To minimize the solution pH changes originating from the dissolved CO_2_ (g) in water during our experiments, DI water used for pH titration and salt solution preparation in this study was equilibrated with air at ambient temperature for 24 h to reach an equilibrium amount of dissolved CO_2_ (g) in water, as adapted from previous reports.^60^

Note that the presence of neutral salt, i.e., NaCl in water (as 1 M NaCl (aq) solution in this study), does not change the acidity or basicity of the solution and thus does not affect the solution pH. NaCl is formed from the neutralization between a strong acid (hydrochloric acid, HCl) and a strong base (NaOH) (HCl + NaOH → NaCl + H_2_O). Thus, in water, the fully dissociated Na^+^ cation and Cl^–^ anion from the neutral NaCl salt cannot react with water (H_2_O) to generate a base (OH^–^) or an acid (H_3_O^+^). As a result, the addition of NaCl salt to water does not change the concentrations of OH^–^ and H_3_O^+^ in the solution. Their concentrations are the same as those in pure (DI) water ([OH^–^] = [H_3_O^+^] = 10^–7^ M), showing the same pH range as that of pure water. Consistently, our measured pH values in both DI water and 1 M NaCl (aq) solution are in a similar range (pH ∼ 6), as expected.

While the addition of NaCl salt to water (as 1 M NaCl (aq) solution in this study) does not alter the solution pH, the added dissociated ions (Na^+^ and Cl^–^) from the NaCl salt increase the overall ionic strength of the solution. External salt effects from the increased ionic strength can alter the dissociation behavior (*α*) and p*K*_a_ of weakly charged polymers, such as in AA–PEGDA series. Detailed discussion of the effects of external salt conditions on dissociation is provided in Section 4.5.

For the pre-titration study, an AA–PEGDA network film was immersed to reach equilibrium without adding a strong base in the dilute (DI water) and concentrated (1 M NaCl (aq)) solutions. If a small fraction of dissociable charged groups (COOH) in the weakly acidic AA–PEGDA polymer is dissociated, the released H_3_O^+^ from the polymer decreases the pH in the external solution correspondingly. The larger the pH change in the external solution, the more dissociated charged (COO^–^) groups are present in the polymer. By measuring the pH change over time (see Figure 2a), pre-titration degree of ionization (*α*) of the polymer can be determined using eq 9, as shown in Figure 2c.

**Figure 2:**
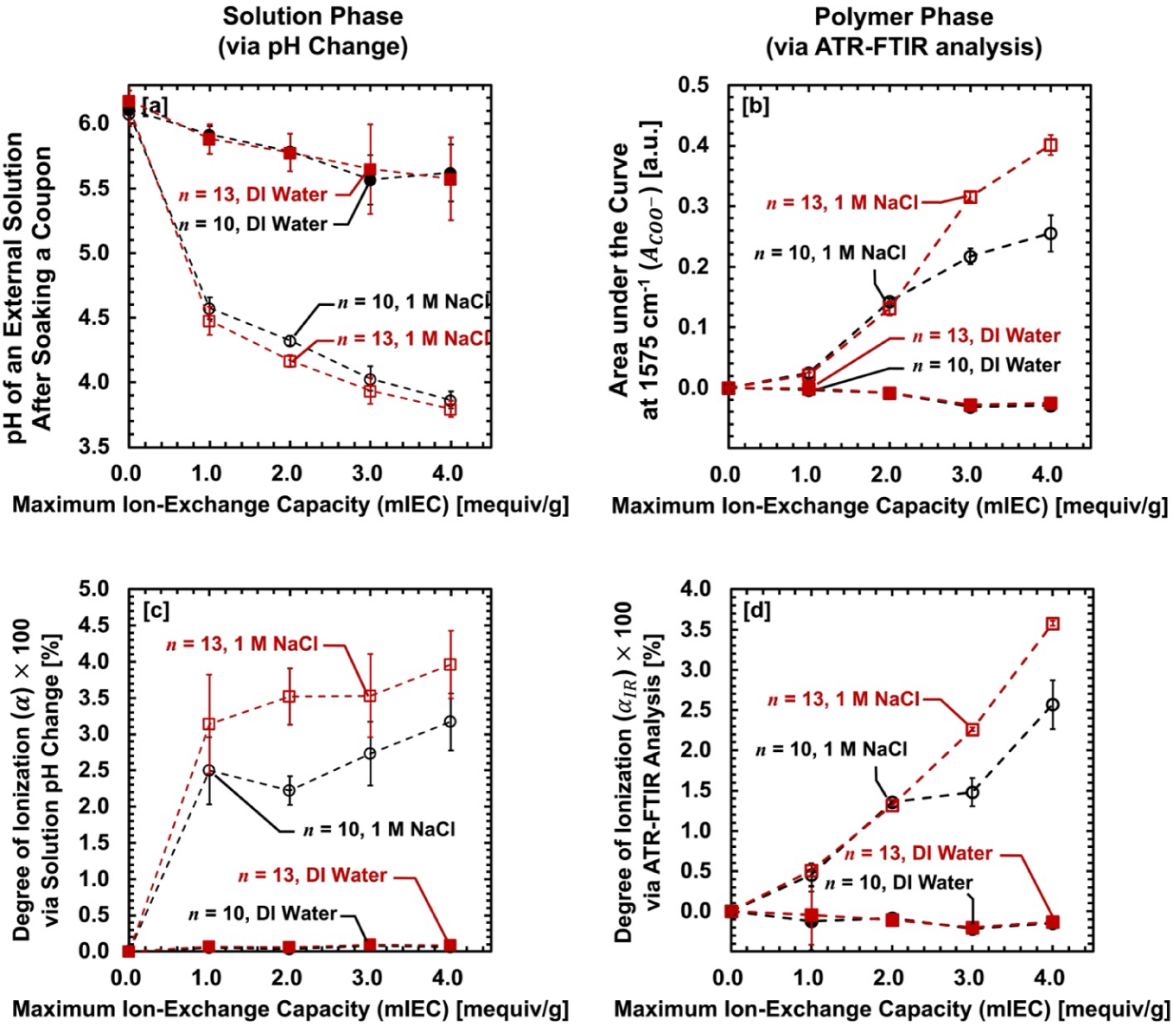
**[a]** pH of an external solution after soaking AA–PEGDA film in DI water or 1 M NaCl (aq) solution for 2 days before titration. No strong base (NaOH) was added. **[b]** Areas under the curve at 1575 cm^–1^ (*A_COO_*–, from dissociated COO^–^ groups) as a function of the maximum ion-exchange capacity (mIEC) [mequiv/g] of dry AA–PEGDA series before titration. **[c]** Pre-titration degree of ionization (*α*) × 100 [%] via solution pH change. **[d]** Pre-titration, degree of ionization (*α_IR_*) × 100 [%] via ATR–FTIR analysis. Dashed lines are used to guide the eyes. Error bars are included. Error bars represent the standard deviation of at least three independent replicate samples.

Figure 2a shows the effects of polymer composition (mIEC = 0∼4 mequiv/g, PEGDA cross-linker length, *n* = 10 and 13) and external salt concentration (dilute vs concentrated solutions) on the pre-titration pH change in the external solution after 2 days of film soaking (also see Figure S3). As a control, cross-linked PEGDA network films (10–0 and 13–0) without any dissociable charged groups (i.e., mIEC = 0 mequiv/g) were shown for comparison. Without any dissociable charged groups in the polymers, pH values in the solution contacting the PEGDA films did not change over time and remained almost constant (pH ∼ 6). This is expected for non-charged, neutral polymers (thus, degree of ionization, *α* = 0).

In contrast, in AA–PEGDA series, the extent of pH changes over time shows a distinct trend as a function of polymer composition (mIEC and *n*) and external salt conditions. First, as mIEC increases from mIEC = 0 mequiv/g to mIEC = 4 mequiv/g (other conditions remain the same in a fixed PEGDA cross-linker length (*n*) and a fixed external salt solution), the extent of pH change is greater because the total amount of dissociable COOH groups increases in the polymers. The increased amount of dissociable COOH groups leads to higher H_3_O^+^ concentration after partial dissociation, resulting in a lower pH in the solution.

Second, as PEGDA cross-linker length (*n*) increases (other conditions are the same), the pH change is also moderately greater, although the difference is relatively smaller in the dilute solution (DI water) and more obvious in the concentrated solution (1 M NaCl (aq)). In general, a looser network with a longer PEGDA cross-linker leads to a higher water swelling in the network (see Figure S21). A water-rich environment favors more dissociation by offering a high dielectric medium for dissociated charged groups. The increased amount of dissociated COO^–^ groups in the looser network leads to a lower pH in the external solution. In the dilute solution (DI water), this trend is likely to be similar, but the differences between *n* = 10 and *n* = 13 series are within the uncertainty of a measurement. This is due to the suppressed dissociation via enhanced electrostatic repulsion between charged groups in dilute solutions, since the electrostatic screening effect of added external salts (NaCl) is excluded. Detailed discussion is provided in Section 4.5.

Third, in a fixed chemical composition (mIEC and *n* are fixed), a polymer film immersed in the concentrated salt solution (1 M NaCl (aq)) shows a greater pH decrease due to the enhanced electrostatic screening effect by the added external salts. As the external salt concentration increases, the electrostatic repulsion between dissociated charged groups decreases via the increased electrostatic screening effect, providing a favorable environment for dissociation. More dissociation in the concentrated salt solution leads to a larger pH decrease (details in Section 4.5).

Figure 2c summarizes the pre-titration degree of ionization (*α*) calculated from the solution pH changes in Figure 2a. Overall, as mIEC increases, PEGDA cross-linker length (*n*) increases, and external salt concentration increases, the degree of ionization (*α*) slightly increases, as expected. However, the extent of the dissociation degree (*α*) without adding a strong base is very small (up to 3–4 %) even in the concentrated salt solution. In the dilute solution (DI water), the pre-titration dissociation degree (*α*) is almost negligible (*α* = 0.05 ± 0.02 %) regardless of the polymer composition.^22^

To further support the pre-titration dissociation data of AA–PEGDA series, we also probed the dissociation process in a polymer phase via ATR–FTIR analysis, as shown in Figure 2b and d (also see Figures S4–S7). While the dissociation results in Figure 2a and c are derived from detecting the pH changes in the solution phase (contacting the polymer film in equilibrium), ATR–FTIR analysis detects the concentration changes of dissociable COOH and dissociated COO^–^ groups in the polymer phase. Combining two methods (probing both solution and polymer phases together) enables a more thorough and rigorous probing of the dissociation process, as discussed in our previous report. Detailed analysis methods have been reported previously (also see Section S1).^23,32,51^

Consistent with the pH changes in the solution phase (in Figure 2a), as mIEC increases (other conditions remain the same) and in the concentrated salt solution (1 M NaCl (aq)), the absorbance intensities (and the areas under the curves) of dissociated COO^–^ groups (at 1575 cm^–1^ and 1400 cm^–1^) increase, as shown in Figure 2b, indicating the increased dissociation degree (*α_IR_*), as expected (also see Figures S6–S7). The absorbance differences between *n* = 10 and *n* = 13 series are also relatively smaller in the dilute (DI water) solution, as expected.

Figure 2d summarizes the pre-titration degree of ionization (*α_IR_*) via ATR–FTIR analysis (the subscript *IR* notes that the degree of ionization was determined by IR analysis). Consistent with the *α* data in the solution phase (see Figure 2c), *α_IR_* results in the polymer phase show similar trends and a similar range of dissociation degrees (0–4 %). As mIEC increases, PEGDA cross-linker length (*n*) increases, and in the concentrated salt solution, the *α_IR_* slightly increases as expected. Minor differences in *α* values via the two methods (i.e., the solution pH change and ATR–FTIR analysis) are likely due to differences in measurement methods, as reported previously.^22,32^ Figure S8 combines all *α* results from both methods.

The pre-titration dissociation analysis confirms that our weakly acidic AA–PEGDA series shows a very small dissociation degree (*α* = 0–4 %). While the extent of dissociation degree (*α*) is very small, the dissociation trend is consistent with respect to polymer composition (mIEC and cross-linker length, *n*) and external salt conditions. The pre-titration dissociation results support the assumption that the major dissociation occurs as a strong base (NaOH) is added during titration.^23,27–30^ Our pre-titration results also serve as a baseline for our further analysis.

### 4.2 pH Titration Curves

We recorded the dissociation process in weakly acidic AA–PEGDA network series as a strong base (NaOH) was added in dilute (DI water) and concentrated (1 M NaCl (aq)) salt solutions via potentiometric (POT) titration. Figure 3 shows the pH titration curves of AA–PEGDA series as a function of the added amount of NaOH (aq) (*x_NaOH_* [mequiv/g]).

**Figure 3:**
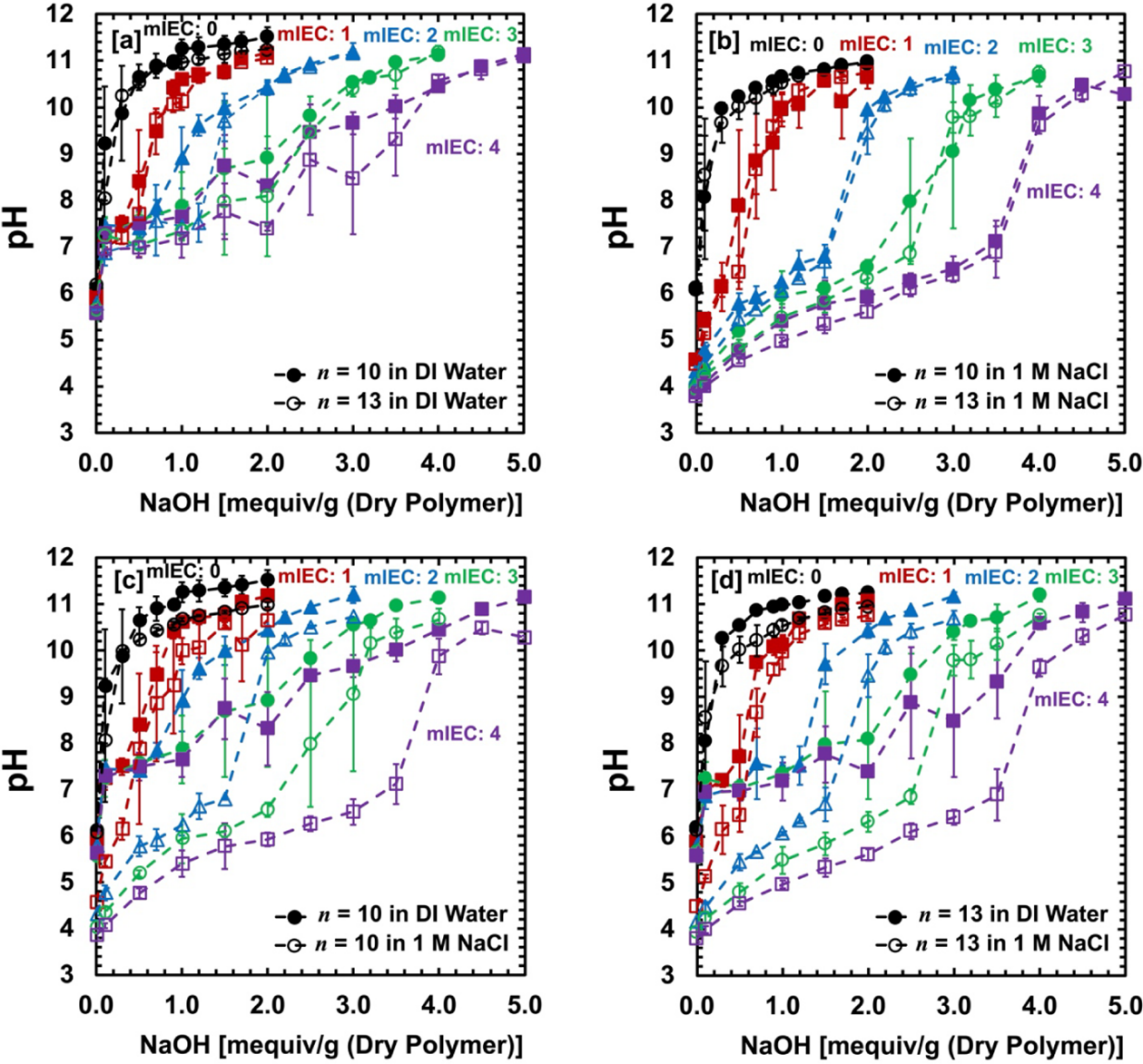
pH vs added NaOH amount [mequiv/g] in PEGDA (mIEC = 0 mequiv/g) and AA–PEGDA series (mIEC = 1–4 mequiv/g). *n* = 10 and *n* = 13 series in **[a]** DI water and **[b]** 1 M NaCl (aq) solution. **[c]** *n* = 10 series in DI water and 1 M NaCl (aq) solution. **[d]** *n* = 13 series in DI water and 1 M NaCl (aq) solution. Dashed lines are used to guide the eyes. Error bars are included. Error bars represent the standard deviation of at least three independent replicate samples.

In a strongly acidic polymer, all strong acid groups dissociate (HA + H_2_O ⇌ H_3_O^+^ + A^–^) in both dilute and concentrated salt (NaCl) solutions.^27^ As a strong base (e.g., NaOH) is added to titrate the strongly acidic polymer, the solution pH remains almost constant until an equivalence point is reached. An equivalence point is where the added amount of OH^–^ equals the amount of H_3_O^+^ in the polymer ([OH^–^] = [H_3_O^+^]). After reaching the equivalence point, the excess amount of OH^–^ ([OH^–^]) steeply increases the pH in the solution. This results in rapid and abrupt increase in pH as the titration approaches its completion point.

In contrast, weakly acidic polymers such as AA–PEGDA series in this study exhibit different trends in pH titration, since weakly acidic groups partially dissociate (HCOOH + H_2_O ⇄ HCOO^–^ + H_3_O^+^).^61^ The acid dissociation constant (*K*_*a*_) is defined as^27^

25 $$K_{a}=\frac{\left[H_{3}O^{+}\right]\cdot \left[A^{-}\right]}{\left[\mathrm{HA}\right]}$$

and can be expressed with pH (= −log­[H_3_O^+^]) as

26 $$\left[H_{3}O^{+}\right]=\frac{K_{a}\cdot \left[\mathrm{HA}\right]}{\left[A^{-}\right]}$$

As a strong base (NaOH) is added, a salt (NaA) is formed (HA + NaOH ⇌ NaA + H_2_O), and thus, the amount of dissociated A^–^ ([A^–^]) decreases accordingly. To maintain stoichiometry, a significant fraction of HA dissociates to present A^–^ (decreased [HA] and increased [A^–^]). This leads to gradually increasing pH (decreased [H_3_O^+^]). At the halfway point, where one-half of the initial amount of HA dissociates, the amount of HA matches the amount of dissociated A^–^ ([HA]_1/2_ = [A^–^]_1/2_), and the degree of ionization, *α* is 0.5. The negative logarithm of the apparent acid dissociation constant in a polymer, p*K*_a_ can be determined as the negative logarithm of the hydronium concentration ([H_3_O^+^]) at the halfway point (pH_1/2_) as

27 $$pK_{a}=pH_{1/2}$$

Before adding the strong base (NaOH), the amount of dissociated A^–^ in the polymer is assumed to be almost negligible.^28^ This is the case in the AA–PEGDA series, as discussed in the previous section (see Section 4.1). The pre-titration dissociation degree (*α*) is negligible (*α* = 0.05 ± 0.02 %) in the dilute solution (DI water) and very small (*α* = 0–4 %) in the concentrated solution (1 M NaCl (aq)). After the halfway point is passed, as the strong base is gradually added, the remaining HA progressively dissociates until it approaches the completion of neutralization (the degree of ionization, *α* = 1), and pH increases in the solution. As the titration is completed, the excess amount of OH^–^ ([OH^–^]) regulates the solution pH, leading to a steeper pH increase.

Our weakly acidic AA–PEGDA series shows the similar pH titration trend, as shown in Figure 3. For comparison, pH titration curves of cross-linked PEGDA networks (10–0 and 13–0) without any dissociable charged groups (i.e., no AA monomers, mIEC = 0 mequiv/g) are shown as controls. Without any dissociable COOH groups, as the strong base (NaOH) is added, PEGDA films show steep increase in pH in the solution in an early stage in both dilute (DI water) and concentrated (1 M NaCl (aq)) solutions. This is expected because all added base (NaOH) contributes to the solution pH without titrating the PEGDA polymers.

On the other hand, in AA–PEGDA series with dissociable COOH groups (mIEC = 1–4 mequiv/g), as the strong base (NaOH) is added, the amount of dissociated COO^–^ groups increases in the polymer, and thus, pH in the solution gradually increases, respectively, as expected. As the added amount of NaOH (*x_NaOH_* [mequiv/g]) equals the total amount of dissociable charged (COOH) groups (mIEC [mequiv/g]) in the polymer, titration (neutralization) reaches the completion point. Near the completion point of titration, the degree of ionization, *α*, approaches 1, and thus, pH steeply increases.^27^

In AA–PEGDA series, the pH titration curves show consistent trends as a function of polymer composition (mIEC) and PEGDA cross-linker length (*n*), and external salt conditions (DI water vs 1 M NaCl (aq)). First, as mIEC (the total dissociable COOH groups in a polymer) increases, the pH titration curve shifts to the right when all other conditions remain the same (with a fixed PEGDA cross-linker length (*n*) and a fixed external salt solution) (see Figure 3a-b). As the total amount of dissociable charged groups (mIEC) increases in a polymer, the total amount of strong base (NaOH) needed for fully neutralizing the polymer correspondingly increases, shifting the titration curve to the right.

Second, in a fixed mIEC and a fixed external salt solution, as PEGDA cross-linker length (*n*) decreases, pH titration curves shift to higher pH ranges. In DI water, the trend is more obvious in the fixed mIEC = 2–4 mequiv/g with increasing mIEC, whereas the titration curves of *n* = 10 and *n* = 13 series in the fixed mIEC = 1 mequiv/g are similar to each other (see Figure 3a). In 1 M NaCl (aq) solution, the upshifting trend with decreasing PEGDA cross-linker length (*n*) is also found in all compositions (mIEC = 1–4 mequiv/g), but the extent of upshifting pH range is smaller (see Figure 3b). This upshifting trend originates from the suppressed dissociation in a tighter cross-linked network. A shorter PEGDA cross-linker forms a tighter cross-linked polymer network with a higher cross-linking density. As the distance between dissociable COOH groups becomes closer in the tighter network, the dissociation is suppressed because of the increased electrostatic interactions.^62–65^ This leads to the increased p*K*_a_ in the tighter network, and the details will be discussed in the following section.

Third, in the fixed composition (mIEC and *n* are fixed), as titration is performed in the concentrated salt solution (1 M NaCl (aq)), pH titration curves shift to lower pH ranges because of the enhanced electrostatic screening effect from the added external salts^25,58,59^ (see Figure 3c-d). In all formulations, the extent of the downshifting trend increases with increasing mIEC. This downshifting trend with increasing external salt concentration results in the decreased p*K*_a_. The details will be discussed in the next section.

### 4.3 Degree of Ionization (α) and p*K*_a_ via the Modified Henderson–Hasselbalch Equation

In this section, we recorded the degree of ionization (*α*) and p*K*_a_ of AA–PEGDA series in dilute (DI water) and concentrated (1 M NaCl (aq)) solutions via (1) potentiometric (POT) titration of a polymer coupon with the strong base (NaOH) and (2) subsequent ATR–FTIR analysis. By integrating the dissociation data in both solution phase (via POT titration) and polymer phase (via ATR–FTIR analysis) together, we can thoroughly monitor the dissociation process and quantify the dissociation parameters (*α* and p*K*_a_) in the polymers. The modified Henderson–Hasselbalch equation was used to fit the data and describe the physical picture of the dissociation in the polymers.

Figure 4a and c reports the degree of ionization (*α*_*pH*_) vs pH of AA–PEGDA series in dilute (DI water) and concentrated (1 M NaCl (aq)) solutions via POT titration, whereas Figure 4b and d shows the *α_IR_* vs pH results via ATR–FTIR analysis (also see Figure S17). In each figure, symbols are experimental data, while solid lines are the best fit using the modified Henderson–Hasselbalch equation. In all formulations, as the external pH increases between 3 and 12, the degree of ionization (*α*) increases between 0 and 1, following the modified Henderson–Hasselbalch equation. At pH > 10–11, all AA–PEGDA series are fully dissociated (*α* = 1). In general, the *α* vs pH trends obtained via both POT titration and ATR–FTIR analysis are consistent with each other. This indicates that both methods faithfully describe the physical picture of the dissociation process in the polymers. Also, the *α* vs pH trends are aligned well with the modified Henderson–Hasselbalch equation. This confirms that our dissociation process can be reasonably analyzed within the framework of the modified Henderson–Hasselbalch equation. Using the Henderson–Hasselbalch equation (eq 15), pH vs log­(*α*/(1 – *α*)) was plotted, and straight lines were obtained for all compositions, as shown in Figure S18. At the halfway point (*α* = 0.5), p*K*_a_ value was determined (eq 27) and the slope of pH vs log­(*α*/(1 – *α*)) plot was the fitting parameter (*B*). Detailed analyses of other formulations are shown in Figures S15–S16. The degree of ionization (*α*), p*K*_a_, and fitting parameter (*B*) values are summarized in Table 1. Detailed ATR–FTIR analyses of all formulations are provided in Section S2 and Figures S9–S14. Note that relatively large error bars in ATR–FTIR analysis (see Figure 4b and d) are attributed to the absorbance peak deconvolution method, as reported in our previous report^32^ and elsewhere.^23^

**Figure 4:**
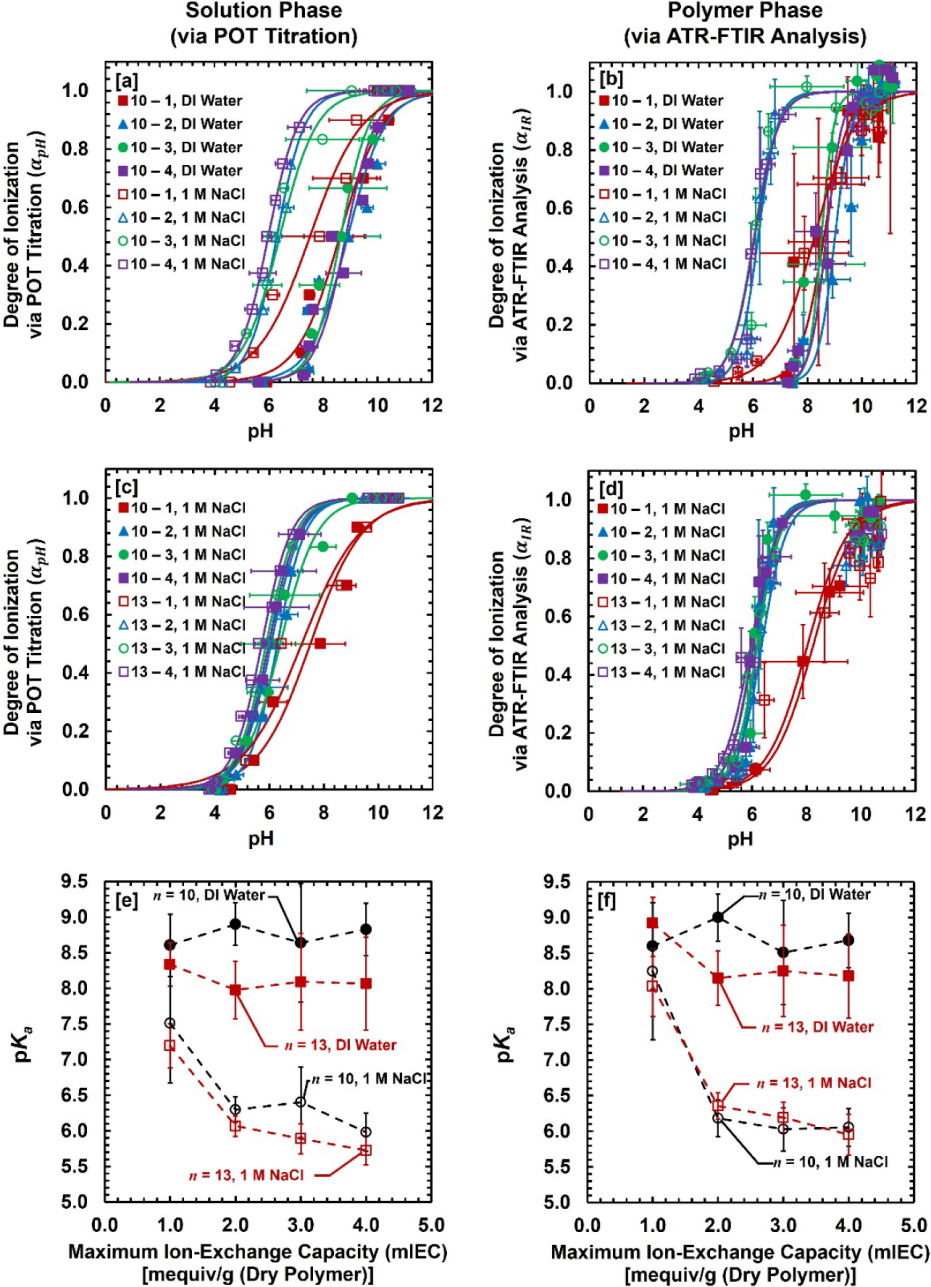
**[a, c]** Degree of ionization (*α*_pH_) vs pH of AA–PEGDA series via POT titration. Symbols are experimental data, while solid lines are the best fit using the modified Henderson–Hasselbalch equation. **[a]** *n* = 10 series in DI water and 1 M NaCl (aq) solution. **[c]** *n* = 10 and *n* = 13 series in 1 M NaCl (aq) solution. **[b, d]** Degree of ionization (*α_IR_*) vs pH of AA–PEGDA series via ATR–FTIR analysis. **[b]** *n* = 10 series in DI water and 1 M NaCl (aq) solution. **[d]** *n* = 10 and *n* = 13 series in 1 M NaCl (aq) solution. **[e, f]** p*K*_a_ vs the maximum ion-exchange capacity (mIEC) of AA–PEGDA series in DI water and 1 M NaCl (aq) solution. p*K*_a_ was determined via **[e]** POT titration and **[f]** ATR–FTIR analysis. Dashed lines are used to guide the eyes. Error bars are included. Error bars represent the standard deviation of at least three independent replicate samples.

**Table 1:** p*K*_a_ and *B* Values of AA–PEGDA Series in DI Water and 1 M NaCl (aq) Solution Determined Using the Modified Henderson–Hasselbalch Equation via POT Titration and ATR–FTIR Analysis^a^

	Dilute Solution (DI water)				Concentrated Solution (1 M NaCl (aq) solution)			
	POT titration		ATR–FTIR analysis		POT titration		ATR–FTIR analysis	
Sample [*n*– mIEC]	p*K*_a_	*B*	p*K*_a_	*B*	p*K*_a_	*B*	p*K*_a_	*B*
10–1	8.60 ± 0.43	1.80 ± 0.03	8.60 ± 0.61	1.02 ± 0.37	7.51 ± 0.84	2.21 ± 0.60	8.25 ± 0.96	1.72 ± 0.45
10–2	8.90 ± 0.30	1.59 ± 0.13	9.00 ± 0.33	0.71 ± 0.07	6.30 ± 0.18	1.18 ± 0.03	6.18 ± 0.26	0.82 ± 0.09
10–3	8.64 ± 0.83	1.19 ± 0.30	8.51 ± 0.73	0.52 ± 0.13	6.40 ± 0.49	1.56 ± 0.40	6.03 ± 0.30	1.01 ± 0.15
10–4	8.83 ± 0.37	1.26 ± 0.00	8.68 ± 0.38	0.68 ± 0.07	5.98 ± 0.27	1.26 ± 0.12	6.05 ± 0.27	1.01 ± 0.08
13–1	8.34 ± 0.30	1.85 ± 0.02	8.92 ± 0.36	1.33 ± 0.11	7.19 ± 0.31	2.47 ± 0.03	8.03 ± 0.42	1.71 ± 0.11
13–2	7.98 ± 0.40	1.23 ± 0.10	8.15 ± 0.38	0.56 ± 0.07	6.07 ± 0.14	1.27 ± 0.07	6.35 ± 0.18	1.03 ± 0.06
13–3	8.09 ± 0.68	0.96 ± 0.30	8.25 ± 0.64	0.56 ± 0.16	5.89 ± 0.21	1.25 ± 0.04	6.19 ± 0.22	0.98 ± 0.07
13–4	8.07 ± 0.65	1.02 ± 0.39	8.18 ± 0.59	0.62 ± 0.20	5.73 ± 0.21	1.24 ± 0.14	5.95 ± 0.29	1.16 ± 0.11

a An average value with a standard deviation is reported for each measurement with at least three independent replicate samples.

Figure 4e and f summarizes the p*K*_a_ values in the dilute (DI water) and concentrated (1 M NaCl (aq)) solutions determined at the halfway point via both methods. The p*K*_a_ values in AA–PEGDA series show consistent trends as a function of polymer composition (mIEC and *n*) and external salt conditions. First, as mIEC increases (all other conditions remain the same, including a fixed PEGDA cross-linker length, *n*, and a fixed external salt solution), the degree of ionization (*α*) vs pH curves shift to the left, corresponding to the decreased p*K*_a_ in the polymers. The extent of the decreased p*K*_a_ trend is relatively smaller in the dilute solution (DI water) and is greater in the concentrated solution (1 M NaCl (aq)). In AA–PEGDA series, as mIEC increases (AA monomer content increases), PEGDA cross-linker content decreases, and thus, cross-linking density (*v_t_*) decreases, resulting in a looser polymer network with a higher water swelling, as reported in our previous work.^22,32^ A higher water swelling in a looser polymer network offers a more favorable environment for dissociation, leading to a lower p*K*_a_.

The extent of decreasing p*K*_a_ trend is greater in the concentrated solution (1 M NaCl (aq)) due to the enhanced electrostatic screening effect by the external salts, providing a favorable environment for dissociation (lowering p*K*_a_) by suppressing electrostatic repulsion. In the dilute solution (DI water), the extent of the decreasing p*K*_a_ trend is relatively smaller within the uncertainty of a measurement because of the suppressed dissociation (smaller change in p*K*_a_) originating from the enhanced electrostatic repulsion in the absence of external salts. In particular, the p*K*_a_ values of *n* = 10 series titrated in DI water exhibit slight rise and fall within the uncertainty of measurement. This is likely due to the tighter polymer network nature in *n* = 10 series combined with the dilute solution effect. Because *n* = 10 series has a tighter network (with a shorter PEGDA cross-linker), it shows smaller shifts in the *α* vs pH curves, corresponding to the smaller changes in p*K*_a_ values within the uncertainty of measurement.

Second, as PEGDA cross-linker length (*n*) increases (other conditions are the same in a fixed mIEC and a fixed external salt solution), p*K*_a_ values shift to a lower range because of the enhanced dissociation in a looser polymer network. A longer PEGDA cross-linker forms a looser polymer network with a lower cross-linking density and higher water swelling. A water-rich environment provides a favorable environment for dissociation. A higher water content in the polymer also increases the distance between charged groups and thus reduces the electrostatic repulsion. Both effects lead to lower p*K*_a_ ranges in the looser network (more details will be discussed in the following section).

The extent of the decreased p*K*_a_ range between *n* = 10 and *n* = 13 series is relatively smaller in the concentrated solution (1 M NaCl (aq)) than in the dilute solution (DI water). This indicates that the external salt effect is a dominant factor in our dissociation process. As a future work, a longer PEGDA cross-linker (*n* > 13) can be synthesized and used to expand the data sets.

Third, in a fixed polymer composition (mIEC and *n* are fixed), p*K*_a_ values shift to a lower range in the concentrated solution. As the external salt concentration increases, the electrostatic repulsion between dissociated COO^–^ groups is weakened by electrostatic screening effects.^25,58,59^ This leads to the greater shifts in *α* vs pH curves, corresponding to the larger decrease in p*K*_a_ values (see Figure 4a-b). Overall, the decreassing degree of p*K*_a_ values is greater and more obvious in the concentrated solution (see Figure 4e-f). This indicates the importance of external salt effects on the dissociation process in our system. In the concentrated solution, the decreasing p*K*_a_ trend with respect to mIEC (the total dissociable COOH groups in a polymer) is more obvious, since the electrostatic repulsion between the dissociated COO^–^ groups is suppressed by the external salts. More details are discussed in Section 4.5.

Fitting parameter (*B*) from the modified Henderson–Hasselbalch equation does not have fundamental physical origin, but it reflects the extent of deviation from the ideal Henderson–Hasselbalch equation^28^ (see Figure S19). If *B* = 1, the modified Henderson–Hasselbalch equation returns back to the original Henderson–Hasselbalch equation. If *B* departs from 1, some literature have correlated the degree of deviation with chemical and/or morphological features in ion-exchange resins.^66–68^ Some reports relate *B* values to their degree of nonideality in a system with respect to an ideal solution analogue.^28,68^ Although the physical meaning of *B* value in weakly charged polymers is still under debate and speculative, it was of interest to record the general trend of *B* values in AA–PEGDA series.

First, our *B* values in this study (*B* = 0.5–2.5) are in similar ranges (*B* = 1.0–2.7) of other acrylic acid (AA)-based polymers in literature^28–30,66–68^ (see Table 1). Second, *B* values by POT titration are relatively higher than those by ATR–FTIR analysis, within the uncertainty of measurement. The slight difference in *B* values via the two methods could be attributed to the differences in measurement methods. Third, as mIEC increases (with a fixed PEGDA cross-linker length, *n*), *B* values generally decrease in both dilute and concentrated salt solutions, following the similar trend in p*K*_a_ values (p*K*_a_ values decrease with increasing mIEC). In contrast, as PEGDA cross-linker length (*n*) increases, no clear trend in *B* values was observed with varying *n*. Similarly, different external salt solutions (DI water vs 1 M NaCl (aq)) show no significant influence on *B* values. Difficulties in correlating *B* values with polymer compositions and test conditions have been similarly reported by other researchers.^28–30,66–68^

### 4.4 Effects of Charged Group Content (*C*_*C*_^*m*^), Cross-linking Density (*v_t_*), and Water Content (*ϕ_w_*) on Dissociation (p*K*_a_)

To determine governing molecular parameters on dissociation process in AA–PEGDA series, we recorded the effects of charged group concentration $\left(C_{C}^{m}\right)$ , cross-linking density (*v_t_*), and equilibrium water content (*ϕ_w_*) on the dissociation parameters (p*K*_a_ and *B*), as shown in Figures 5, 6, and S20. Our motivation for introducing charged group concentration $\left(C_{C}^{m}\right)$ (see eq 8) comes from the fact that changing mIEC (AA monomer content) simultaneously alters PEGDA cross-linker content, leading to different cross-linking densities (*v_t_*) and water swelling (*ϕ_w_*) in the polymers. Varying external salt concentration (DI water vs 1 M NaCl (aq)) also changes equilibrium water swelling (*ϕ_w_*) in the polymers due to osmotic deswelling. Therefore, these molecular parameters (mIEC, $C_{C}^{m}$ , *v_t_*, and *ϕ_w_*) are coupled and need to be understood cooperatively in our AA–PEGDA series. Although we recorded the equilibrium water swelling (*ϕ_w_*) vs pH in the dilute (DI water) and concentrated (1 M NaCl (aq)) solutions, we only included *ϕ_w_* values at the lowest pH (pH = 3–6) and the highest pH (pH = 10–12) to show the water content ranges in our analysis, as shown in Table S3.^22,32^ In Figure 5, we used the highest water swelling (*ϕ_w_*) values at the highest pH (pH = 10–12) where all compositions are fully ionized (*α* = 1). The molecular parameters $(C_{C}^{m}$ , *v_t_*, and *ϕ_w_*) of all formulations at the lowest pH (pH = 3–6) are also shown in Figures S21–S22 and summarized in Table S3.

**Figure 5:**
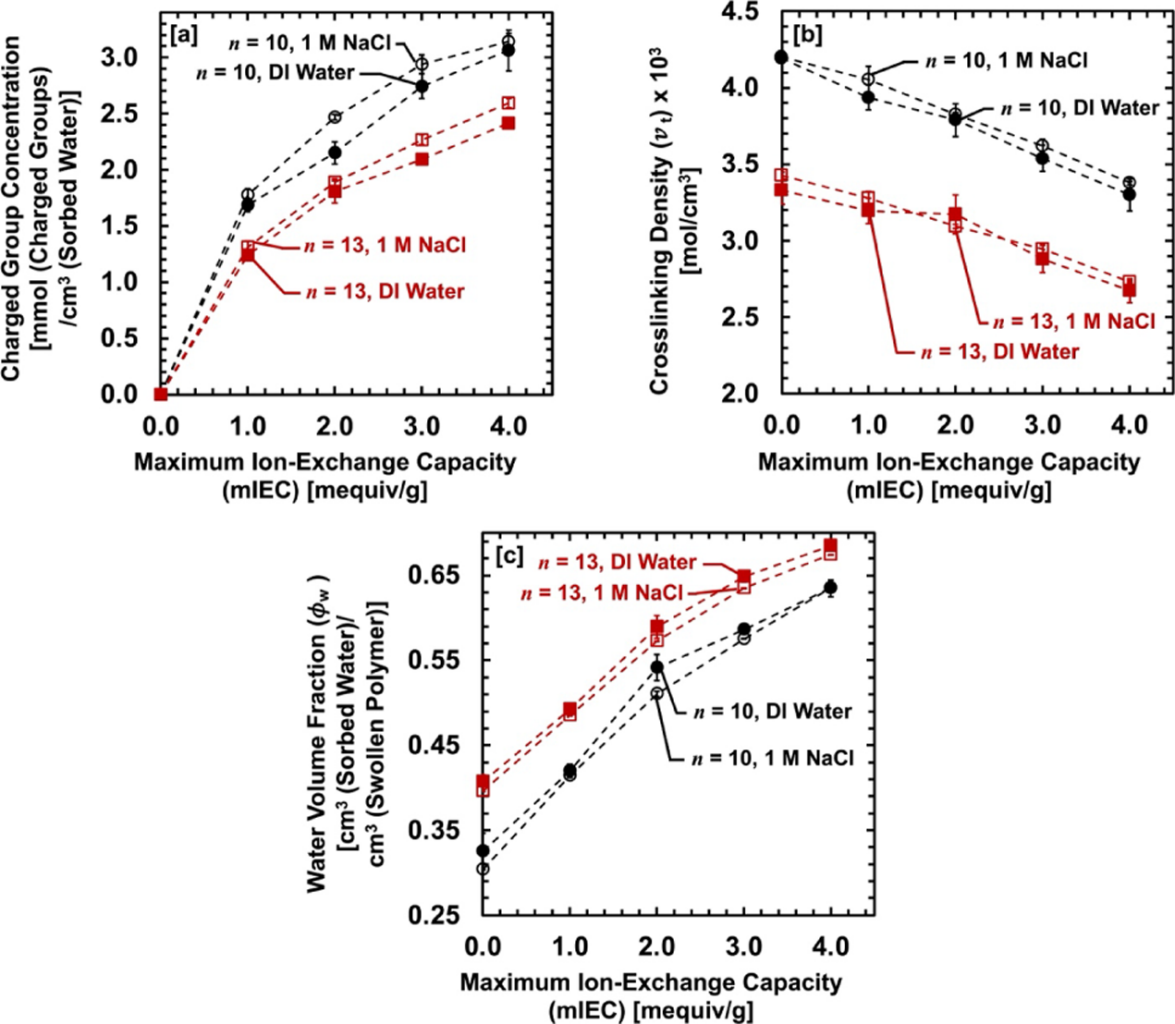
Effect of the maximum ion-exchange capacity (mIEC) on **[a]** charged group concentration $\left(C_{C}^{m}\right)$ , **[b]** cross-linking density (*v_t_*), and **[c]** water volume fraction (*ϕ_w_*) of AA–PEGDA series in DI water and 1 M NaCl (aq) solution at the highest pH (pH = 11–12). Dashed lines are used to guide the eyes. Error bars are included. Error bars represent the standard deviation of at least three independent replicate samples.

**Figure 6:**
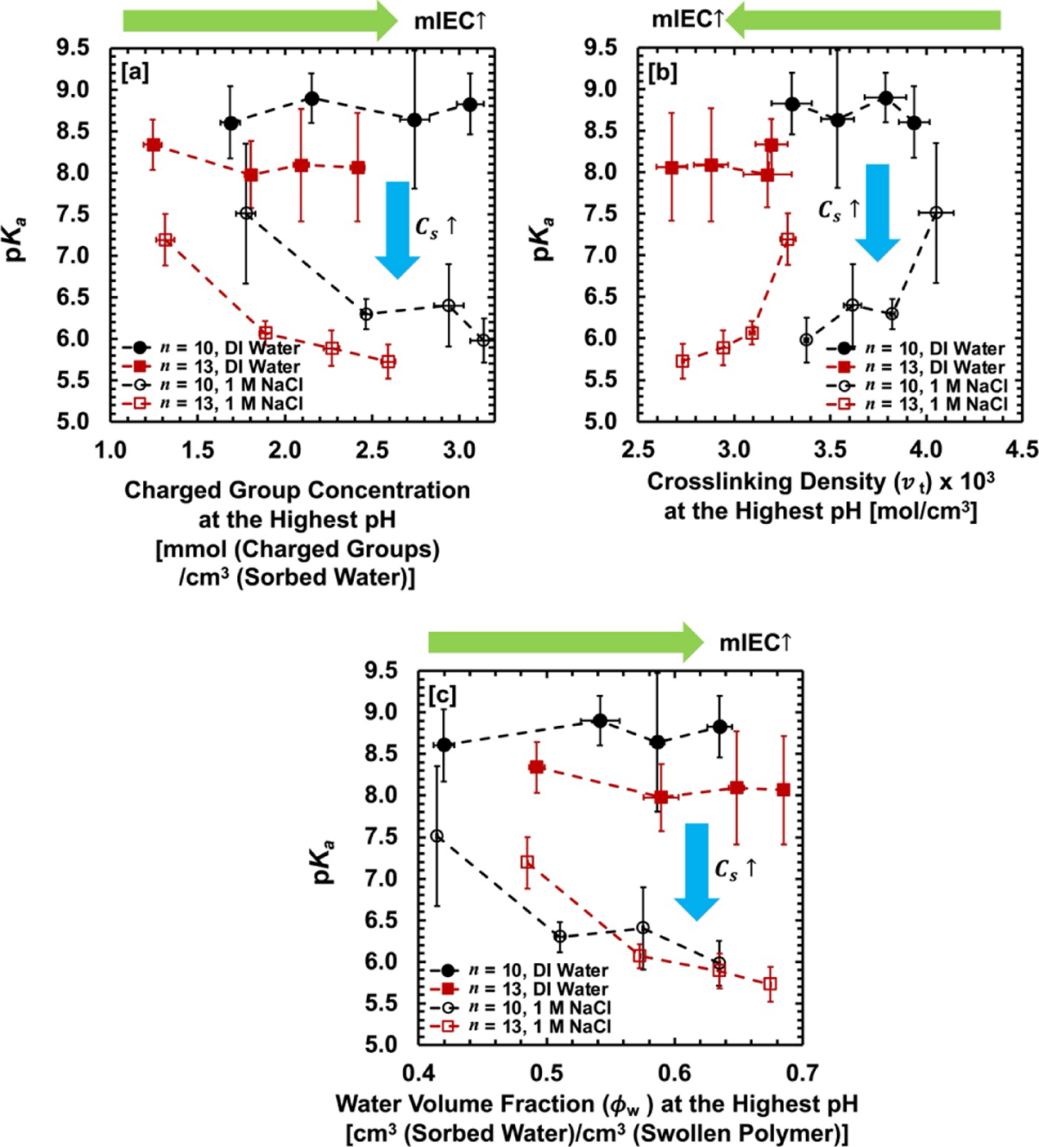
p*K*_a_ (via POT titration) vs **[a]** charged group concentration $\left(C_{C}^{m}\right)$ , **[b]** cross-linking density (*v_t_*), and **[c]** water volume fraction (*ϕ_w_*) of AA–PEGDA series at the highest pH (pH = 11∼12) in DI water and 1 M NaCl (aq) solution. Dashed lines are used to guide the eyes. Error bars are included. Error bars represent the standard deviation of at least three independent replicate samples.

In Figure 5, with a fixed PEGDA cross-linker length (*n*) and a fixed external salt solution, as mIEC increases (higher AA content), charged group concentration $\left(C_{C}^{m}\right)$ increases (see Figure 5a), cross-linking density (*v_t_*) decreases (see Figure 5b), and equilibrium water swelling (*ϕ_w_*) increases (see Figure 5c) in both dilute (DI water) and concentrated (1 M NaCl (aq)) solutions. First, as mIEC increases (with increasing AA monomer content) between 0 mequiv/g and 4 mequiv/g, charged group concentration $\left(C_{C}^{m}\right)$ follows the increasing trend of mIEC and increases between 0 mmol/cm^3^ and 3 mmol/cm^3^. Charged groups in a polymer prefer to be surrounded by water molecules until the elastic force by the polymer matrix reaches equilibrium with the osmotic pressure of water.^69^ The increased amount of charged groups $\left(C_{C}^{m}\right)$ raises the sorbed water content, leading to a higher water swelling (*ϕ_w_*) in the polymers. Additionally, as mIEC increases, PEGDA cross-linker content also decreases in a polymer, forming a looser polymer network with a lower cross-linking density (*v_t_*) (see Figure 5b). This lower *v_t_* increases water swelling (*ϕ_w_*) in the looser network. Thus, in our AA–PEGDA series, as mIEC increases (i.e., PEGDA cross-linker content decreases), equilibrium water swelling (*ϕ_w_*) increases because of higher $C_{C}^{m}$ and lower *v_t_* in the polymers (see Figure 5c).

Second, in a fixed mIEC and a fixed external salt solution, as PEGDA cross-linker length (*n*) increases, cross-linking density (*v_t_*) decreases, and water swelling (*ϕ_w_*) increases in a looser polymer network. These combined effects (lower *v_t_* and higher *ϕ_w_*) decrease the charged group concentration $\left(C_{C}^{m}\right)$ in the looser polymer network.

Third, in a fixed polymer composition (mIEC and *n* are fixed), as external salt concentration increases (DI water vs 1 M NaCl (aq)), water swelling (*ϕ_w_*) slightly decreases due to an osmotic deswelling effect (see Figure 5c). A lower water swelling (*ϕ_w_*) in the polymer moderately increases the charged group concentration $\left(C_{C}^{m}\right)$ in the 1 M NaCl (aq) solution.

Built on these general trends of molecular parameters (mIEC, $C_{C}^{m}$ , *v_t_*, and *ϕ_w_*) in AA–PEGDA series, Figure 6 shows the effects of charged group concentration $\left(C_{C}^{m}\right)$ , cross-linking density (*v_t_*), and equilibrium water swelling (*ϕ_w_*) on p*K*_a_ values (via POT titration) in dilute and concentrated salt solutions. In our AA–PEGDA series, these coupled molecular parameters (mIEC, $C_{C}^{m}$ , *v_t_*, and *ϕ_w_*) provide competing effects on the dissociation process (*α* and p*K*_a_) in the polymers.^32^ (1) First, as mIEC increases (higher AA monomer content), charged group concentration $\left(C_{C}^{m}\right)$ increases and PEGDA cross-linker content decreases in AA–PEGDA series. Both effects (increased $C_{C}^{m}$ and decreased *v_t_*) increase equilibrium water swelling (*ϕ_w_*) in the polymers (see Figure 5c). A water-rich environment in swollen polymers favors dissociation via offering an increased dielectric constant medium. High water swelling also increases the distance between charged groups and thus decreases the electrostatic repulsion.^25,27,62–65^ Moreover, decreased cross-linking density (*v_t_*) (from decreased PEGDA cross-linker content) provides more flexible chain configuration, providing a more favorable environment for dissociation. As a result (increased mIEC, increased $C_{C}^{m}$ , decreased *v_t_*, and increased *ϕ_w_*), p*K*_a_ decreases if other conditions remain the same.

(2) On the other hand, if the increasing degree of water swelling (*ϕ_w_*) (via increased charged group content) is not significant, as opposed to our system, then the dissociation behavior is different. In this case, as mIEC increases, the distance between charged groups decreases, and thus, the electrostatic repulsion between the charged groups increases. These combined effects (increased mIEC, increased $C_{C}^{m}$ , but moderately similar *ϕ_w_* range) suppress dissociation, leading to a higher p*K*_a_.^62–65^

In our AA–PEGDA series, as mIEC increases (charged group content $\left(C_{C}^{m}\right)$ increases), p*K*_a_ values generally decrease, as shown in Figure 6a, confirming that the former is the case. As mIEC increases, cross-linking density (*v_t_*) decreases, and p*K*_a_ decreases (see Figure 6b). Simultaneously, as cross-linking density (*v_t_*) decreases, water volume fraction (*ϕ_w_*) increases, and thus, p*K*_a_ decreases (see Figure 6c). Therefore, in our system, as mIEC increases, the decreased cross-linking density (*v_t_*) and simultaneously increased water volume fraction (*ϕ_w_*) reduce the overall electrostatic repulsion, resulting in decreased p*K*_a_.

The p*K*_a_ trend is likely to be similar in all compositions but more obvious in a looser *n =* 13 series and in the concentrated salt solution (1 M NaCl (aq)). Because *n* = 10 series has a tighter polymer network than that of *n* = 13 series, *n* = 10 series shows smaller shifts in *α* vs pH curves and, thus, smaller changes in p*K*_a_ values within the uncertainty of a measurement. In the concentrated salt solution, all formulations exhibit greater shifts in *α* vs pH curves due to the electrostatic screening effects from the external salts. This leads to larger shifts in p*K*_a_ values than those in the dilute solution (DI water), as consistent with previous reports.^25,29,30^ The p*K*_a_ trends from the ATR–FTIR analysis also show a similar trend, as shown in Figure S20.

Fitting parameter (*B*), representing the degree of deviation from the ideal Henderson–Hasselbalch equation, also shows similar trends as those of p*K*_a_, although the physical origin of *B* value is still under debate and speculative.^28,30,70^ In Figure S23, *B* values are plotted against charged group concentration $\left(C_{C}^{m}\right)$ , cross-linking density (*v_t_*), and water volume fraction (*ϕ_w_*). Similar to the p*K*_a_ trends, as mIEC increases, the combined effects of higher $C_{C}^{m}$ , lower *v_t_* and higher *ϕ_w_* lead to lower *B* values, closer to 1 via POT titration (see Figure S23a, c, e). Different external salt solutions (DI water vs 1 M NaCl (aq)) show no significant influence on *B* values, as reported in the previous section and in other reports.^30,70^

### 4.5 Molecular-Scale Picture of Dissociation via Relevant Length Scales

To describe the molecular picture of dissociation process in AA–PEGDA series, we compared relevant molecular-level physical length scales in our system. Our p*K*_a_ values (via POT titration) are plotted against (1) the average distance between dissociated charged groups in a swollen polymer (*r_c_*, subscript *c* notes charged groups) (at the highest pH = 10–12) (see Figure 7a), (2) the average distance between salt ions in an external solution (*r_ion_*) (see Figure 7b), and the respective (3) Bjerrum length (*l_B_*) (see Figure 7c) and (4) the Debye screening length *r_D_*) in a system (see Figure 7d). The motivation for plotting p*K*_a_ vs these four length scales (*r_c_*, *r_ion_*, *l_B_*, and *r_D_*) comes from the fact that the electrostatic interaction originating from varying polymer compositions (mIEC, $C_{C}^{m}$ , *v_t_*, and *ϕ_w_*) and external salt conditions (dilute vs concentrated salt solutions) governs dissociation process. Some varying parameters (in polymer compositions and external salt conditions) offer opposite and/or competing influences on electrostatic interaction and, thus, dissociation. Therefore, to determine the governing molecular parameters on the electrostatic interaction (thus, dissociation) in our system, our system variables (via changing polymer compositions and external salt conditions) are first expressed using two important length scales, i.e., (1) the average distance between charged groups in a polymer (*r_c_*) (see Figure 7a) and (2) the average distance between salt ions in an external salt solution (*r_ion_*) (see Figure 7b) to describe the physical picture of the system. Relative standing of these two length scales with respect to each other broadly dictates the magnitude and direction of electrostatic interaction in the system.

**Figure 7:**
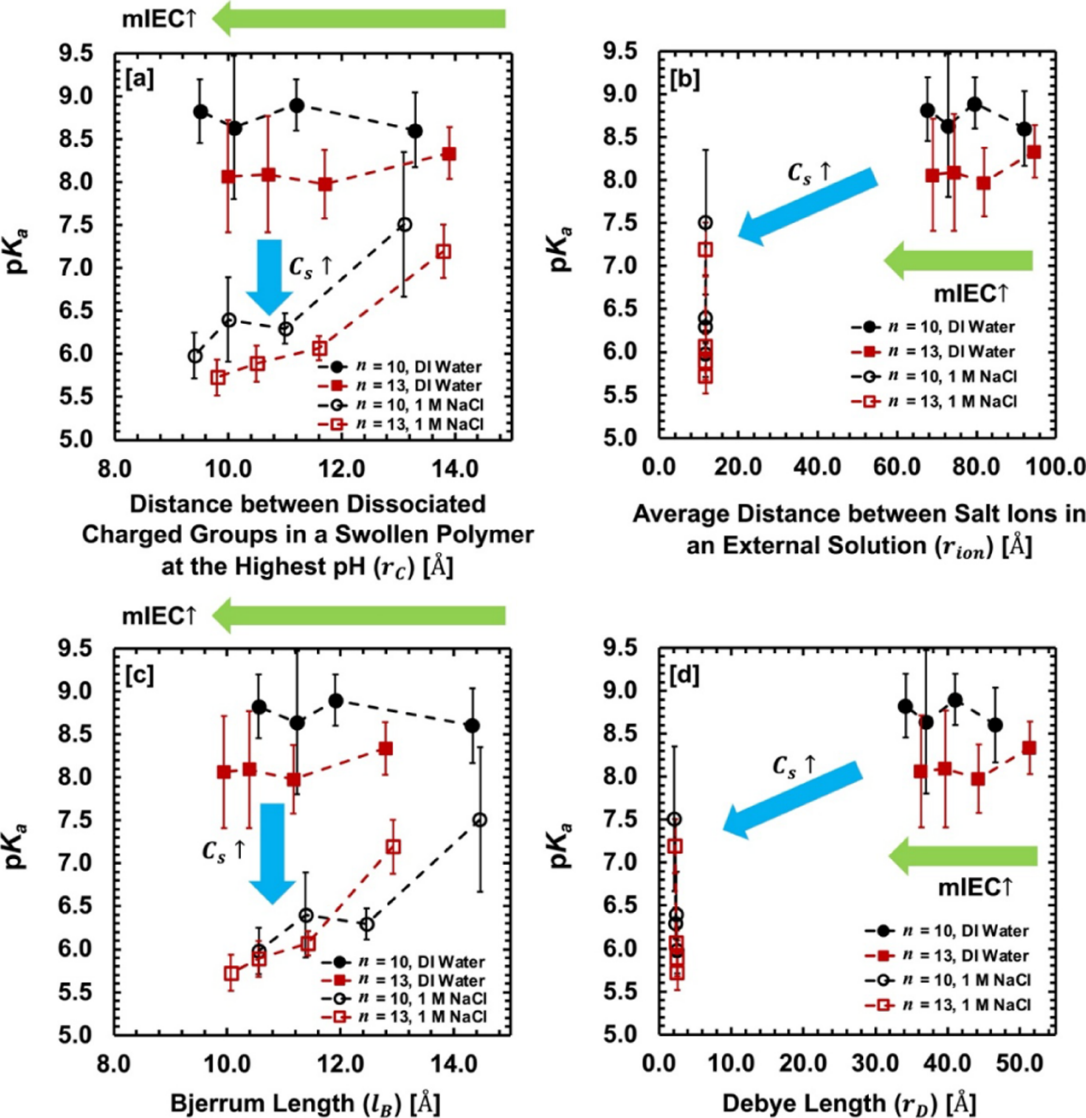
p*K*_a_ (via POT titration) vs **[a]** the average distance between dissociated charged groups in a swollen polymer (*r_c_*) at the highest pH (pH = 11–12), **[b]** the average distance between salt ions in an external solution (*r_ion_*), **[c]** Bjerrum length (*l_B_*) and **[d]** Debye screening length (*r_D_*) in AA–PEGDA series in DI water (filled symbols) and 1 M NaCl (aq) solution (unfilled symbols). Dashed lines are used to guide the eyes. Error bars are included.

In addition, the respective (3) Bjerrum length (*l_B_*) (see Figure 7c) and (4) the Debye screening length (*r_D_*) (see Figure 7d) are used to interpret the electrostatic phenomena. In this system, Bjerrum length (*l_B_*) indicates the strength of electrostatic interaction between the charged group in a polymer and the surrounding mobile ions in an external solution. Debye screening length (*r_D_*) represents the distance at which the electrostatic interaction by the charged group in the polymer is effectively screened by the mobile ions in the external solution. These four length scales (*r_c_*, *r_ion_*, *l_B_*, and *r*_D_) need to be discussed cooperatively for a comprehensive understanding.

First, in dilute solution (DI water), as mIEC increases from mIEC = 1 mequiv/g to mIEC = 4 mequiv/g (i.e., with increasing $C_{C}^{m}$ , decreasing *v_t_*, and increasing *ϕ_w_*), the average distance between dissociated charged groups in a swollen polymer (at the highest pH = 10–12) decreases from *r_c_* = 13∼14 Å to *r_c_* = 10 Å (i.e., suppressing dissociation via enhanced electrostatic repulsion) (see Figure 7a), and its corresponding Bjerrum length (*l_B_*) decreases from *l_B_* = 14∼13 Å to *l_B_* = 11∼10 Å (see Figure 7c) in the polymers. In *n* = 10 series, the charged group distance (*r_c_* = 13∼10 Å) is shorter than the Bjerrum length (*l_B_* = 14∼11 Å), whereas, in *n* = 13 series, the charged group distance (*r_c_* = 14∼10 Å) is comparable to the Bjerrum length (*l_B_* = 13∼10 Å). If the distance is shorter than (or comparable to) the Bjerrum length (*r* ≲ *l_B_*), the electrostatic interaction energy is larger than the thermal energy. Subsequently, the ion is strongly associated with the charged group, suppressing the dissociation. Both length scales (*r_c_*, *l_B_*) of the polymers dictate the p*K*_a_ values to be in a higher range.

In the dilute solution, as mIEC increases, the average distance between salt ions in DI water decreases from *r_ion_* = 92∼95 Å to *r_ion_* = 68∼69 Å with increasing total amount of strong base (NaOH) for titration from 0.002 M to 0.005 M (see Figure 7b). The distance between charged groups (*r_c_* = 14∼10 Å) in the polymers is significantly shorter than the distance between salt ions (*r_ion_* = 95∼68 Å) in the DI water. This indicates that the amount of external salts is not sufficient to modulate the enhanced electrostatic repulsion between charged groups in the polymers. This is consistent since the corresponding Debye screening length (*r_D_*) is in the range of 51∼34 Å (see Figure 7d), which is longer than the charged group distance (*r_c_* = 14∼10 Å), but shorter than the spacing between salt ions (*r_ion_* = 95∼68 Å) (i.e., *r_c_* < *r_D_* < *r_i__on_*). Below the Debye screening length (*r* < *r_D_*), the electrostatic interaction energy exerted by the charged group (in the polymer) is dominant over the thermal energy. Consequently, the electrostatic interaction energy regulates and influences the movement and arrangement of nearby mobile ions, overcoming the random thermal motion. Thus, the dominant electrostatic interaction energy suppresses the dissociation, pushing p*K*_a_ to a higher value.

Overall, in the dilute solution (DI water), the relative order of these four length scales (*r_c_* ≲ *l_B_* ≪ *r_D_* < *r_ion_*) indicates that the electrostatic interaction is the dominant factor for suppressing the dissociation in AA–PEGDA series and thus moving p*K*_a_ to a higher range. Under this fixed dilute condition, the p*K*_a_ values slightly decrease (from p*K*_a_ = 8.60∼8.34 to p*K*_a_ = 8.83∼8.07) with increasing mIEC (1∼4 mequiv/g) due to the increased water swelling (*ϕ_w_*) (favorable for dissociation), but the extent of the decreased p*K*_a_ range is very small (see Figure 7c). This again confirms that electrostatic interaction governs (suppresses) the dissociation process in the dilute solution. The effect of high water swelling (*ϕ_w_*) on lowering p*K*_a_ is less pronounced in the dilute solution.

Second, in the concentrated salt solution (1 M NaCl (aq)), as mIEC increases from mIEC = 1 mequiv/g to mIEC = 4 mequiv/g, the average distance between charged groups in a swollen polymer decreases from *r_c_* = 13∼14 Å to *r_c_* = 9∼10 Å (see Figure 7a). The *r_c_* range is similar to those in the dilute solution (DI water) because of the similar (moderately smaller) water swelling (*ϕ_w_*) range in 1 M NaCl (aq) solution. Its respective Bjerrum length (*l_B_*) decreases from *l_B_* = 15∼13 Å to *l_B_* = 11∼10 Å (see Figure 7c) in the polymers. In *n* = 10 series, the charged group distance (*r_c_* = 13∼9 Å) is shorter than the Bjerrum length (*l_B_* = 15∼11 Å), whereas in *n* = 13 series, the charged group distance (*r_c_* = 14∼10 Å) is comparable to the Bjerrum length (*l_B_* = 13∼10 Å).

In the concentrated solution, as mIEC increases, the average distance between salt ions remains almost constant (*r_ion_* ∼ 12 Å) with increasing total amount of added strong base (NaOH) for titration from 0.002 M to 0.005 M (see Figure 7b). The charged group distance (*r_c_* = 14∼9 Å) in the polymers is comparable to the spacing between salt ions (*r_ion_* ∼ 12 Å) in the external solutions. This indicates that sufficient salt ions exist to screen and modulate the electrostatic repulsion between charged groups in the polymers. Consistently, the Debye screening length (*r_D_*) is in the range of 2.1–2.6 Å (see Figure 7c), which is substantially shorter than the charged group distance (*r_c_* = 14∼9 Å) in the polymers as well as the spacing between salt ions (i.e., *r_D_* < *r_c_* ≈ *r_ion_*) in the solutions. Beyond the Debye screening length (*r* > *r_D_*), the electrostatic interaction energy exerted by the charged group (in the polymer) is significantly weakened and screened by the redistribution of mobile ions in the external solution. Thus, the screened electrostatic interaction energy no longer suppresses the dissociation and, thus, significantly decreases p*K*_a_ values. This decreasing p*K*_a_ trend is consistent with other experimental and theoretical dissociation studies using polyelectrolyte brushes, polymer networks, and linear polymers in concentrated salt solution conditions.^54,71–75^

Overall, in the concentrated salt solution (1 M NaCl (aq)), the relative order of these four length scales (*r_D_* ≪ *r_c_* ≲ *l_B_* ≈ *r_ion_*) indicates that, the screened electrostatic interaction via added external salts is the governing factor for promoting dissociation in AA–PEGDA series and thus substantially lowering p*K*_a_. Under this fixed concentrated condition, the p*K*_a_ values further significantly decrease (from p*K*_a_ = 7.51∼7.19 to p*K*_a_ = 5.98∼5.73) with increasing mIEC (1∼4 mequiv/g) due to the increased water swelling (*ϕ_w_*) (favorable for dissociation). The extent of the decreasing p*K*_a_ trend (with respect to polymer compositions) is greater compared to the smaller p*K*_a_ changes (with varying mIEC and *n*) in the dilute solutions (DI water).

Figure 8 summarizes the molecular-level physical picture of dissociation (p*K*_a_) in both dilute solution (Figure 8a) and concentrated solution (Figure 8b) using the four relevant length scales (*r_c_*, *r_ion_*, *l_B_*, and *r_D_*). In the dilute solution (DI water), the electrostatic interaction energy is maximized in the absence of external salts and thus suppresses the dissociation and increases p*K*_a_. The charge distance in the polymers is closer than the Bjerrum length and is significantly shorter than the Debye screening length (*r_c_* ≲ *l_B_* ≪ *r_D_* < *r_ion_*). The unscreened electrostatic interaction is dominant over the thermal energy, and the ions are strongly associated with the charged groups (suppressing dissociation and increasing p*K*_a_). On the other hand, in the concentrated salt solution (1 M NaCl (aq)), the electrostatic interaction energy is screened and minimized via the added external salts. The charge distance in the polymers (*r_c_*) and the Bjerrum length (*l_B_*) are much longer than the Debye screening length (*r_D_* ≪ *r_c_* ≲ *l_B_* ≈ *r_ion_*). The screened electrostatic interaction no longer suppresses the dissociation and thus decreases p*K*_a_.

**Figure 8:**
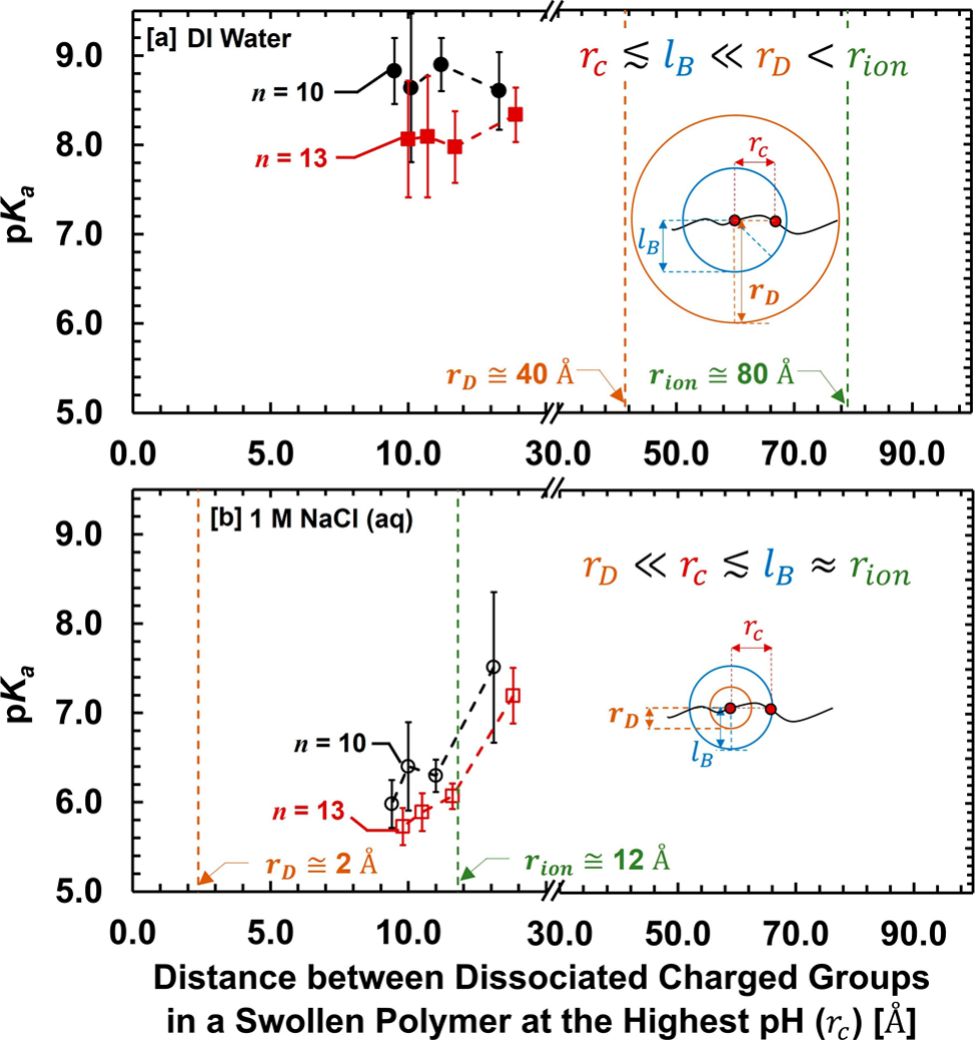
p*K*_a_ (via POT titration) vs the average distance between dissociated charged groups in a swollen polymer (*r_c_*) at the highest pH (pH = 11–12), along with the relevant length scales (*r_ion_*, *l_B_*, and *r_D_*) in **[a]** DI water (filled symbols) and **[b]** 1 M NaCl (aq) solution (unfilled symbols). Vertical dashed lines indicate the average Debye screening length (*r_D_*) and the average distance between salt ions in an external solution (*r_ion_*).

As a result, in the dilute solution (DI water), due to the enhanced electrostatic interaction, the influence of varying polymer composition (mIEC and PEGDA cross-linker length, *n*) has relatively smaller impact on dissociation and p*K*_a_. In contrast, in the concentrated salt solution (1 M NaCl (aq)) because of the screened electrostatic interaction via the added external salts, changing polymer composition (mIEC, *n*) shows greater influences on dissociation and p*K*_a_. If an external salt condition is fixed, varying polymer composition (mIEC, *n*) in AA–PEGDA series leads to different water swelling (*ϕ_w_*) (high *ϕ_w_* favors more dissociation), altering dissociation trend and p*K*_a_. Interestingly, in a fixed chemical composition (mIEC, *n* are fixed) of our system, as the external salt concentration varies between DI water and 1 M NaCl (aq) solution, the water swelling (*ϕ_w_*) range is relatively similar, although *ϕ_w_* slightly decreases in 1 M NaCl (aq) solution because of osmotic deswelling. This relatively similar water swelling (*ϕ_w_*) range in both DI water and 1 M NaCl (aq) solution indicates that the reduced electrostatic interaction via the added external salts primarily governs the dissociation and p*K*_a_ in our AA–PEGDA series. After the electrostatic interaction is effectively screened via the added external salts, then water swelling (*ϕ_w_*) in the polymers broadly dictates the dissociation as the next governing factor. Overall, varying external salt conditions and water swelling (*ϕ_w_*) ratios in the polymers are the major molecular parameters for controlling the dissociation behavior in weakly charged polymers. Our multiscale dissociation analysis of weakly charged polymers can help us understand the dissociation process in complex environments and thus achieve desired transport properties in broader membrane-based applications.

Note that we mainly used the p*K*_a_ values via POT titration for our analysis in this section. The p*K*_a_ values via ATR–FTIR analysis were also similarly plotted against the four relevant length scales, as shown in Figures S25–S26. The trends are consistent via both methods. In addition, the four relevant length scales were plotted as a function of polymer composition (mIEC, *n*) and external salt solution conditions, as shown in Figure S24 and summarized in Tables S4–S5.

### 4.6 Comparison with Other AA-Containing Polymers in the Literature: Effect of External Salt Conditions on Dissociation

In this last section, p*K*_a_ results of our AA–PEGDA series in dilute and concentrated salt solutions were compared with those of other acrylic acid (AA)-based polymers in the literature. Our goal was to build a comprehensive understanding of dissociation among similar weakly charged polymers. We also aimed to understand the dissociation behavior of our AA–PEGDA series in the context of other similar AA-based polymers. Toward this goal, Figure 9 shows p*K*_a_ vs cross-linking density (*v_t_*) results of our AA–PEGDA series, along with other data from linear poly­(acrylic acid) (PAA) polymers with different molecular weights (M̅_w_ = 2–450 kg/mol),^23^ and cross-linked PAA and poly­(methacrylic acid) (PMA) polymers in dilute and concentrated salt solutions.^29,30^ Although the polymer forms (e.g., dissolved polymer solutions, porous beads), cross-linker types and concentrations (e.g., divinylbenzene (DVB), PEGDA), and external salt solution conditions (e.g., salt type and concentrations) of these other polymers in the literature are different from those of our AA–PEGDA series in this study, these reports were selected because of their detailed information on polymer composition and *α* vs pH data for a reasonable comparison.

**Figure 9:**
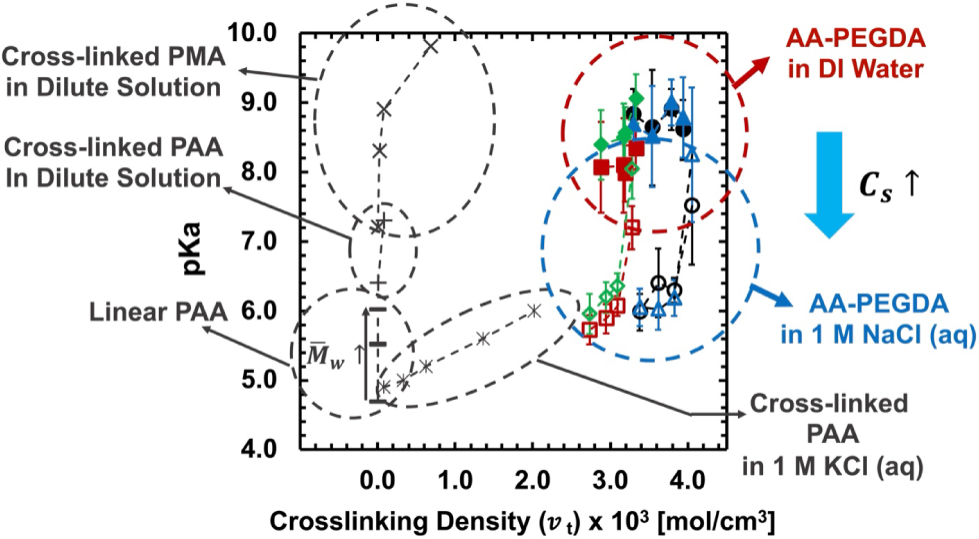
Comparison of AA–PEGDA series in this study with other acrylic acid (AA)-based polymers in the literature.

Note that our AA-PEGDA series was formed into free-standing thin films of uniform thickness and experiments were performed in dilute solutions (in DI water, NaOH *C_s_* = 0.002–0.005 M, and below the saturation limit, $C_{s}^{*})$ and concentrated solutions (in 1 M NaCl (aq) solution, where the total salt (NaCl and NaOH) concentration *C_s_* = 1.002–1.005 M). On the other hand, in the literature, linear PAA polymers were tested while being dissolved in dilute solutions (NaOH *C_s_* = 0.05–0.09 M). Cross-linked PAA and PMA polymers in porous beads were titrated in dilute solutions (*C_s_* = 0.03–0.06 M). Cross-linked PAA polymers were formed into porous beads (20–40 mesh beads) and tested in 1 M potassium chloride (KCl) (aq) solution.^23,29,30^

While the limitations are present (polymer forms, cross-linker type, and external solution conditions are not the same as our study) in the selected reports, the overall p*K*_a_ trend as a function of polymer chain length (*M̅_w_*), chain architecture (linear vs cross-linked), cross-linking density (*v_t_*), and external salt concentration (dilute vs salt solutions) can be found. We discussed the detailed comparison in our previous report.^32^ In general, (1) first, p*K*_a_ increases with increasing cross-linking density (*v_t_*), as shown in Figure 9. (2) Second, in linear polymer architecture (in PAA polymers), p*K*_a_ increases (p*K*_a_ = 5.41–5.86) with increasing polymer chain length (*M̅_w_* = 2–450 kg/mol) because of unfavorable dissociation on covalently bonded longer chains.^23,24^ Note that the p*K*_a_ of the AA monomer is in the range of 4.24–4.60.^23–26^ (3) Third, cross-linked PAA and PMA polymers show increased p*K*_a_ with increasing cross-linking density (*v_t_*) in both dilute and concentrated solution conditions. This is due to the unfavorable dissociation in restricted chain conformation by cross-linking.^29,30,66–68^ (4) Fourth, a higher external salt concentration decreases p*K*_a_ values in PAA polymers due to the increased electrostatic charge screening effect and, thus, the reduced electrostatic repulsion.^25,29,30^

Compared to the other AA-based polymers in the literature, our cross-linked AA–PEGDA series shows higher p*K*_a_ values in the dilute condition (DI water) because of higher cross-linking density and a lower p*K*_a_ range in the concentrated salt condition (1 M NaCl (aq)) due to the enhanced electrostatic charge screening effect by the added external salts, as shown in Figure 9. The dissociation behavior is consistent with the general trend of other AA-based polymers. Interestingly, p*K*_a_ range in the concentrated salt condition of some AA–PEGDA series is comparable to p*K*_a_ range of high *M̅_w_* linear AA polymers. Although a direct comparison requires a detailed further analysis, this comparable p*K*_a_ range via different molecular parameters (cross-linked vs linear polymer) and external salt conditions demonstrates the cooperative influences of polymer parameters and external salt effects on dissociation of the polymers. To the best of our knowledge, this is the first time that the dissociation process has been systematically studied using a thin-film form with systematically varied polymer composition and external salt conditions. Our AA–PEGDA system can serve as a useful platform to understand the molecular-level physical picture of the dissociation process of weakly charged polymers. Using this platform, our subsequent paper will further discuss the effect of external salt concentration on the dissociation process using a fixed chemical structure in order to advance our understanding of dissociation.

In this paper, we reported the dissociation process of weakly charged AA–PEGDA series in dilute and concentrated salt solutions via probing the dissociation in both 1) a solution phase (POT titration) and 2) a polymer phase (ATR–FTIR analysis). The pre-titration analysis confirmed that our AA–PEGDA series shows a very small dissociation degree (*α* = 0–4 %) before adding a strong base. While the extent of dissociation degree is very small, the dissociation trend is consistent with respect to polymer compositions and external salt conditions. When titrated, the dissociation process in our AA–PEGDA series follows the modified Henderson–Hasselbalch equation. Both POT titration (probing a solution phase) and ATR–FTIR analysis (detecting a polymer phase) describe the dissociation process well, showing similar ranges of dissociation parameters (*α*, p*K*_a_). The overall dissociation trend was well described by comparing four relevant molecular-level physical length scales (*r_c_*, *r_ion_*, *l_B_*, and *r_D_*) in our system.

In the dilute solution (DI water), the enhanced electrostatic interaction (*r_c_* ≲ *l_B_* ≪ *r_D_* < *r_ion_*) is the dominant factor for suppressing dissociation and thus moving p*K*_a_ to a higher range. The influence of varying polymer composition (mIEC and PEGDA cross-linker length, *n*) has a relatively smaller impact on dissociation and p*K*_a_ in the dilute condition. In contrast, in the concentrated salt solution (1 M NaCl (aq)), the screened electrostatic interaction (*r_D_* ≪ *r_c_* ≲ *l_B_* ≈ *r_ion_*) via the added external salts is the governing factor for promoting dissociation and thus substantially lowering p*K*_a_. Because of the screened electrostatic interaction, changing polymer composition (mIEC, *n*) shows greater influence on dissociation and p*K*_a_. If an external salt condition is fixed, varying polymer composition (mIEC, *n*) in AA–PEGDA series leads to different water swelling (*ϕ_w_*) (high *ϕ_w_* favors more dissociation), altering the dissociation trend and p*K*_a_. In our system, varying external salt conditions and water swelling (*ϕ_w_*) ratios in the polymers are the major molecular parameters for controlling the dissociation behavior and p*K*_a_. Our multiscale analysis of dissociation can help us understand the dissociation behavior of weakly charged polymers in complex environments and thus achieve desired transport properties. Ultimately, this system can offer a versatile foundation to design next-generation polymer membranes for sustainable technologies.

## 5 Conclusion

Charged polymer membranes serve as a versatile platform for a wide range of applications in energy, environment, and health because of their selective and controlled transport of small molecules across the membranes. To develop innovative charged polymer membranes, we previously designed a library of weak polyelectrolyte membranes, i.e., cross-linked acrylic acid (AA)–poly­(ethylene glycol) diacrylate (PEGDA) random copolymer networks (AA–PEGDA) with a wide ion-exchange capacity (IEC = 0–4 mequiv/g) range and limited water swelling. Weakly acidic acrylic acid (AA) monomer was chosen as a charged block. Poly­(ethylene glycol) diacrylate (PEGDA) cross-linkers with different molecular weights were used to control cross-linking densities in the networks and thus limit high water swelling. In this model polymer, in one fixed chemical structure, the charged group concentration can be systematically altered (degree of ionization, *α* = 0–1) as the external pH is varied (pH = 3–12). This library can help develop a mechanistic understanding of ion and water transport in charged polymer membranes.

Toward this goal, it is crucial to understand the dissociation process vs pH in weakly charged polymers. In this paper, we reported the dissociation process of weakly charged AA–PEGDA series in dilute and concentrated salt solutions via probing the dissociation in both 1) a solution phase (POT titration) and 2) a polymer phase (ATR-FTIR analysis). The pre-titration analysis confirmed that our AA–PEGDA series shows a very small dissociation degree (*α* = 0–4 %) before adding a strong base. While the extent of dissociation degree is very small, the dissociation trend is consistent with respect to polymer compositions and external salt conditions. When titrated, the dissociation process in our AA–PEGDA series follows the modified Henderson–Hasselbalch equation. Both POT titration (probing a solution phase) and ATR–FTIR analysis (detecting a polymer phase) describe the dissociation process well, showing similar ranges of dissociation parameters (*α*, p*K*_a_). The overall dissociation trend was well described by comparing four relevant molecular-level physical length scales (*r_c_*, *r_ion_*, *l_B_*, and *r_D_*) in our system.

In the dilute solution (DI water), the enhanced electrostatic interaction (*r_c_* ≲ *l_B_* ≪ *r_D_* < *r_ion_*) is the dominant factor for suppressing dissociation and thus moving p*K*_a_ to a higher range. The influence of varying polymer composition (mIEC and PEGDA cross-linker length, *n*) has a relatively smaller impact on dissociation and p*K*_a_ in the dilute condition. In contrast, in the concentrated salt solution (1 M NaCl (aq)), the screened electrostatic interaction (*r_D_* ≪ *r_c_* ≲ *l_B_* ≈ *r_ion_*) via the added external salts is the governing factor for promoting dissociation and thus substantially lowering p*K*_a_. Because of the screened electrostatic interaction, changing polymer composition (mIEC, *n*) shows greater influence on dissociation and p*K*_a_. If an external salt condition is fixed, varying a polymer composition (mIEC, *n*) in AA–PEGDA series leads to different water swelling (*ϕ_w_*) (high *ϕ_w_* favors more dissociation), altering the dissociation trend and p*K*_a_. In our system, varying external salt conditions and water swelling (*ϕ_w_*) ratios in the polymers are the major molecular parameters for controlling the dissociation behavior and p*K*_a_. Our multiscale analysis of dissociation can help us tune the dissociation behavior of weakly charged polymers in complex environments to achieve desired transport properties.

Compared with the other AA-based polymers in the literature, our AA–PEGDA series follows the general trend of other polymers. Our AA–PEGDA series shows higher p*K*_a_ values in dilute conditions and a lower p*K*_a_ range in concentrated salt conditions. To the best of our knowledge, this is the first time the dissociation process has been systematically studied using a film form with systematically varied polymer composition and external conditions. Our AA–PEGDA system can serve as a useful platform to understand the molecular-level physical picture of the dissociation process of weakly charged polymers. Using this platform, our subsequent paper will further discuss the effect of external salt concentration on the dissociation process using a fixed chemical structure in order to advance our understanding of dissociation. Together, this system can offer a versatile foundation to design next–generation polymer membranes for sustainable technologies.

## Supplementary Material



## Notes

ATR–FTIR analyses, degree of ionization (*α*) vs pH plots using the modified Henderson–Hasselbalch equation, p*K*_a_ and *B* values, four relevant length scales vs polymer composition and external salt solution conditions (PDF)

## References

[1] Kaushika, N. ; Reddy, K. ; Kaushik, K. Sustainable Energy and the Environment: A Clean Technology Approach. Springer, 2016. 10.1007/978-3-319-29446-9

[2] Commission, E. Implementing the Water–Energy–Food–Ecosystems Nexus and achieving the Sustainable Development Goals. UNESCO Publishing, 2021.

[3] Keairns D., Darton R., Irabien A. (2016). The energy-water-food nexus. Annu. Rev. Chem. Biomol. Eng.

[4] National Academies of Sciences and Medicine and Division on Earth and Life Studies and Board on Chemical Sciences and Committee on a Research Agenda for a New Era in Separation Science. A research agenda for transforming separation science. National Academies Press, 2019.

[5] Peinemann, K.-V. ; Nunes, S. P. Membranes for life sciences. John Wiley & Sons, 2011.

[6] Elimelech M., Phillip W. A. (2011). The future of seawater
desalination: Energy, technology,
and the environment. Science.

[7] Mauger A., Julien C. M., Paolella A., Armand M., Zaghib K. (2019). Building better
batteries in the solid state: A review. Materials.

[8] Zhang H., Shen P. K. (2012). Recent development
of polymer electrolyte membranes
for fuel cells. Chem. Rev.

[9] Werber J. R., Osuji C. O., Elimelech M. (2016). Materials
for next-generation desalination
and water purification membranes. Nat. Rev.
Mater.

[10] Zhao W.-Y., Zhou M., Yan B., Sun X., Liu Y., Wang Y., Xu T., Zhang Y. (2018). Waste conversion and
resource recovery from wastewater by ion exchange membranes: State-of-the-art
and perspective. Ind. Eng. Chem. Res.

[11] Freeman, B. D. ; Pinnau, I. Gas and liquid separations using membranes: An overview. In Advanced Materials for Membrane Separations. ACS 2004. 10.1021/bk-2004-0876.ch001

[12] Oh H. J., Aboian M. S., Yi M. Y. J., Maslyn J. A., Loo W. S., Jiang X., Parkinson D. Y., Wilson M. W., Moore T., Yee C. R. (2019). 3d printed absorber for capturing chemotherapy drugs
before they spread through the body. ACS Cent.
Sci.

[13] Stapf H., Selbmann F., Joseph Y., Rahimi P. (2024). Membrane-based nems/mems
biosensors. ACS Appl. Electron. Mater.

[14] Hazarika G., Jadhav S. V., Ingole P. G. (2024). Exploring the potential of polymeric
membranes in cutting-edge chemical and biomedical applications: A
review. Mater. Today Commun.

[15] Siracusa V. (2012). Food packaging
permeability behaviour: A report. Int. J. Polym.
Sci.

[16] Kamcev J., Paul D. R., Freeman B. D. (2017). Effect
of fixed charge group concentration
on equilibrium ion sorption in ion exchange membranes. J. Mater. Chem. A.

[17] Kamcev J., Doherty C. M., Lopez K. P., Hill A. J., Paul D. R., Freeman B. D. (2018). Effect of fixed
charge group concentration on salt
permeability and diffusion coefficients in ion exchange membranes. J. Membr. Sci.

[18] Yan N., Sujanani R., Kamcev J., Galizia M., Jang E.-S., Paul D. R., Freeman B. D. (2022). Influence of fixed charge concentration
and water uptake on ion sorption in amps/pegda membranes. J. Membr. Sci.

[19] Yan N., Sujanani R., Kamcev J., Jang E.-S., Kobayashi K., Paul D. R., Freeman B. D. (2022). Salt and ion transport in a series
of crosslinked amps/pegda hydrogel membranes. J. Membr. Sci.

[20] Jang E.-S., Kamcev J., Kobayashi K., Yan N., Sujanani R., Dilenschneider T. J., Park H. B., Paul D. R., Freeman B. D. (2019). Influence
of water content on alkali metal chloride transport in cross-linked
poly (ethylene glycol) diacrylate. 1. Ion sorption. Polymer.

[21] Jang E.-S., Kamcev J., Kobayashi K., Yan N., Sujanani R., Dilenschneider T. J., Park H. B., Paul D. R., Freeman B. D. (2020). Influence
of water content on alkali metal chloride transport in cross-linked
poly (ethylene glycol) diacrylate. 2. Ion diffusion. Polymer.

[22] Kim Y., Kim T., Kang D. E., Kracaw R. B., Lukaszewski A. J., Szymanski J. S., Rahman C. M., Shaqfeh M. A., Tierney K. M., Doan H. (2024). Weak polyelectrolyte membranes with a wide ion-exchange
capacity (iec) range and limited water swelling in clean technologies
for sustainability. ACS Appl. Polym. Mater.

[23] Müller M., Wirth L., Urban B. (2021). Determination
of the carboxyl dissociation
degree and pka value of mono and polyacid solutions by ftir titration. Macromol. Chem. Phys.

[24] Swift T., Swanson L., Geoghegan M., Rimmer S. (2016). The ph-responsive behaviour
of poly (acrylic acid) in aqueous solution is dependent on molar mass. Soft Matter.

[25] Kodama H., Miyajima T., Mori M., Takahashi M., Nishimura H., Ishiguro S. (1997). A unified analytical
treatment of
the acid-dissociation equilibria of weakly acidic linear polyelectrolytes
and the conjugate acids of weakly basic linear polyelectrolytes. Colloid Polym. Sci.

[26] Miyajima T., Mori M., Ishiguro S.-I. (1997). Analysis of complexation equilibria
of polyacrylic acid by a donnan-based concept. J. Colloid Interface Sci.

[27] Helfferich, F. G. Ion Exchange. Courier Corporation, 1995.

[28] Soldatov V. (1998). Potentiometric
titration of ion exchangers. React. Funct. Polym.

[29] Fisher S., Kunin R. (1956). Effect of cross-linking on the properties
of carboxylic polymers.
I. Apparent dissociation constants of acrylic and methacrylic acid
polymers. J. Phys. Chem.

[30] Gregor H. P., Hamilton M. J., Becher J., Bernstein F. (1955). Studies on
ion exchange resins. Xiv. Titration, capacity and swelling of methaerylic
acid resins. J. Phys. Chem.

[31] Coronell O., Marinas B. J., Cahill D. G. (2011). Depth heterogeneity
of fully aromatic
polyamide active layers in reverse osmosis and nanofiltration membranes. Environ. Sci. Technol.

[32] Kim Y., Kim T., Kang D. E., Szymanski J. S., Kracaw R. B., Lukaszewski A. J., Tierney K. M., Shaqfeh M. A., Rahman C. M., Jeung
Oh H. (2024). Determination of carboxyl dissociation degree and p k a in weak polyelectrolyte
membranes via pot titration and ftir analysis for clean technologies
in sustainability. Macromolecules.

[33] Al-Amshawee S., Yunus M. Y. B. M., Azoddein A. A. M., Hassell D. G., Dakhil I. H., Hasan H. A. (2020). Electrodialysis desalination for water and wastewater:
A review. Chem. Eng. J.

[34] Zhang H., Lu W., Li X. (2019). Progress and perspectives
of flow battery technologies. Electrochem. Energy
Rev.

[35] Millero F. J., Feistel R., Wright D. G., McDougall T. J. (2008). The composition
of standard seawater and the definition of the reference-composition
salinity scale. Deep Sea Res., Part I.

[36] Flory, P. J. Principles of polymer chemistry. Cornell University Press, 1953.

[37] Lin H., Kai T., Freeman B. D., Kalakkunnath S., Kalika D. S. (2005). The effect of cross-linking
on gas permeability in cross-linked poly (ethylene glycol diacrylate). Macromolecules.

[38] Kalakkunnath S., Kalika D. S., Lin H., Raharjo R. D., Freeman B. D. (2007). Molecular
relaxation in cross-linked poly (ethylene glycol) and poly (propylene
glycol) diacrylate networks by dielectric spectroscopy. Polymer.

[39] Yan N., Paul D. R., Freeman B. D. (2018). Water and ion sorption in a series
of cross-linked amps/pegda hydrogel membranes. Polymer.

[40] Stumm, W. ; Morgan, J. J. Aquatic chemistry: Chemical equilibria and rates in natural waters. John Wiley & Sons, 2013.

[41] Riché E., Carrié A., Andin N., Mabic S. (2006). High-purity
water and
ph. Am. Lab.

[42] Lin H., Freeman B. D. (2004). Gas solubility, diffusivity and permeability in poly
(ethylene oxide). J. Membr. Sci.

[43] Walsh, D. ; Zoller, P. Standard pressure volume temperature data for polymers. CRC press, 1995.

[44] Brandrup, J. ; Immergut, E. H. ; Grulke, E. A. ; Abe, A. ; Bloch, D. R. Polymer handbook. Wiley New York, 1999.

[45] Oh H. J., McGrath J. E., Paul D. R. (2018). Water and
salt transport properties
of disulfonated poly (arylene ether sulfone) desalination membranes
formed by solvent-free melt extrusion. J. Membr.
Sci.

[46] Geise G. M., Paul D. R., Freeman B. D. (2014). Fundamental water and salt transport
properties of polymeric materials. Prog. Polym.
Sci.

[47] Xie W., Cook J., Park H. B., Freeman B. D., Lee C. H., McGrath J. E. (2011). Fundamental salt
and water transport properties in
directly copolymerized disulfonated poly (arylene ether sulfone) random
copolymers. Polymer.

[48] Geise G. M., Falcon L. P., Freeman B. D., Paul D. R. (2013). Sodium chloride
sorption in sulfonated polymers for membrane applications. J. Membr. Sci.

[49] Silverstein, R. M. ; Bassler, G. C. Spectrometric identification of organic compounds. Wiley, 1962.

[50] Swinehart D. F. (1962). The beer-lambert
law. J. Chem. Educ.

[51] Zimudzi T. J., Feldman K. E., Sturnfield J. F., Roy A., Hickner M. A., Stafford C. M. (2018). Quantifying carboxylic acid concentration
in model
polyamide desalination membranes via fourier transform infrared spectroscopy. Macromolecules.

[52] Kamcev J., Paul D. R., Freeman B. D. (2015). Ion activity
coefficients in ion
exchange polymers: Applicability of manning’s counterion condensation
theory. Macromolecules.

[53] Kitto D., Kamcev J. (2022). Manning condensation
in ion exchange membranes: A review
on ion partitioning and diffusion models. J.
Polym. Sci.

[54] Muthukumar M. (2017). 50th anniversary
perspective: A perspective on polyelectrolyte solutions. Macromolecules.

[55] Bjerrum, N. Untersuchungen über ionenassoziation. AF Høst, 1926.

[56] Manning G.
S. (1969). Limiting
laws and counterion condensation in polyelectrolyte solutions i. Colligative
properties. J. Chem. Phys.

[57] Noda, N. ; Obrzut, J. High frequency dielectric relaxation in polymers filled with ferroelectric ceramics. In Materials Research Society Symposium Proceedings. Materials Research Society, Warrendale, Pa., 2002 Vol. 698, pp 113–120.

[58] Barthel, J. ; Krienke, H. ; Kunz, W. ; Kunz, W. Physical chemistry of electrolyte solutions: Modern aspects. Springer Science & Business Media, 1998.

[59] Debye V. P. (1923). Zur theorie
der electrolyte. Phys. Z..

[60] Kamcev J., Jang E.-S., Yan N., Paul D. R., Freeman B. D. (2015). Effect
of ambient carbon dioxide on salt permeability and sorption measurements
in ion-exchange membranes. J. Membr. Sci.

[61] Hale D., Reichenberg D. (1949). Equilibrium
and rate studies of cation-exchange with
monofunctional resins. Discuss. Faraday Soc.

[62] Katchalsky A., Lifson S., Mazur J. (1953). The electrostatic
free energy of
polyelectrolyte solutions. I. Randomly kinked macromolecules. J. Polym. Sci.

[63] Lifson S., Katchalsky A. (1954). The electrostatic
free energy of polyelectrolyte solutions.
Ii. Fully stretched macromolecules. J. Polym.
Sci.

[64] Michaeli I., Katchalsky A. (1957). Potentiometric titration of polyelectrolyte
gels. J. Polym. Sci.

[65] Katchalsky A., Spitnik P. (1947). Potentiometric titrations
of polymethacrylic acid. J. Polym. Sci.

[66] Strobel H., Gable R. (1954). Titration studies as a means of characterizing anion-exchange resins. J. Am. Chem. Soc.

[67] Kunin R., Fisher S. (1962). Effect of cross-linking
on the properties of carboxylic
polymers. Ii. Apparent dissociation constants as a function of the
exchanging monovalent cation. J. Phys. Chem.

[68] Soldatov V. S. (1995). Quantitative
presentation of potentiometric titration curves of ion exchangers. Ind. Eng. Chem. Res.

[69] Canal T., Peppas N. A. (1989). Correlation between
mesh size and equilibrium degree
of swelling of polymeric networks. J. Biomed.
Mater. Res.

[70] Gustafson R. L. (1964). Hydrogen
ion equilibria in cross-linked polymethacrylic acid—sodium
chloride systems. J. Phys. Chem.

[71] Balzer C., Wang Z.-G. (2023). Electroresponse of weak polyelectrolyte brushes. Eur. Phys. J. E.

[72] Ferrand-Drake
del Castillo G., Hailes R. L. N., Dahlin A. (2020). Large changes in protonation
of weak polyelectrolyte brushes with salt concentration—implications
for protein immobilization. J. Phys. Chem. Lett.

[73] Kumar N. A., Seidel C. (2005). Polyelectrolyte brushes with added salt. Macromolecules.

[74] Arnold R. (1957). The titration
of polymeric acids. J. Colloid Sci.

[75] Longo G. S., Olvera de la Cruz M., Szleifer I. (2011). Molecular theory of weak polyelectrolyte
gels: The role of ph and salt concentration. Macromolecules.

