# Supplementary material for: Carboxyl Dissociation Degree (α) and pK a of Weak Polyelectrolyte Membranes in Dilute and Concentrated External Salt Solutions for Sustainable Technologies
Source: Macromolecules. 2026 May 4;59(10):5784–804. doi: 10.1021/acs.macromol.5c02531 (PMC13217623; doi:10.1021/acs.macromol.5c02531)
Supplement: Supplementary file 1 [file ma5c02531_si_001.pdf]

Supplementary Information (SI)

Carboxyl Dissociation Degree (α) and pK_a of Weak Polyelectrolyte Membranes in Dilute and Concentrated External Salt Solutions for Sustainable Technologies

Yongha Kim[1], Michael A. Shaqfeh[1], Charleen M. Rahman[1], Riley B. Kracaw[1], Steven D. Marotta[2], Nikitha S. Kanumuru[3], Ania Chandra[1], Andrew J. Lukaszewski[1], Lauren Collins[4] and Hee Jeung Oh[1,3,5,6,*]

[1]Department of Chemical Engineering, The Pennsylvania State University, University Park, PA 16802, USA
[2]Department of Chemistry, The Pennsylvania State University, University Park, Pennsylvania 16802, USA
[3]Department of Materials Science and Engineering, The Pennsylvania State University, University Park, Pennsylvania 16802, USA
[4]Department of Biomedical Engineering, The University of Michigan, Ann Harbor, Michigan 48104, USA
[5]Institute of Energy and Environment (IEE), The Pennsylvania State University, University Park, Pennsylvania 16802, USA
[6]Advanced Manufacturing and Design, The Pennsylvania State University, University Park, Pennsylvania 16802, United States

Corresponding Author: Prof. Hee Jeung Oh
Department of Chemical Engineering
Department of Materials Science and Engineering (by courtesy)
Institute of Energy and Environment (IEE)
Advanced Manufacturing and Design (AMD)
The Pennsylvania State University
Email: hjoh@psu.edu
Phone: 814-863-9085

Table S1. Polymer composition of AA–PEGDA network series[1]

Sample [n – mIEC]	Mass of AA monomer [g]	Mass of PEGDA crosslinker [g]	Mass of DMPA [g]	Mass of water [g]	AA content* [wt%]
10 – 0	0.000	15.000	0.015	0.075	0.00
10 – 1	1.081	13.919	0.015	0.075	7.21
10 – 2	2.162	12.838	0.015	0.075	14.41
10 – 3	3.243	11.757	0.015	0.075	21.62
10 – 4	4.324	10.676	0.015	0.075	28.82
13 – 0	0.000	15.000	0.015	0.075	0.00
13 – 1	1.081	13.919	0.015	0.075	7.21
13 – 2	2.162	12.838	0.015	0.075	14.41
13 – 3	3.243	11.757	0.015	0.075	21.62
13 – 4	4.324	10.676	0.015	0.075	28.82

* AA content [wt%] = mass of AA [g] / (mass of AA [g] + mass of PEGDA [g]) × 100

Table S2. Added mmol equivalent NaOH per grams of a dry polymer (x_{NaOH} [mequiv/g]) with respect to the maximum ion–exchange capacity (mIEC) in a polymer coupon [mequiv/g] for pH titration[1, 2]

		NaOH amounts (x_{NaOH}) [mequiv/g]										
mIEC [mequiv/g]	0	0.1	0.3	0.5	0.7	0.9	1.0	1.2	1.5	1.7	2.0	-
	1	0.1	0.3	0.5	0.7	0.9	1.0	1.2	1.5	1.7	2.0	-
	2	0.1	0.5	0.7	1.0	1.2	1.5	2.0	2.2	2.5	3.0	-
	3	0.1	0.5	1.0	1.5	2.0	2.5	3.0	3.2	3.5	4.0	-
	4	0.1	0.5	1.0	1.5	2.0	2.5	3.0	3.5	4.0	4.5	5.0

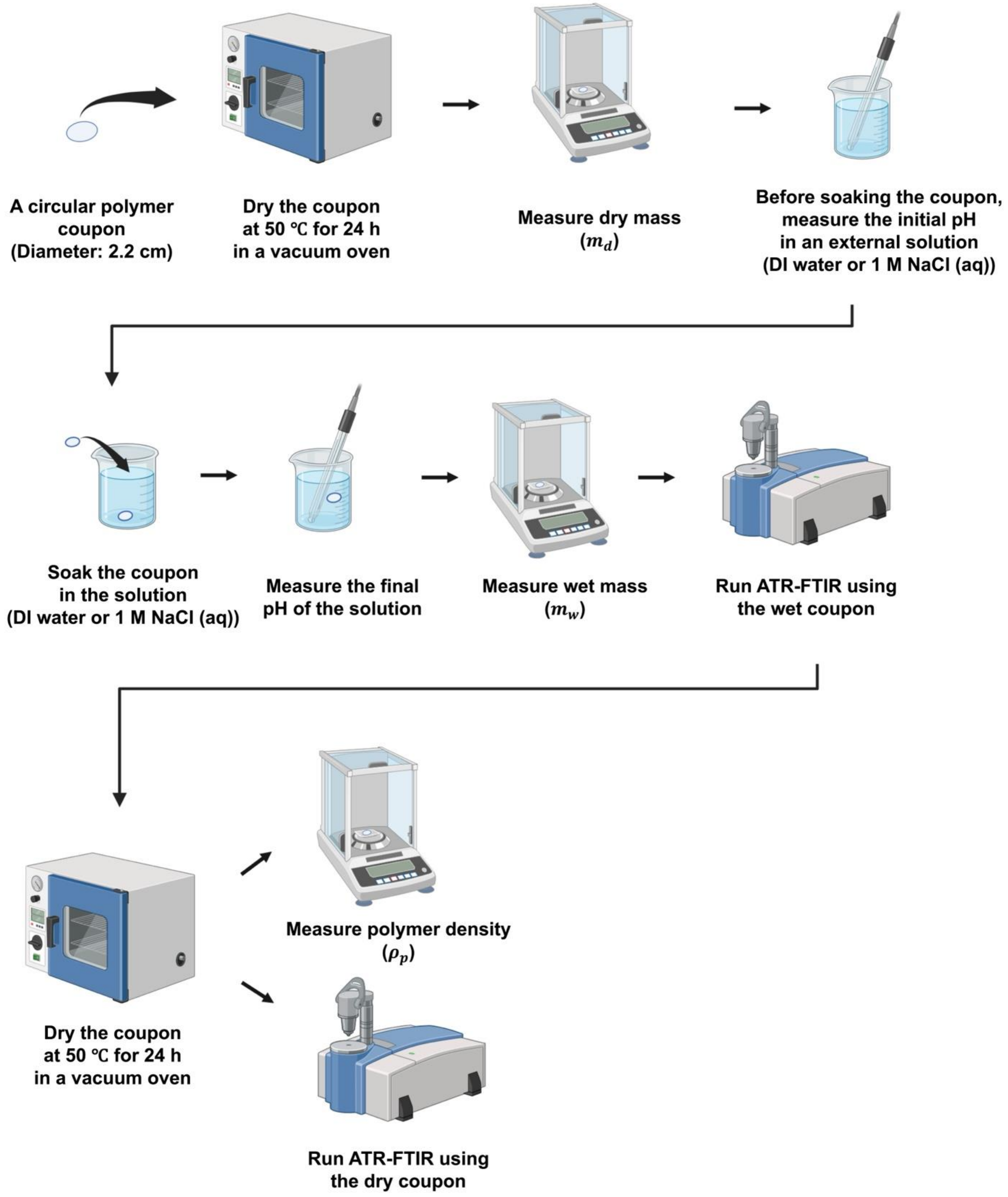

Figure S1. Pre-titration procedure in this study.

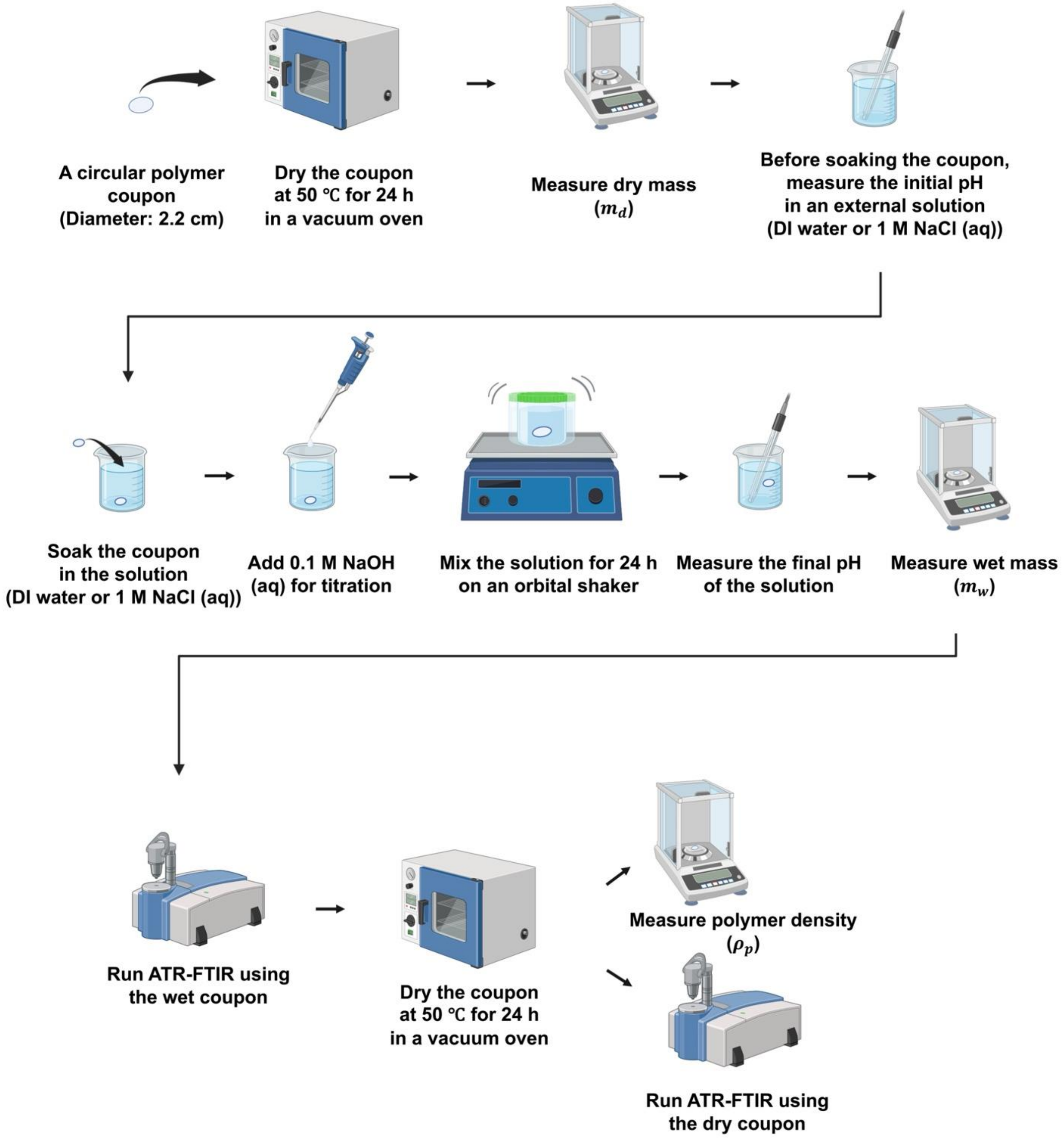

Figure S2. pH titration procedure in this study.

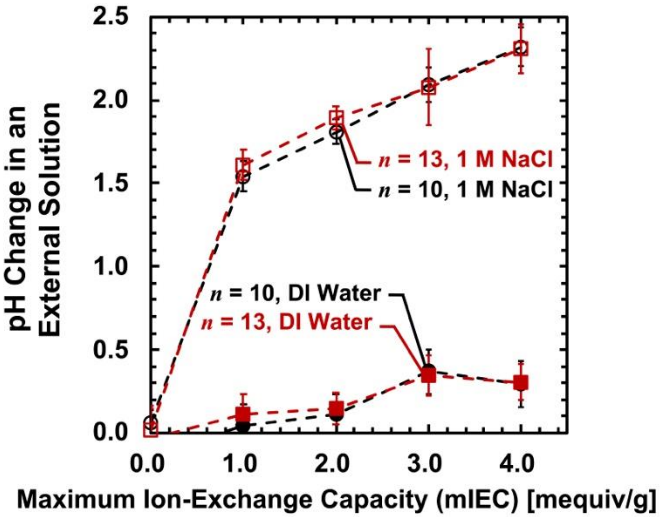

Figure S3. pH change in an external solution after soaking AA–PEGDA film in DI water or 1 M NaCl (aq) solution for 2 days before titration. No strong base (NaOH) was added. Dashed lines are used to guide the eyes. Error bars are included. Error bars represent the standard deviation of at least three independent replicate samples.

S.1. ATR–FTIR before titration

Before titration (without adding NaOH), chemical structure and degree of ionization (α_{IR}) of AA–PEGDA series in 1 M NaCl (aq) solution were characterized via ATR–FTIR analysis using dry and wet polymer films as shown in **Figures S4-S5**. The detailed peak assignment and calculation methods have been discussed in our previous reports and elsewhere[1-7]. To quantify the amounts of dissociable charged groups (COOH) and dissociated charged groups (COO^-) in a polymer, absorbance intensities at 1575 cm^{-1} and 1400 cm^{-1} (from dissociated COO^- groups) are plotted against the maximum ion–exchange capacity (mIEC) of the polymer in **Figure S6a-b**. The areas under the curve at 1575 cm^{-1} (A_{COO^-} from dissociated COO^- groups) and the deconvoluted areas at 1700 cm^{-1} (A_{COOH}, from dissociable COOH groups) are also plotted vs. mIEC in **Figures 2b** and **S7.**

In addition, as mIEC increases (AA monomer content increases), PEGDA cross–linker content decreases in the polymers. Consequently, the absorbance intensities at 1100 cm^{-1} (ether C – O – C stretching from ethylene oxide (EO) groups in PEGDA cross–linker) decrease accordingly as shown in **Figure S6c**.

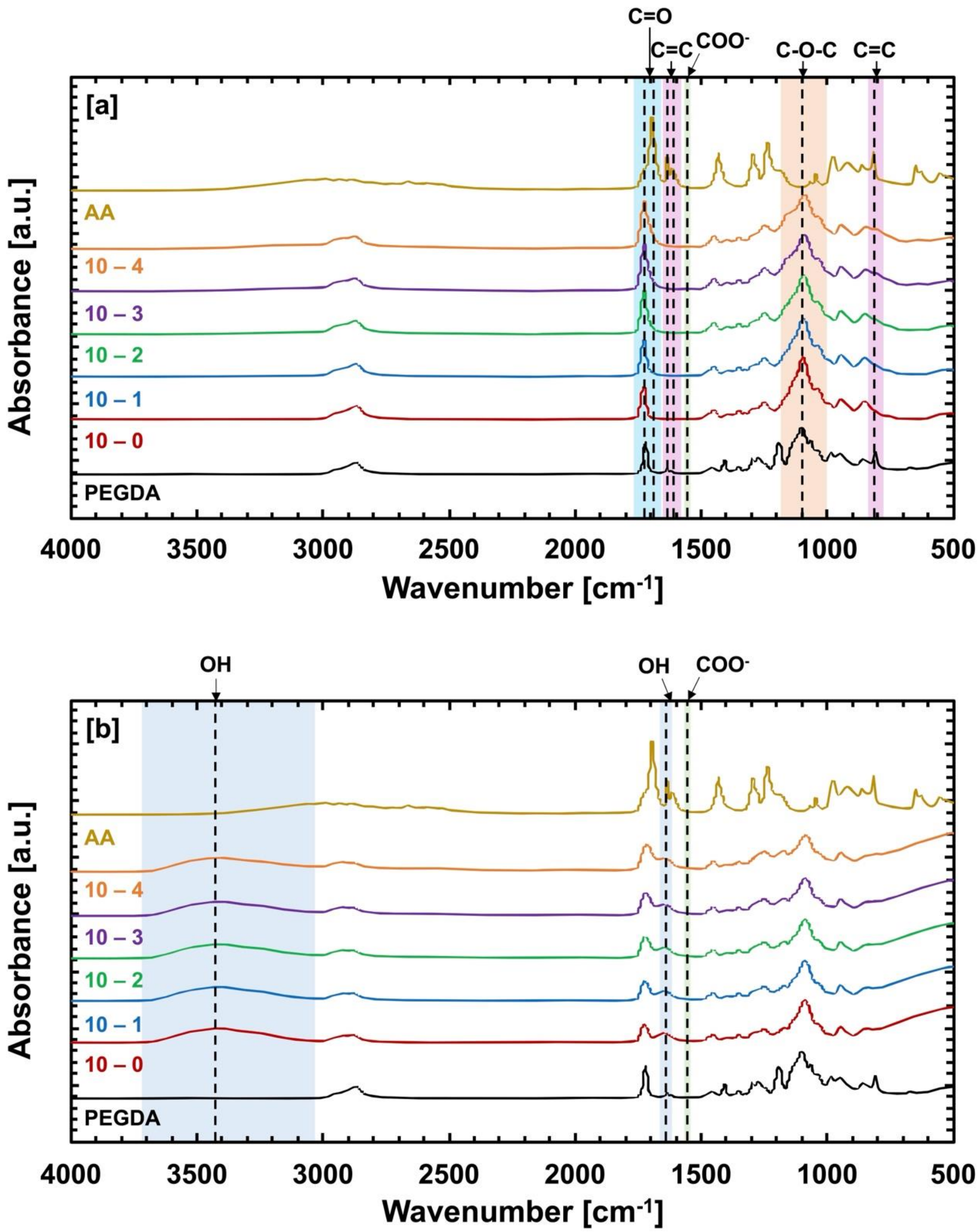

Figure S4. ATR–FTIR spectra of **[a]** dry and **[b]** wet states of AA–PEGDA series with mIEC = 0 – 4 mequiv/g and PEGDA cross–linker length, *n* = 10 before titration. The films were soaked in 1 M NaCl (aq) solution for 2 days before ATR–FTIR analysis. Spectra of AA monomer and PEGDA cross–linker (*n* = 10) are shown for comparison. The spectra are vertically moved for easier viewing.

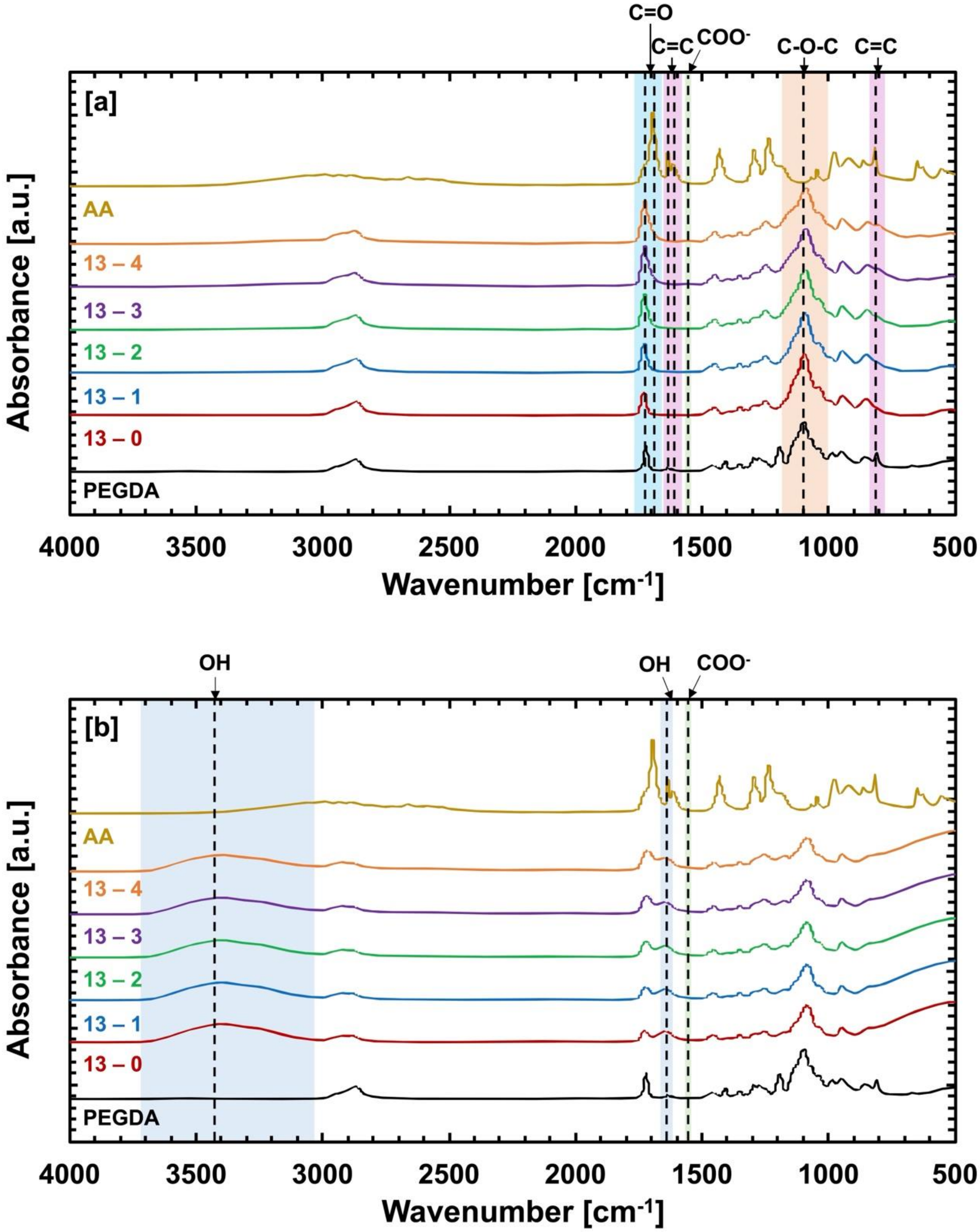

Figure S5. ATR–FTIR spectra of **[a]** dry and **[b]** wet states of AA–PEGDA series with mIEC = 0 – 4 mequiv/g and PEGDA cross–linker length, *n* = 13 before titration. The films were soaked in 1 M NaCl (aq) solution for 2 days before ATR–FTIR analysis. Spectra of AA monomer and PEGDA cross–linker (*n* = 13) are shown for comparison. The spectra are vertically moved for easier viewing.

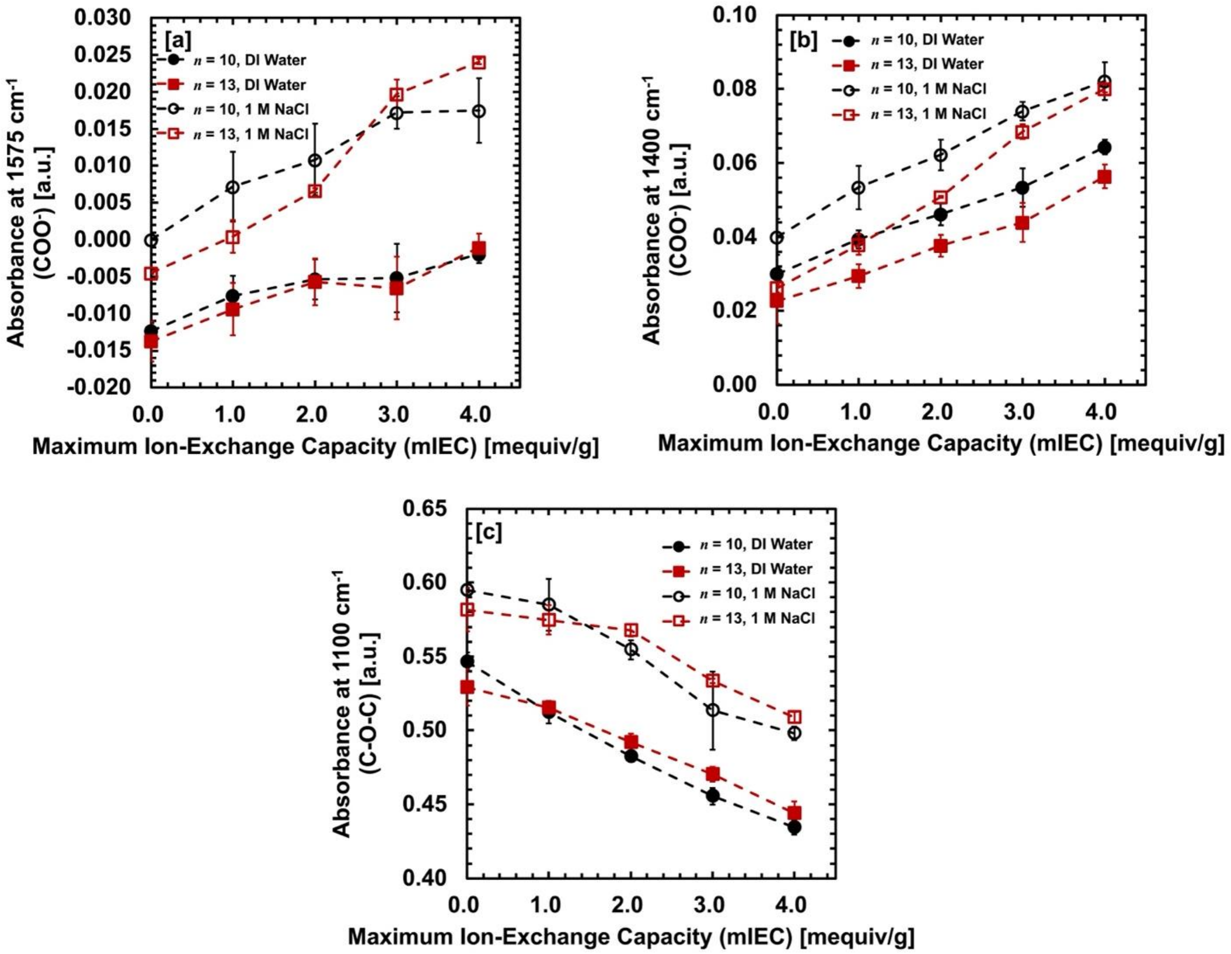

Figure S6. Absorbance intensities at **[a]** 1575 cm^{-1} (from dissociated COO^- groups), **[b]** 1400 cm^{-1} (from dissociated COO^- groups), and **[c]** 1100 cm^{-1} (ether C – O – C stretching from ethylene oxide (EO) groups in PEGDA cross–linker) as a function of maximum ion–exchange capacity (mIEC) [mequiv/g] of dry AA–PEGDA series before titration. The films were soaked in DI water or 1 M NaCl (aq) solution for 2 days and then dried before ATR–FTIR analysis. Dashed lines are used to guide the eyes. Error bars are included.

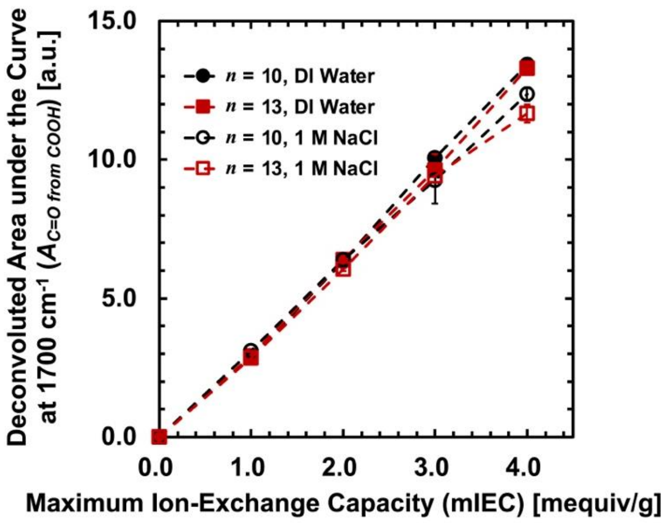

Figure S7. Deconvoluted area under the curve at 1700 cm^{-1} ($A_{C=O\ from\ COOH}$ or A_{COOH}) from dissociable COOH groups in acrylic acid (AA) as a function of the maximum ion–exchange capacity (mIEC) [mequiv/g] of dry AA–PEGDA series before titration. The films were soaked in DI water or 1 M NaCl (aq) solution for 2 days and then dried before ATR–FTIR analysis. Dashed lines are used to guide the eyes. Error bars are included.

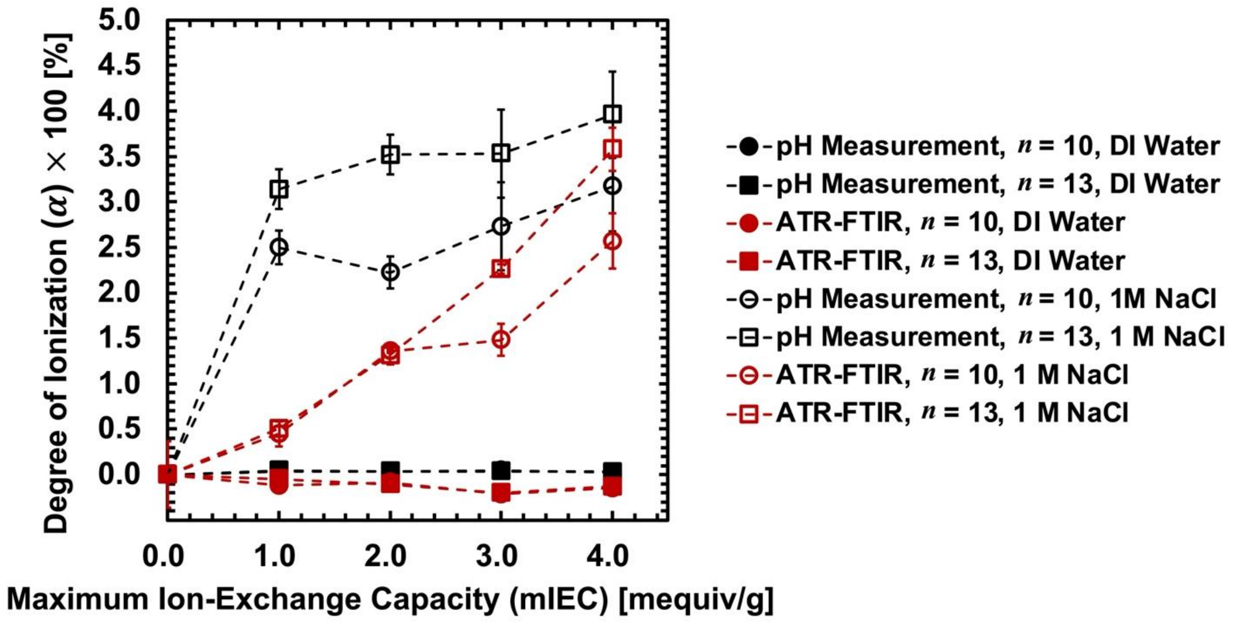

Figure S8. Pre–titration, degree of ionization (α) × 100 [%] of AA–PEGDA series via solution pH measurement and ATR–FTIR analysis. No strong base (NaOH) was added. Dashed lines are used to guide the eyes. Error bars are added.

S.2. ATR–FTIR after titration

After pH titration, chemical structure and degree of ionization (α_{IR}) of AA–PEGDA series in 1 M NaCl (aq) solution were characterized via ATR–FTIR analysis using dry and wet polymer films as shown in **Figures S9-S12**. The detailed peak assignment and calculation methods have been reported in our previous papers[1, 2]. In cross–linked PEGDA networks (10 – 0 and 13 – 0) without any dissociable COOH groups (i.e., no AA monomer), as the external pH increases, the absorbance intensities at 1575 cm^{-1} and 1400 cm^{-1} (from dissociated COO^- groups) are not observed since no dissociable COOH group presents (see **Figure S13a-d**).

In contrast, in AA–PEGDA series with dissociable COOH groups (mIEC = 1 – 4 mequiv/g), as the external pH increases, the absorbance intensities at 1575 cm^{-1} and 1400 cm^{-1} (from dissociated COO^- groups) increase as shown in **Figure S13a-d**. The areas under the curve at 1575 cm^{-1} (from dissociated COO^- groups in AA) increase (see **Figure S14a-b**) while the deconvoluted areas under the curve at 1700 cm^{-1} (from dissociable COOH groups in AA) decrease as expected (see **Figure S14c-d**). In addition, as the external pH increases, the absorbance intensities at 1100 cm^{-1} (ether C – O – C stretching from ethylene oxide (EO) groups in PEGDA cross–linker) remain unchanged (see **Figure 13e-f**). This confirms no chemical degradation occurred during titration.

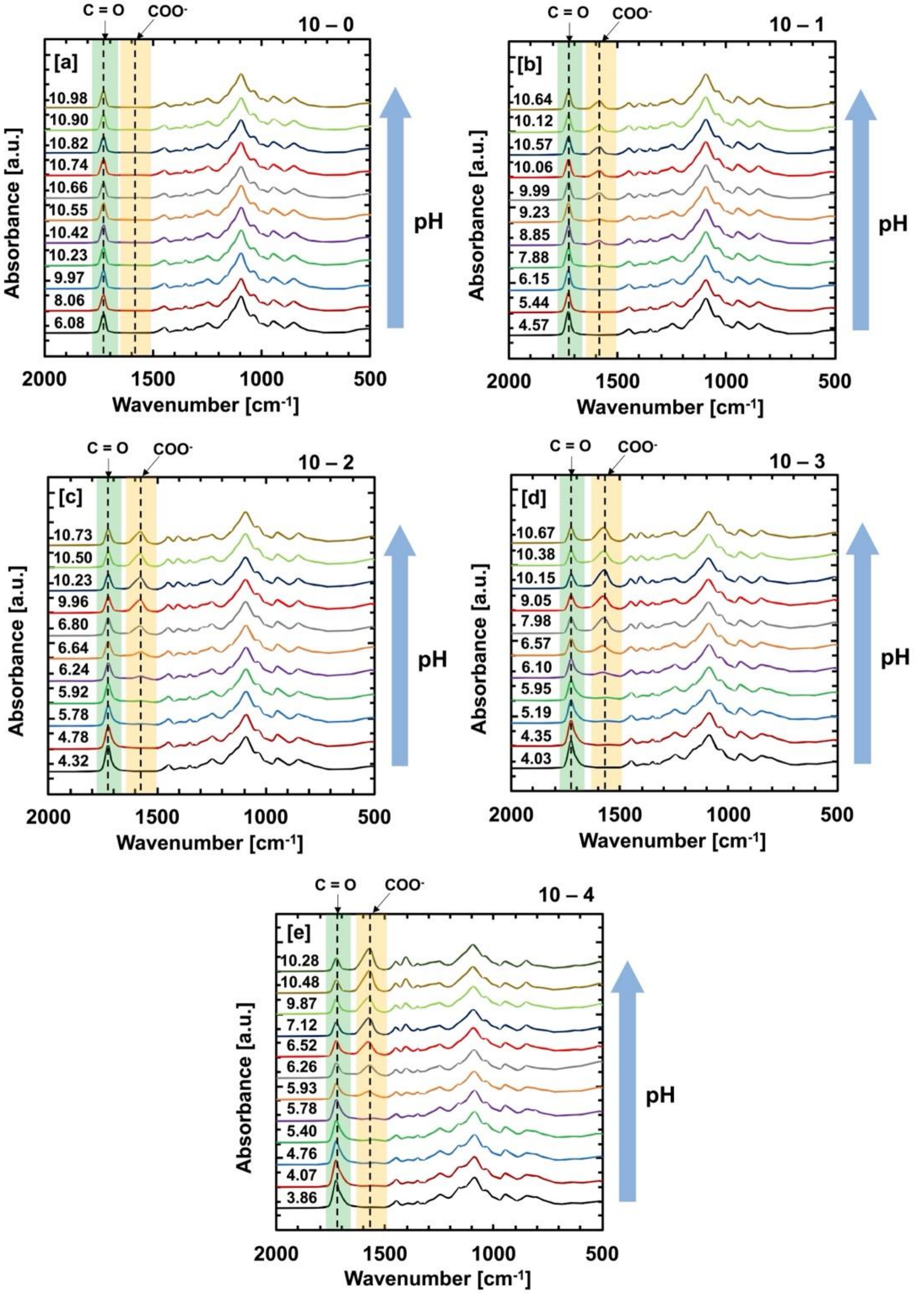

Figure S9. **[a]** ATR–FTIR spectra of control, dry cross–linked PEGDA network (n = 10), i.e., 10 – 0 formulation vs. pH. ATR–FTIR spectra of dry AA–PEGDA series (n = 10) with **[b]** mIEC = 1 mequiv/g, **[c]** mIEC = 2 mequiv/g, **[d]** mIEC = 3 mequiv/g, and **[e]** mIEC = 4 mequiv/g vs. pH in 1 M NaCl (aq) solution. The spectra are vertically moved for easier comparison. The dashed lines are used to guide the eyes.

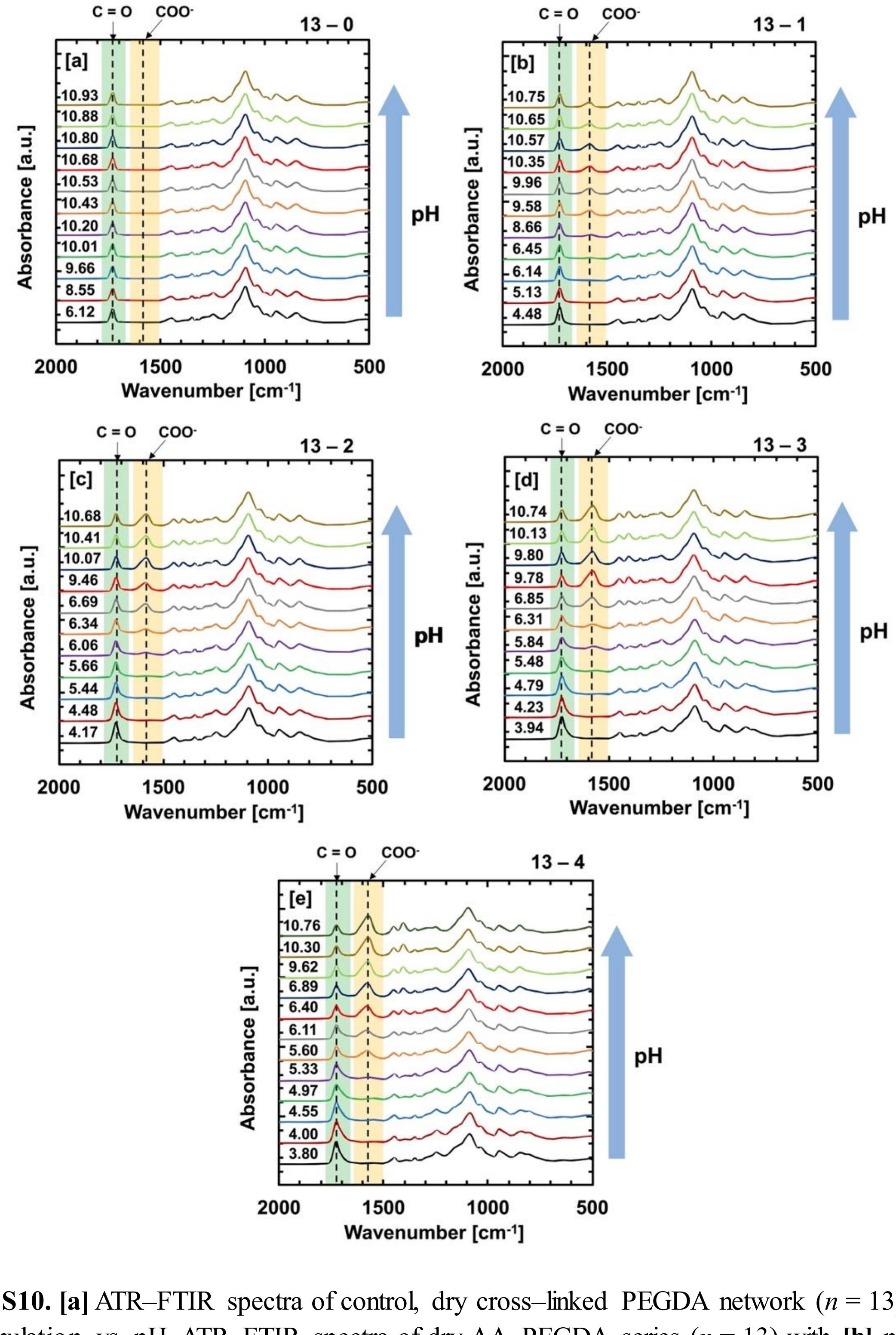

Figure S10. **[a]** ATR–FTIR spectra of control, dry cross–linked PEGDA network (n = 13), i.e., 13 – 0 formulation vs. pH. ATR–FTIR spectra of dry AA–PEGDA series (n = 13) with **[b]** mIEC = 1 mequiv/g, **[c]** mIEC = 2 mequiv/g, **[d]** mIEC = 3 mequiv/g, and **[e]** mIEC = 4 mequiv/g vs. pH in 1 M NaCl (aq) solution. The spectra are vertically moved for easier comparison. The dashed lines are used to guide the eyes.

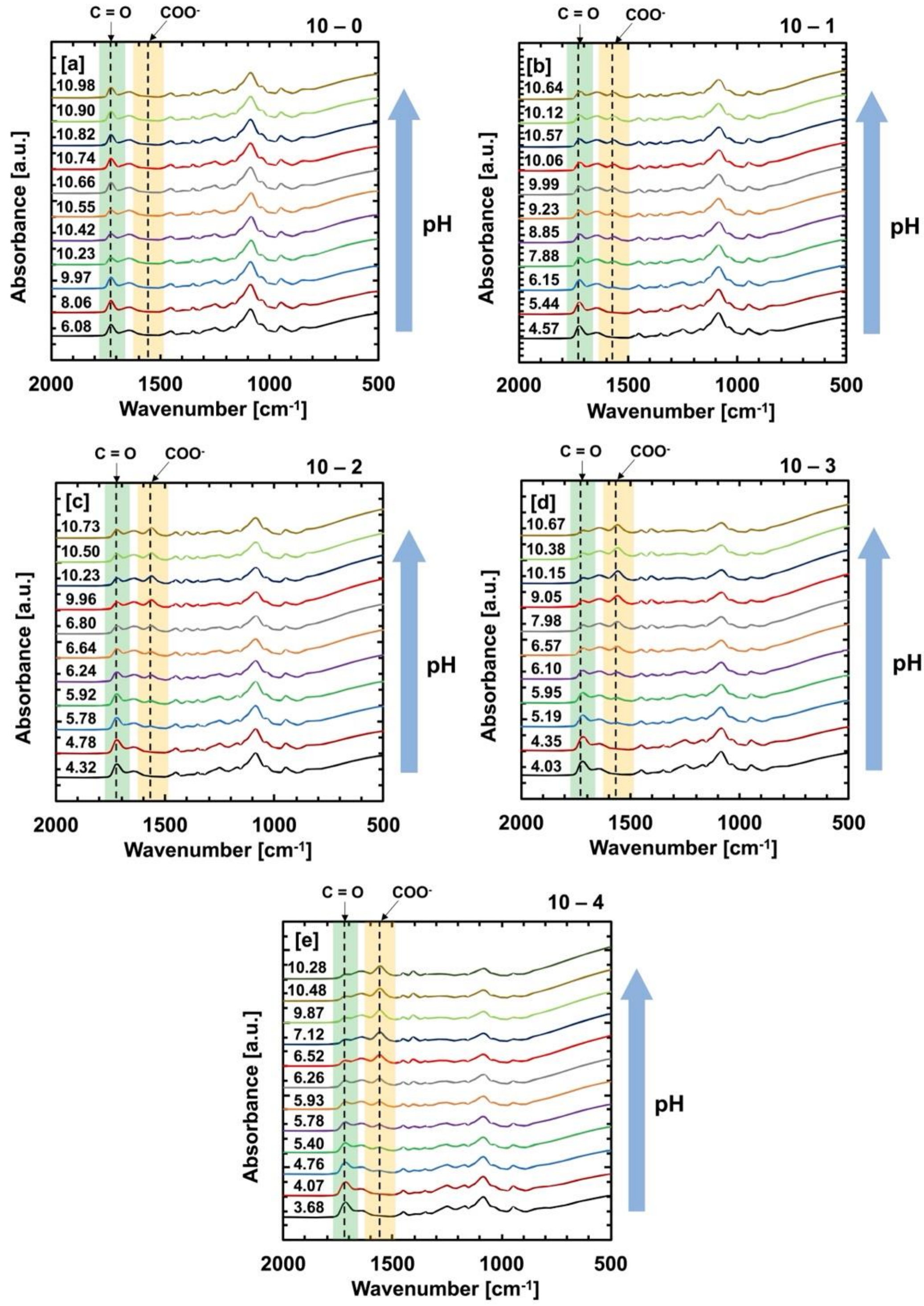

Figure S11. [a] ATR–FTIR spectra of control, wet cross–linked PEGDA network (*n* = 10), i.e., 10 – 0 formulation vs. pH. ATR–FTIR spectra of wet AA–PEGDA series (*n* = 10) with **[b]** mIEC = 1 mequiv/g, **[c]** mIEC = 2 mequiv/g, **[d]** mIEC = 3 mequiv/g, and **[e]** mIEC = 4 mequiv/g vs. pH in 1 M NaCl (aq) solution. The spectra are vertically moved for easier comparison. The dashed lines are used to guide the eyes.

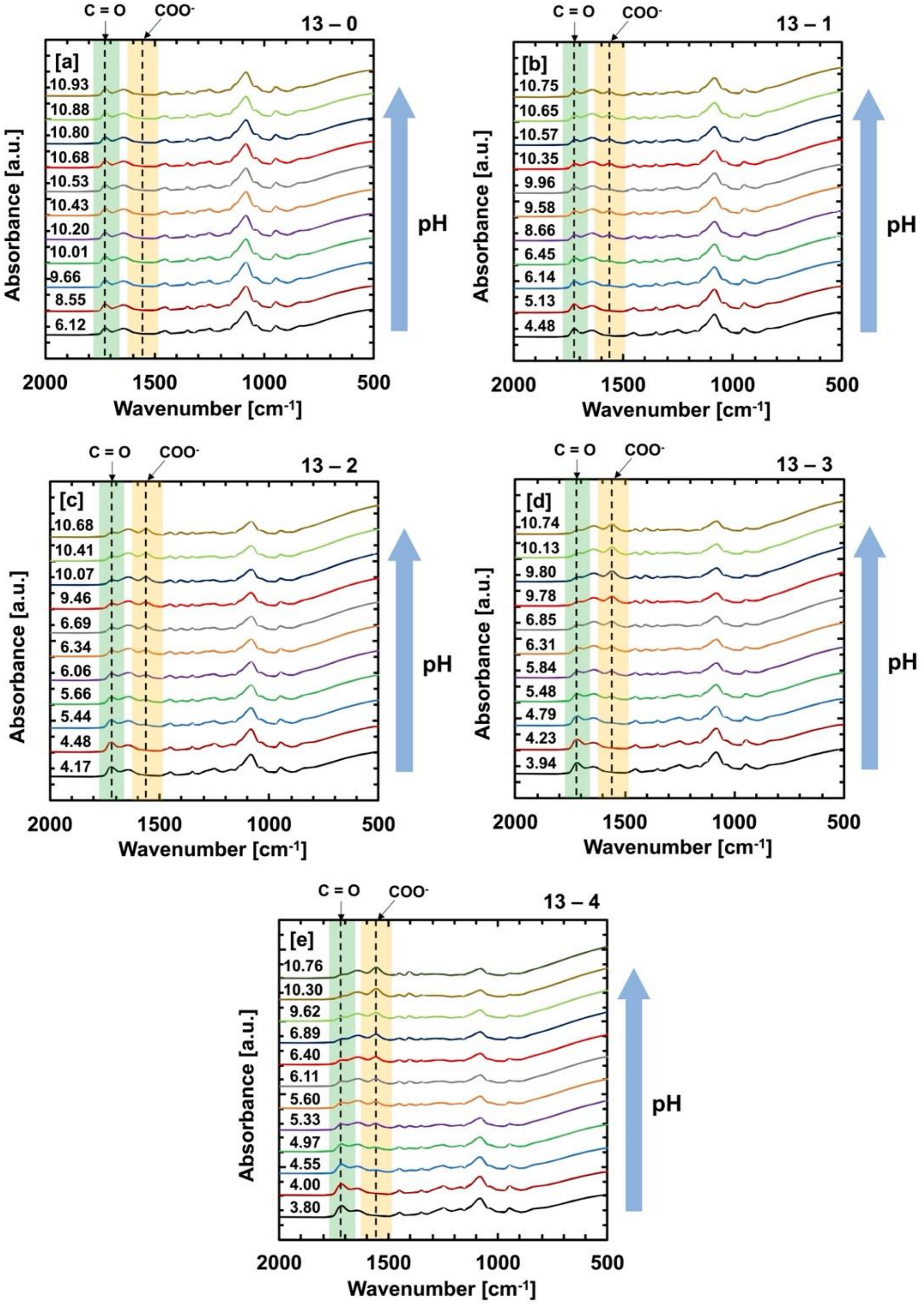

Figure S12. [a] ATR–FTIR spectra of control, wet cross–linked PEGDA network (n = 13), i.e., 13 – 0 formulation vs. pH. ATR–FTIR spectra of wet AA–PEGDA series (n = 13) with **[b]** mIEC = 1 mequiv/g, **[c]** mIEC = 2 mequiv/g, **[d]** mIEC = 3 mequiv/g, and **[e]** mIEC = 4 mequiv/g vs. pH in 1 M NaCl (aq) solution. The spectra are vertically moved for easier comparison. The dashed lines are used to guide the eyes.

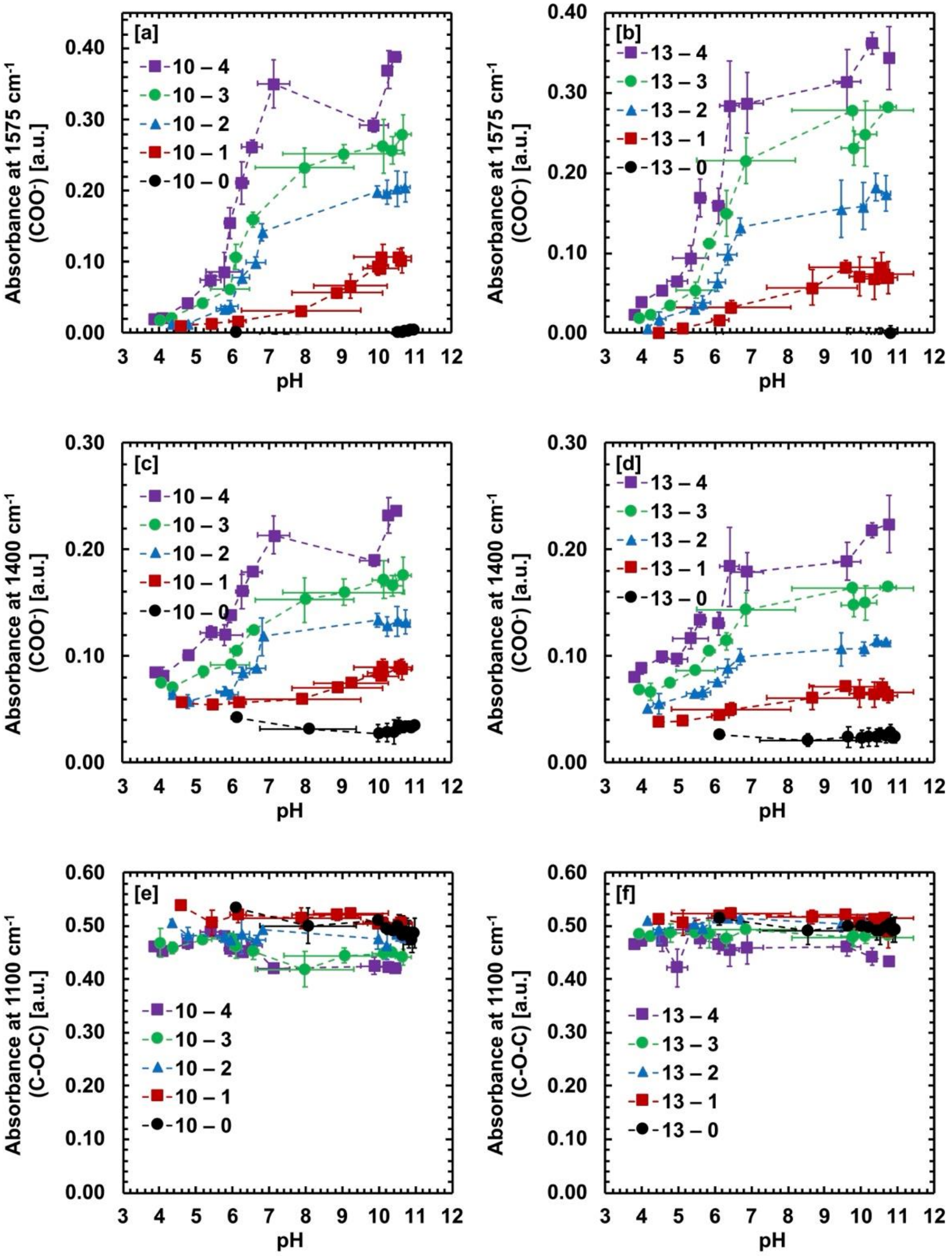

Figure S13. Absorbance intensities at **[a, b]** 1575 cm^{-1} (from dissociated COO^- groups), **[c, d]** 1400 cm^{-1} (from dissociated COO^- groups), and **[e, f]** 1100 cm^{-1} (ether C – O – C stretching from ethylene oxide (EO) groups in PEGDA cross–linker) of AA–PEGDA series vs. pH in 1 M NaCl (aq) solution. **[a, c, e]** are from $n = 10$ series. **[b, d, f]** are from $n = 13$ series. The dashed lines are used to guide the eyes. Error bars are included.

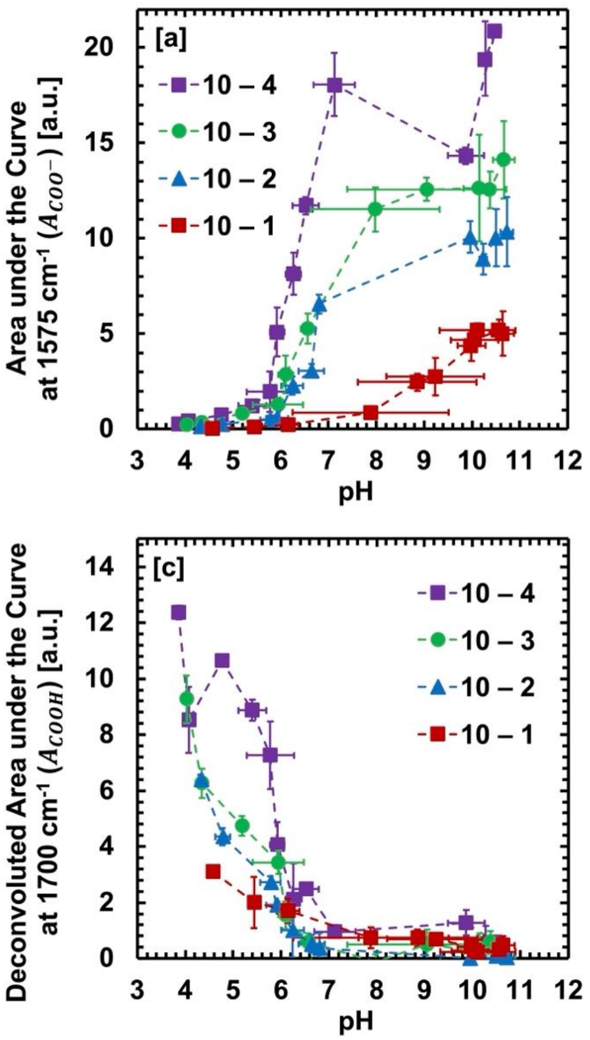

Figure S14. Areas under the curve at **[a, b]** 1575 cm^{-1} from dissociated COO^- groups (A_{COO^-}) in AA–PEGDA series vs. pH in 1 M NaCl (aq) solution. **[c, d]** Deconvoluted areas under the curve at 1700 cm^{-1} from dissociable COOH groups ($A_{C=O\ from\ COOH}$ or A_{COOH}) vs. pH in 1 M NaCl (aq) solution. **[a, c]** are from n = 10 series. **[b, d]** are from n = 13 series. The dashed lines are used to guide the eyes. Error bars are included.

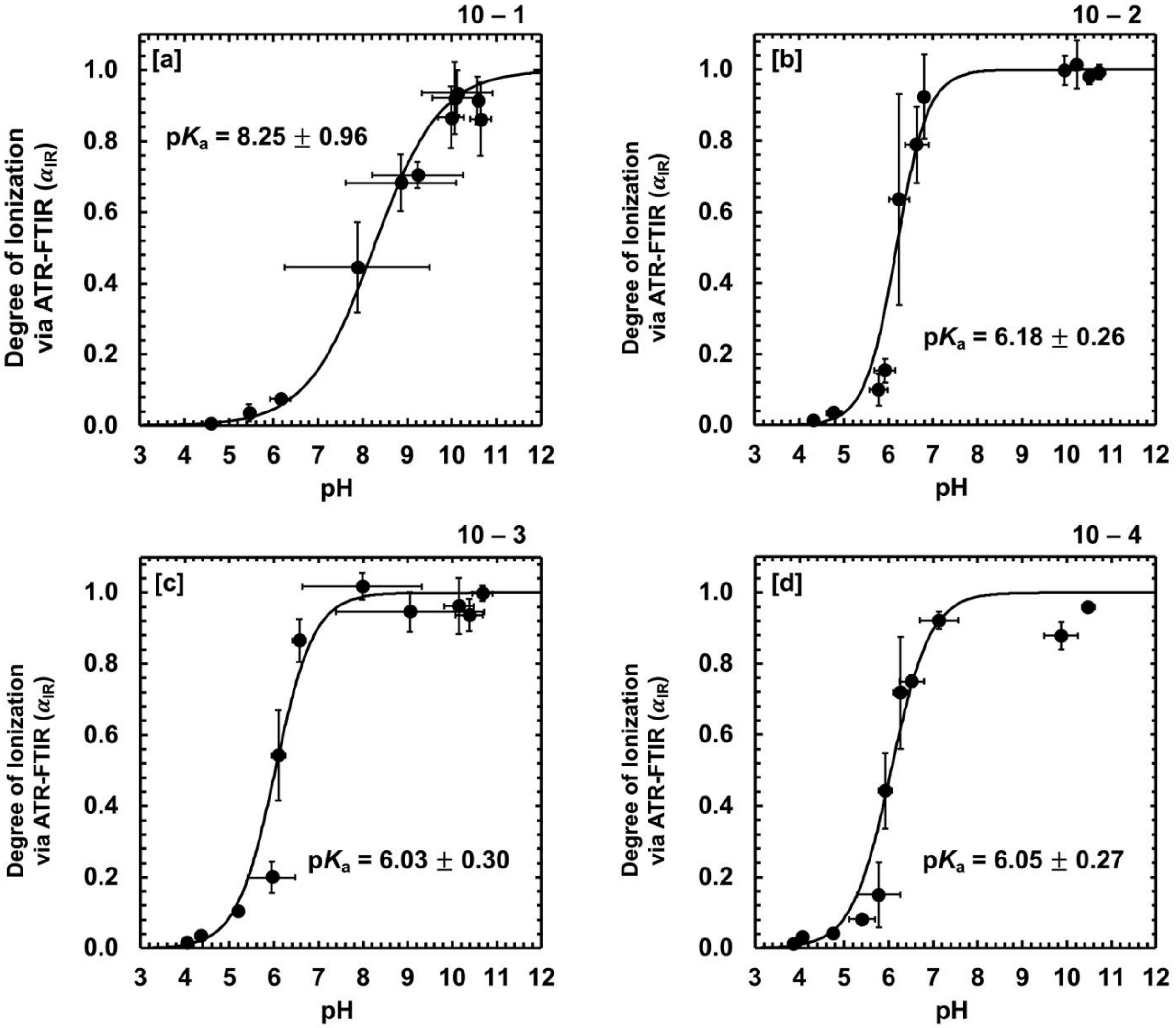

Figure S15. Degree of ionization (α_{IR}) vs. pH of **[a]** 10 – 1, **[b]** 10 – 2, **[c]** 10 – 3, and **[d]** 10 – 4 AA–PEGDA series (mIEC: 1 – 4 mequiv/g, n = 10 series) in 1 M NaCl (aq) solution via ATR–FTIR analysis. Solid lines are the best fitting using the modified Henderson–Hasselbalch equation. Error bars are included.

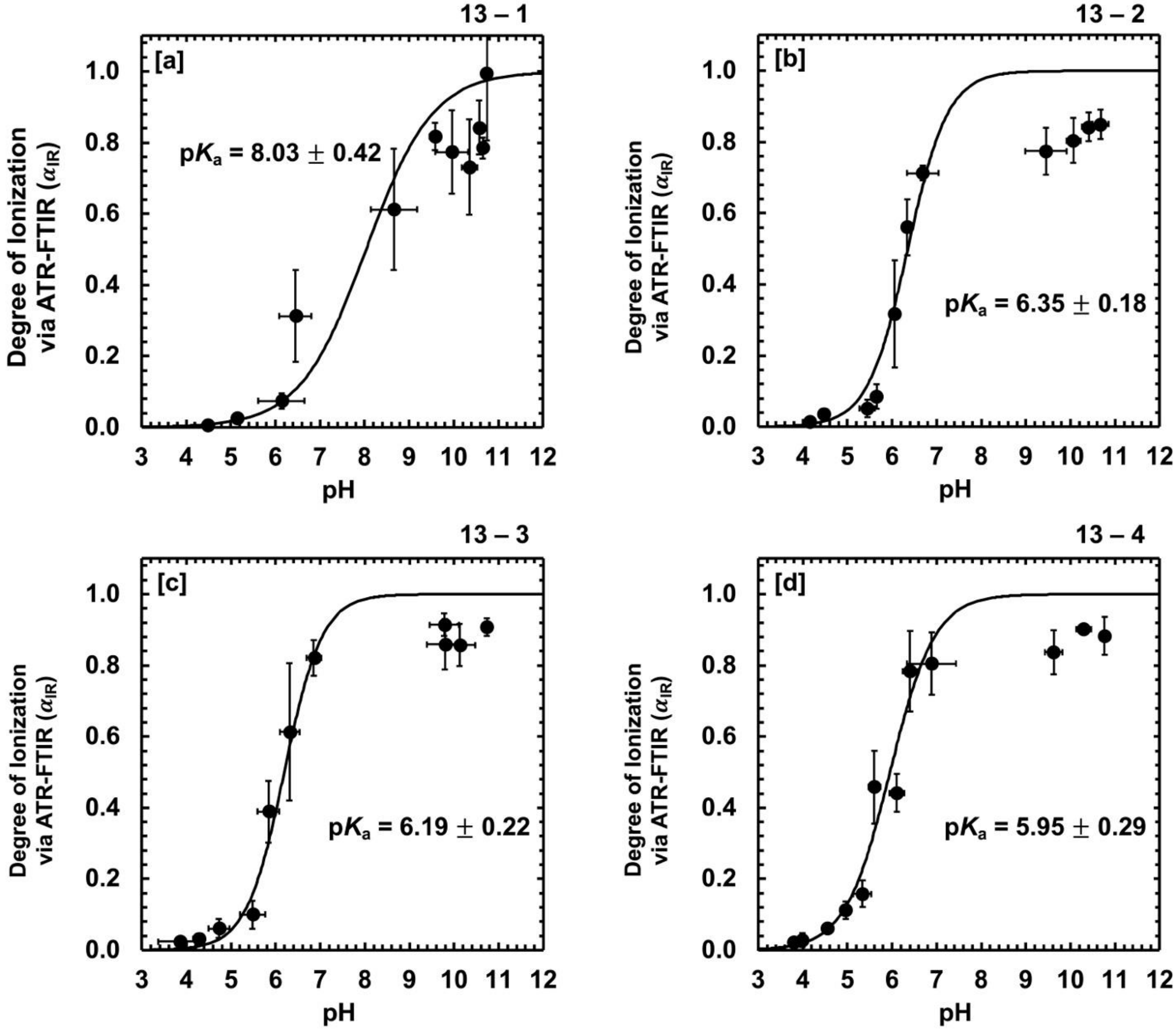

Figure S16. Degree of ionization (α_{IR}) vs. pH of **[a]** 13 – 1, **[b]** 13 – 2, **[c]** 13 – 3, and **[d]** 13 – 4 AA–PEGDA series (mIEC: 1 – 4 mequiv/g, n = 13 series) in 1 M NaCl (aq) solution via ATR–FTIR analysis. Solid lines are the best fitting using the modified Henderson–Hasselbalch equation. Error bars are included.

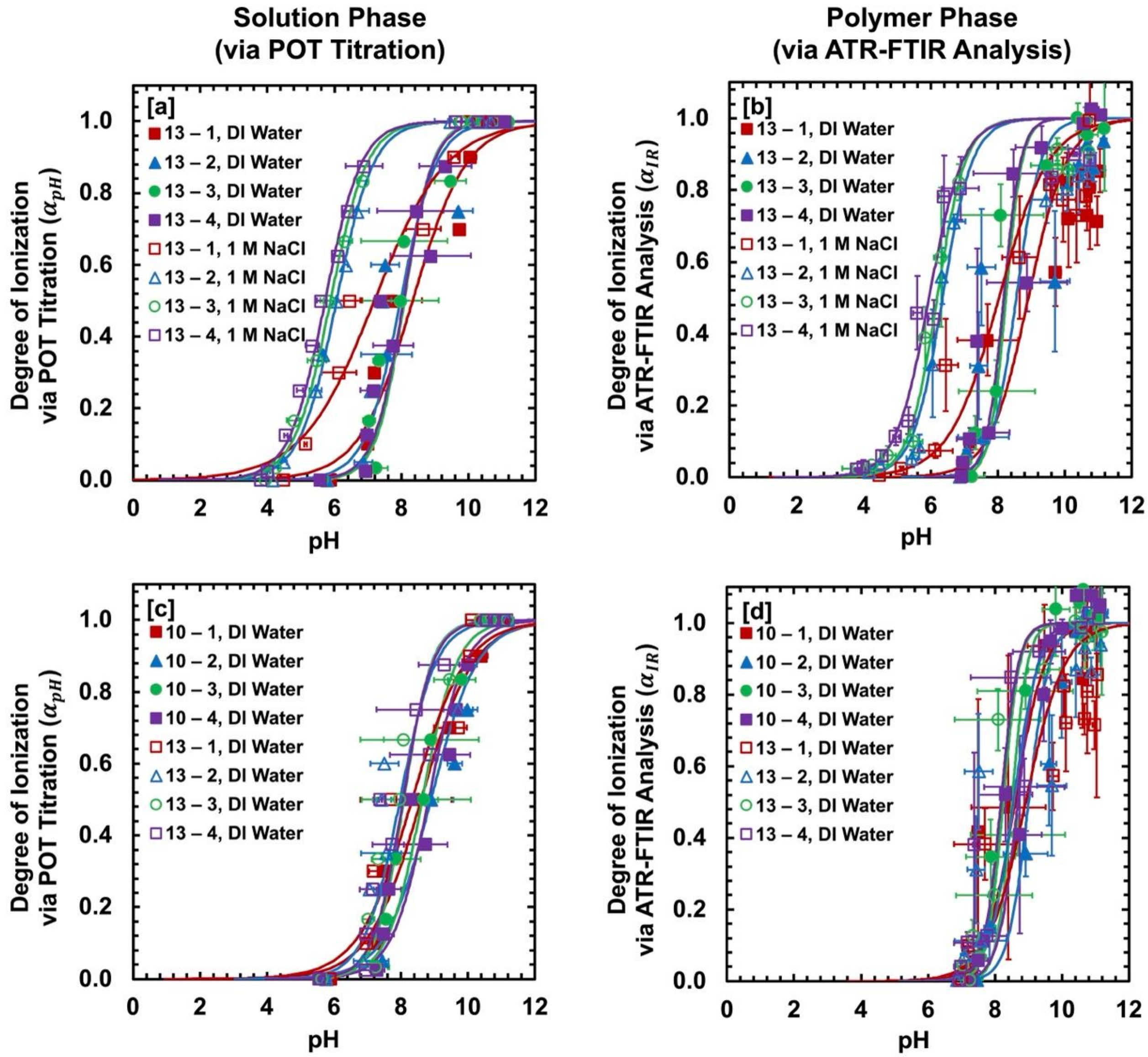

Figure S17. [a, c] Degree of ionization (α_{pH}) vs. pH of AA–PEGDA series (mIEC = 0 – 4 mequiv/g) via POT titration. Experimental data are symbols. Solid a lines are the best fitting using the modified Henderson–Hasselbalch equation. **[a]** n = 13 series in DI water and 1 M NaCl (aq) solution. **[c]** n = 10 and n = 13 series in DI water. **[b, d]** Degree of ionization (α_{IR}) vs. pH of AA–PEGDA series via ATR–FTIR analysis. **[b]** n = 13 series in DI water and 1 M NaCl (aq) solution. **[d]** n = 10 and n = 13 series in DI water. Error bars are included.

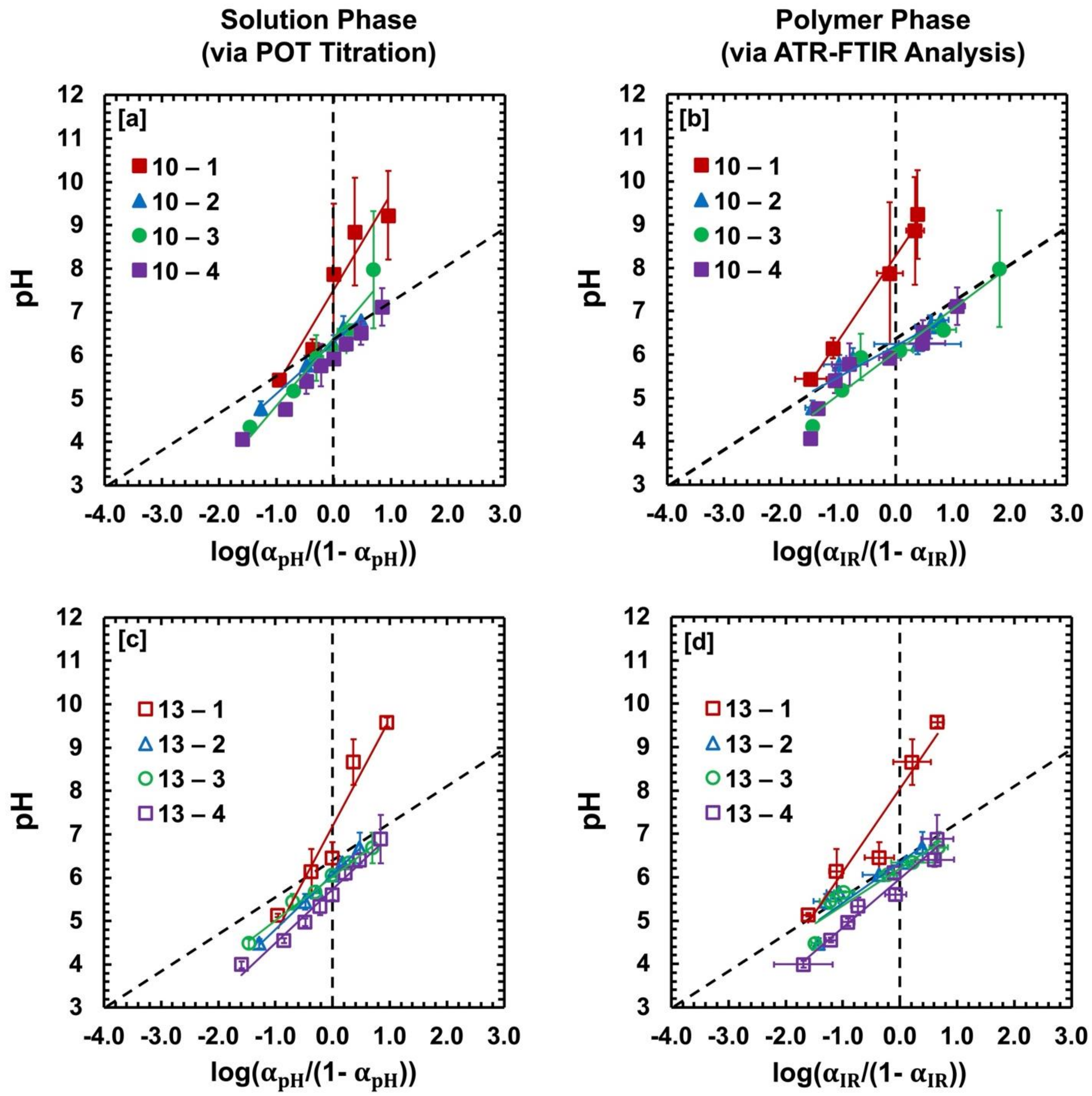

Figure S18. pH vs. $\log(\alpha/(1-\alpha))$ in AA–PEGDA series via POT titration and ATR–FTIR analysis in 1 M NaCl (aq) solution. n = 10 series via **[a]** POT titration and **[b]** ATR–FTIR analysis. n = 13 series via **[c]** POT titration and **[d]** ATR–FTIR analysis. Solid lines are the best fitting using the modified Henderson–Hasselbalch equation. Vertical dashed lines are where α = 0.5 (the halfway point) to determine pK_a. Diagonal dashed lines are where B = 1.

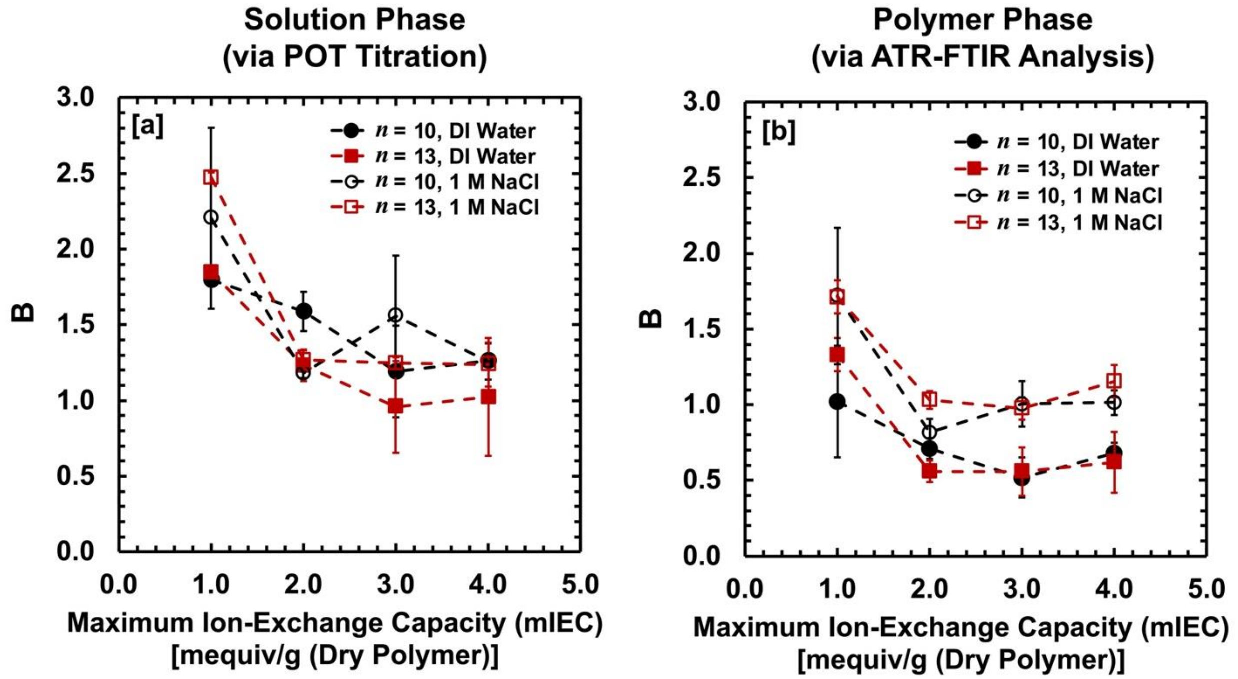

Figure S19. *B* values vs. the maximum ion–exchange capacity (mIEC) of AA–PEGDA series via **[a]** POT titration and **[b]** ATR–FTIR analysis in DI water (filled symbols) and 1 M NaCl (aq) solution (unfilled symbols). Dashed lines are used to guide the eyes. Error bars are included.

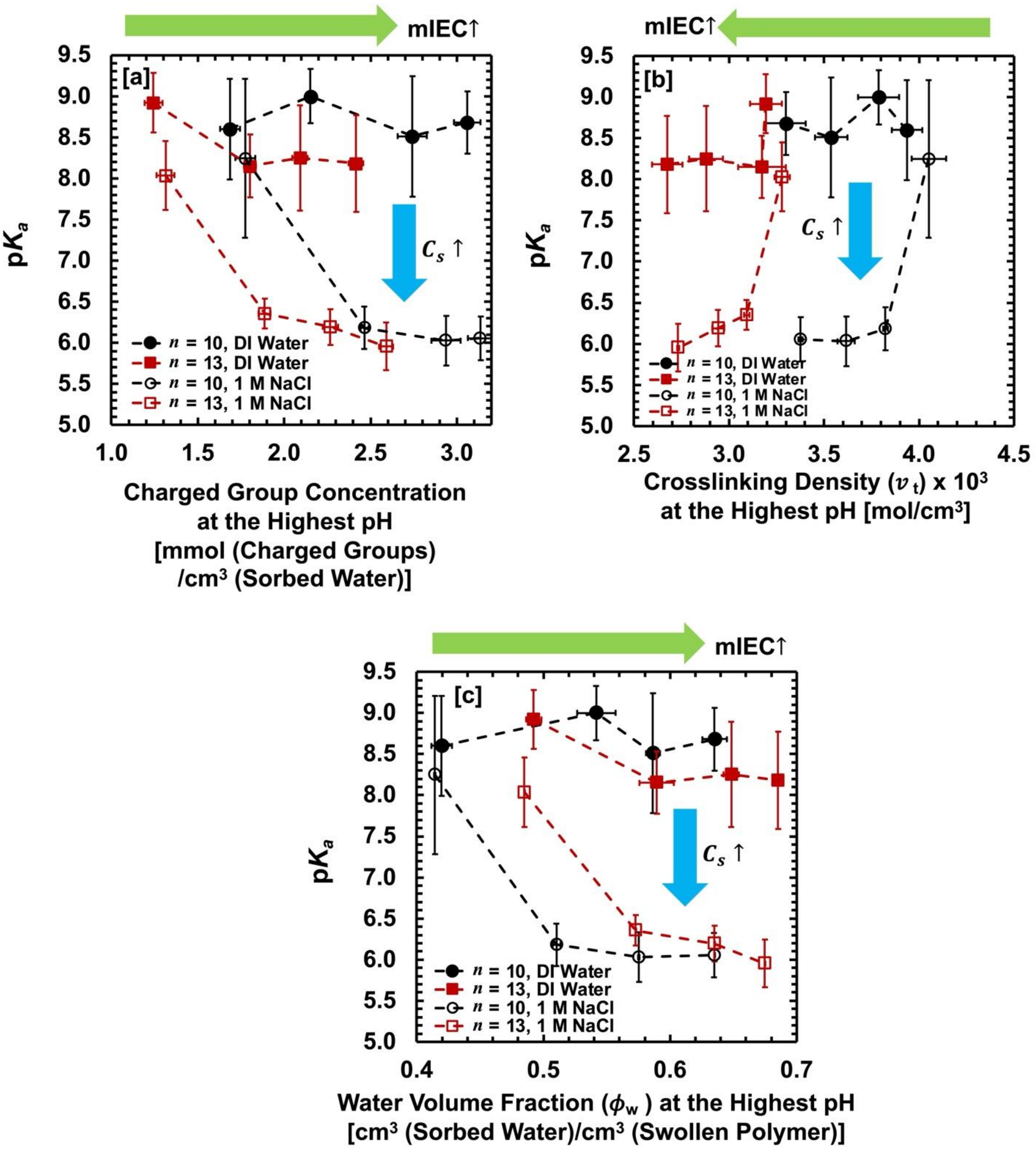

Figure S20. pK_a (via ATR–FTIR analysis) vs. **[a]** charged group concentration (C_C^m), **[b]** cross–linking density (v_t), and **[c]** water volume fraction (ϕ_w) at the highest pH (pH = 11 – 12). Dashed lines are used to guide the eyes. Error bars are included.

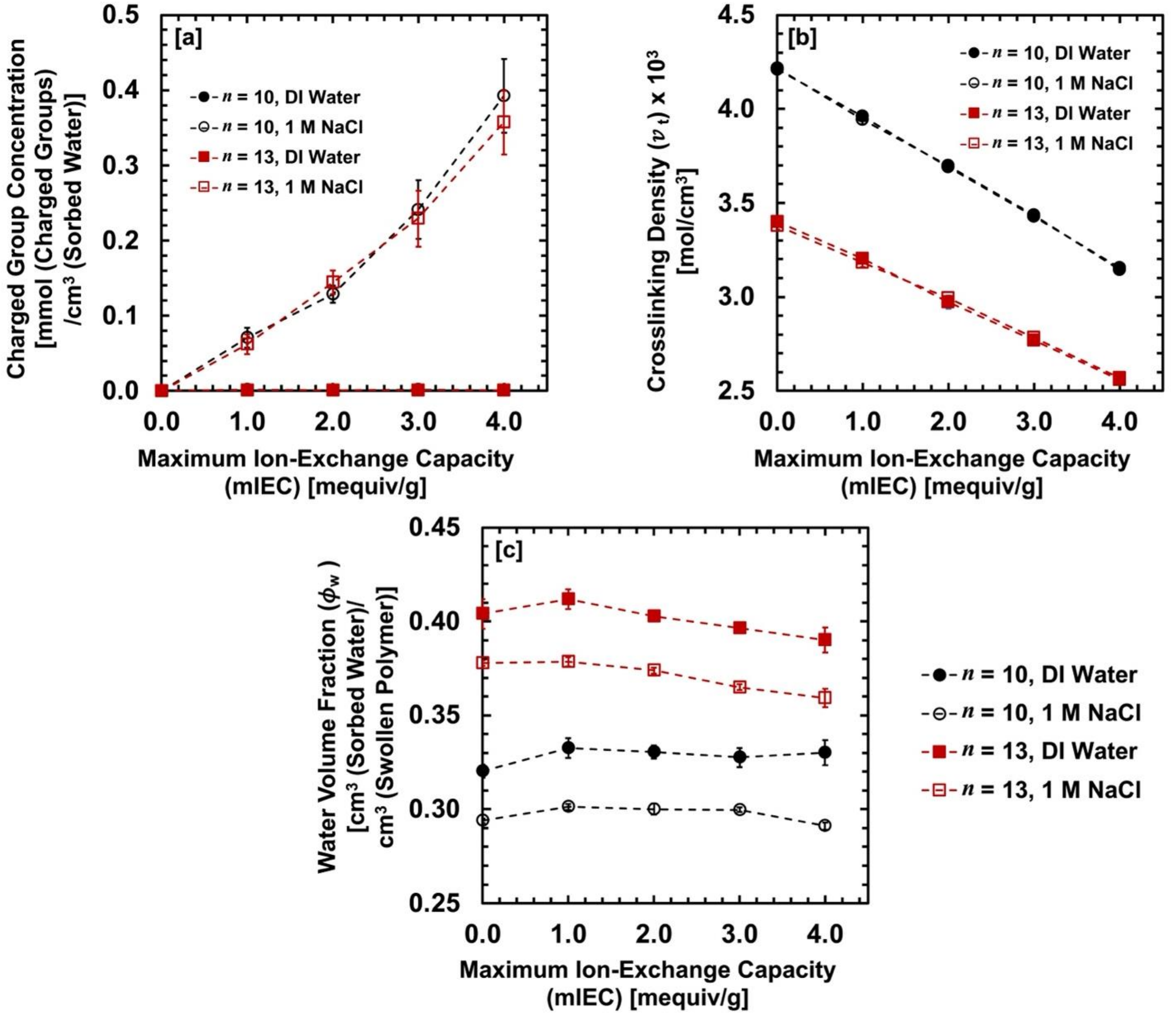

Figure S21. Effect of the maximum ion–exchange capacity (mIEC) on **[a]** charged group concentration (C_C^m), **[b]** cross–linking density (v_t), and **[c]** water volume fraction (ϕ_w) of AA–PEGDA series in DI water and 1 M NaCl (aq) solution at the lowest pH (pH = 3 – 4). Dashed lines are used to guide the eyes. Error bars are included.

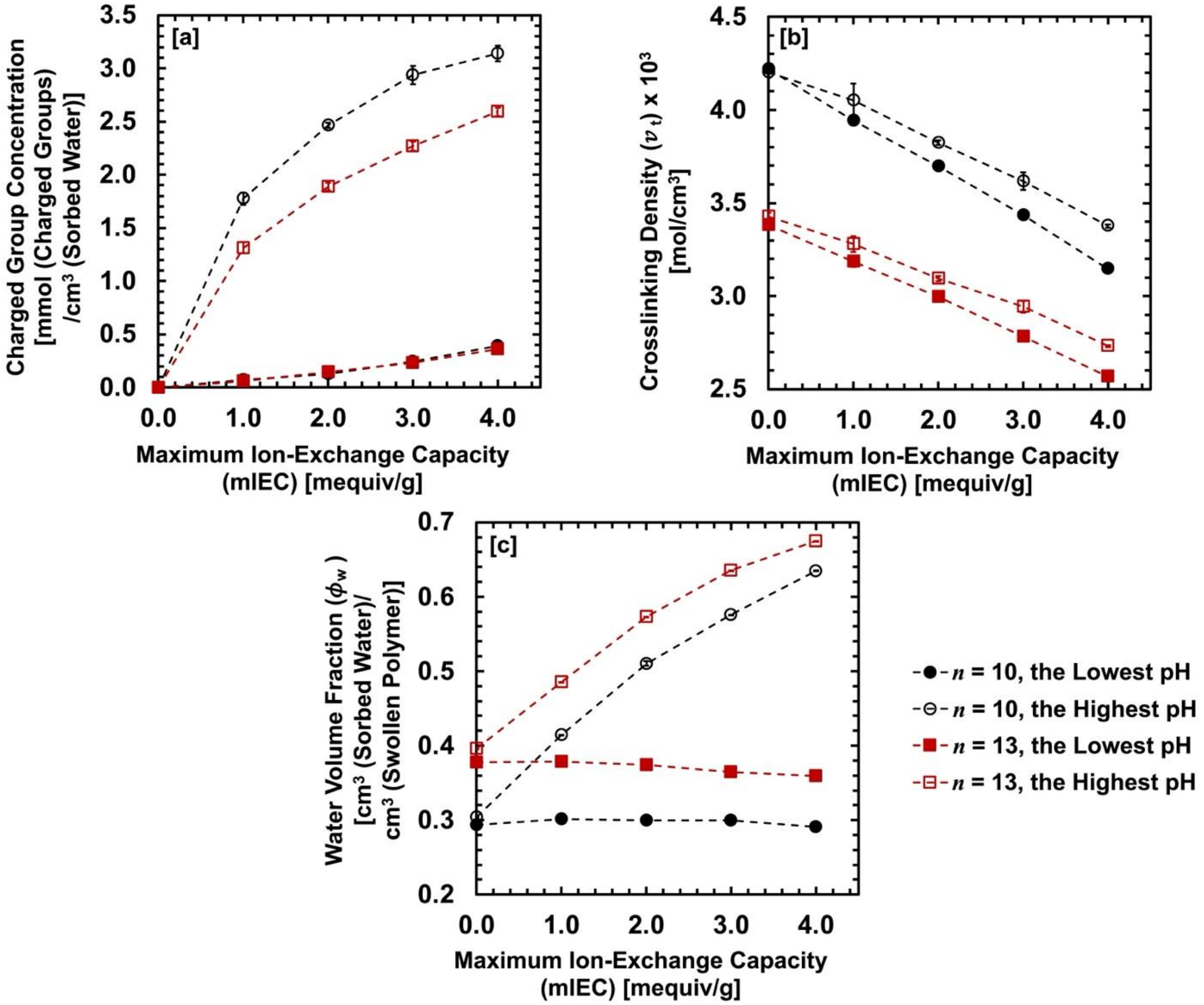

Figure S22. Effect of the maximum ion–exchange capacity (mIEC) on **[a]** charged group concentration (C_C^m), **[b]** cross–linking density (ν_t), and **[c]** water volume fraction (ϕ_w) of AA–PEGDA series in 1 M NaCl (aq) solution at the lowest pH (pH = 3 – 4) and the highest pH (pH = 11 – 12). Dashed lines are used to guide the eyes. Error bars are included.

Table S3. Charged group concentration (C_m^c), cross–linking density (υ_t), water uptake (w_u), and water volume fraction (ϕ_w) of AA–PEGDA series at the lowest pH (pH = 3 – 4) and the highest pH (pH = 11 – 12) in DI water and 1 M NaCl (aq) solution.

n	10					13				
mIEC [mequiv/g]	0	1	2	3	4	0	1	2	3	4
C_m^c at the lowest pH in DI water	–	0.001± 0.001	0.001± 0.001	0.001± 0.001	0.001± 0.000	–	0.001± 0.001	0.001± 0.000	0.001± 0.001	0.001± 0.000
C_m^c at the highest pH in DI water	–	1.686± 0.061	2.151± 0.098	2.742± 0.111	3.060± 0.182	–	1.243± 0.024	1.803± 0.096	2.092± 0.016	2.416± 0.031
C_m^c at the lowest pH in 1 M NaCl	–	0.071± 0.013	0.129± 0.011	0.241± 0.039	0.392± 0.049	–	0.062± 0.013	0.144± 0.016	0.229± 0.037	0.357± 0.042
C_m^c at the highest pH in 1 M NaCl	–	1.775± 0.057	2.465± 0.024	2.937± 0.085	3.138± 0.076	–	1.313± 0.052	1.887± 0.035	2.266± 0.044	2.591± 0.041
υ_t at the lowest pH in DI water	4.210± 0.006	3.959± 0.016	3.689± 0.019	3.429± 0.012	3.153± 0.008	3.400± 0.016	3.204± 0.007	2.972± 0.034	2.767± 0.026	2.557± 0.011
υ_t at the highest pH in DI water	4.193± 0.017	3.936± 0.081	3.788± 0.108	3.537± 0.085	3.300± 0.104	3.331± 0.091	3.194± 0.083	3.172± 0.126	2.880± 0.089	2.674± 0.079
υ_t at the lowest pH in 1 M NaCl	4.218± 0.014	3.942± 0.025	3.697± 0.021	3.434± 0.018	3.145± 0.018	3.380± 0.022	3.183± 0.026	2.994± 0.009	2.782± 0.018	2.569± 0.010
υ_t at the highest pH in 1 M NaCl	4.202± 0.028	4.052± 0.090	3.824± 0.013	3.617± 0.046	3.376± 0.010	3.425± 0.022	3.279± 0.041	3.093± 0.013	2.943± 0.030	2.731± 0.005
w_u at the lowest pH in DI water	0.392± 0.006	0.402± 0.006	0.398± 0.006	0.387± 0.007	0.388± 0.010	0.570± 0.016	0.580± 0.014	0.553± 0.006	0.529± 0.006	0.509± 0.015
w_u at the highest pH in DI water	0.400± 0.039	0.593± 0.022	0.930± 0.042	1.094± 0.044	1.307± 0.078	0.589± 0.017	0.805± 0.016	1.109± 0.059	1.434± 0.011	1.656± 0.021
w_u at the lowest pH in 1 M NaCl	0.343± 0.002	0.353± 0.002	0.345± 0.002	0.340± 0.002	0.323± 0.004	0.513± 0.001	0.507± 0.003	0.487± 0.002	0.463± 0.003	0.444± 0.006
w_u at the highest pH in 1 M NaCl	0.361± 0.012	0.563± 0.018	0.811± 0.008	1.021± 0.029	1.275± 0.031	0.547± 0.013	0.762± 0.030	1.060± 0.019	1.324± 0.026	1.544± 0.024
ϕ_w at the lowest pH in DI water	0.320± 0.004	0.333± 0.005	0.330± 0.004	0.328± 0.005	0.330± 0.007	0.404± 0.008	0.412± 0.005	0.403± 0.001	0.396± 0.002	0.390± 0.007
ϕ_w at the highest pH in DI water	0.325± 0.021	0.420± 0.008	0.542± 0.015	0.586± 0.005	0.635± 0.010	0.407± 0.008	0.492± 0.006	0.590± 0.014	0.648± 0.006	0.685± 0.004
ϕ_w at the lowest pH in 1 M NaCl	0.294± 0.001	0.301± 0.002	0.300± 0.003	0.300± 0.001	0.291± 0.002	0.378± 0.001	0.378± 0.003	0.374± 0.002	0.365± 0.002	0.359± 0.005
ϕ_w at the highest pH in 1 M NaCl	0.304± 0.000	0.414± 0.001	0.510± 0.003	0.575± 0.000	0.635± 0.000	0.396± 0.000	0.485± 0.000	0.573± 0.000	0.635± 0.000	0.675± 0.000

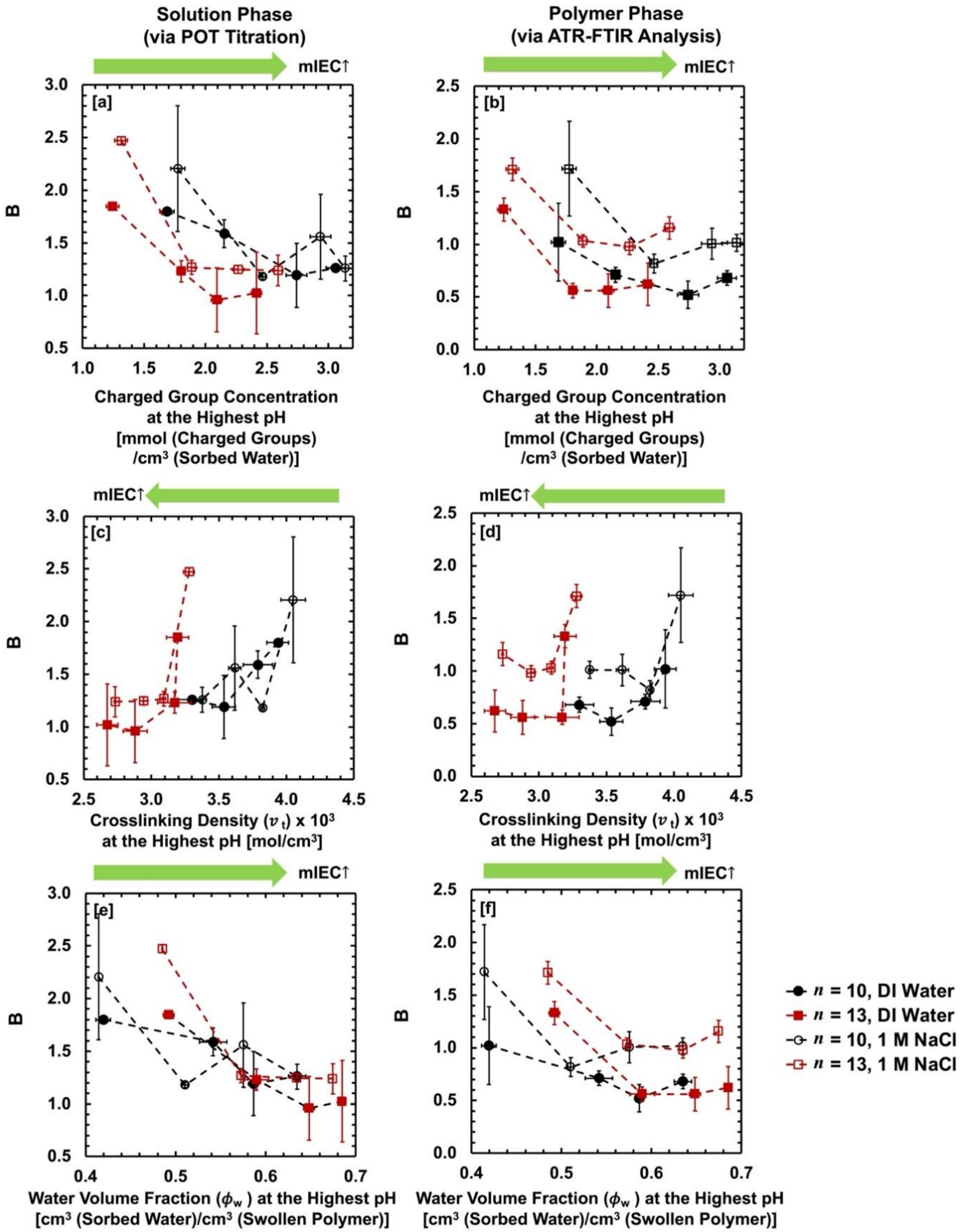

Figure S23. B values vs. **[a, b]** charged group concentration (C_C^m), **[c, d]** cross–linking density (v_t), and **[e, f]** water volume fraction (ϕ_w) at the highest pH (pH = 11 – 12). B values were determined via **[a, c, e]** POT titration and **[b, d, f]** ATR–FTIR analysis. Dashed lines are used to guide the eyes. Error bars are included.

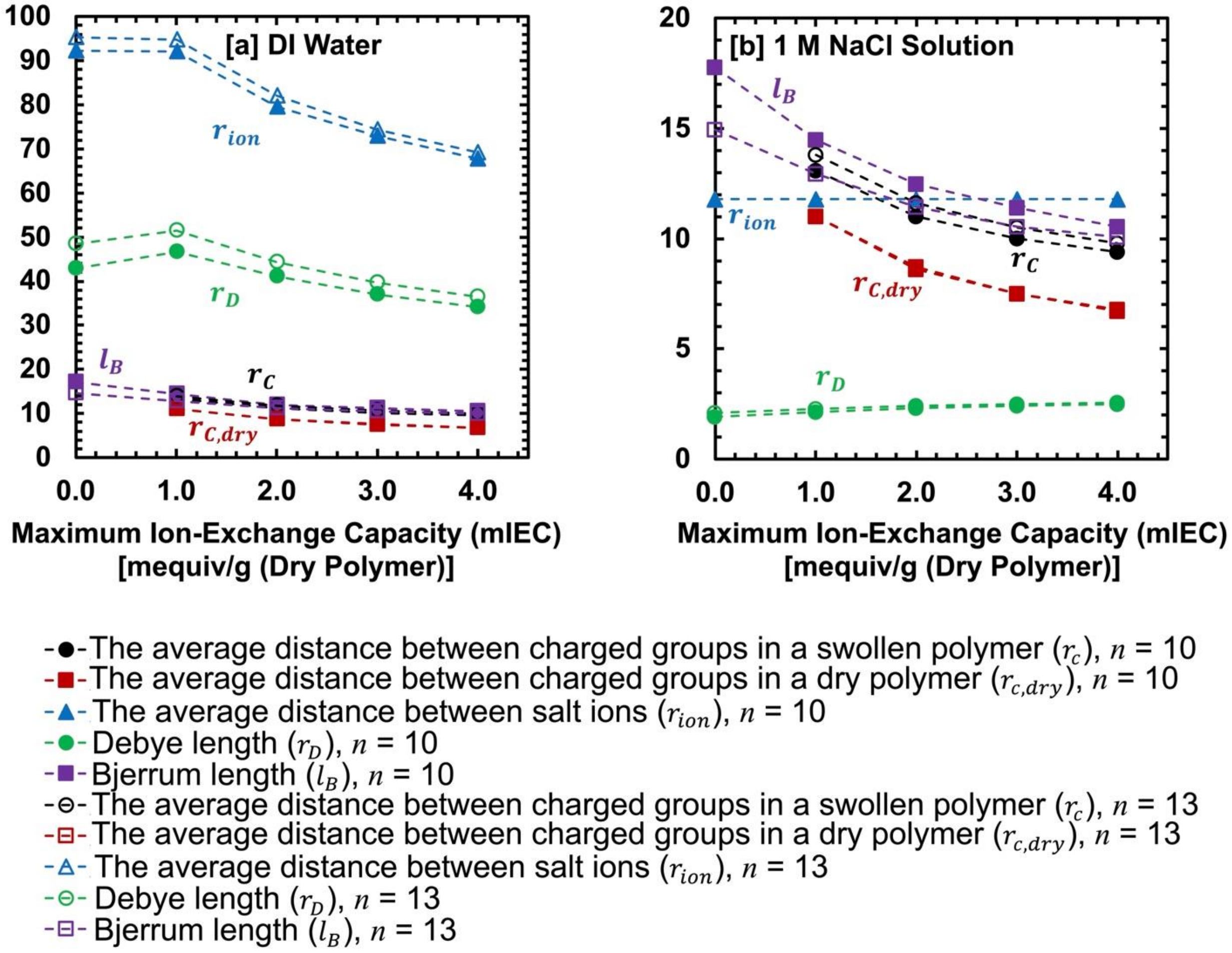

Figure S24. Relevant length scales vs. the maximum ion–exchange capacity (mIEC) of AA–PEGDA series in **[a]** DI water and **[b]** 1 M NaCl (aq) solution at the highest pH (pH = 11 – 12). (1) The average distance between charged groups (r_c) in a swollen polymer and a dry polymer, (2) the average distance between salt ions in an external solution (r_{ion}), and the respective (3) Bjerrum length (l_B) and (4) Debye screening length (r_D) are shown.

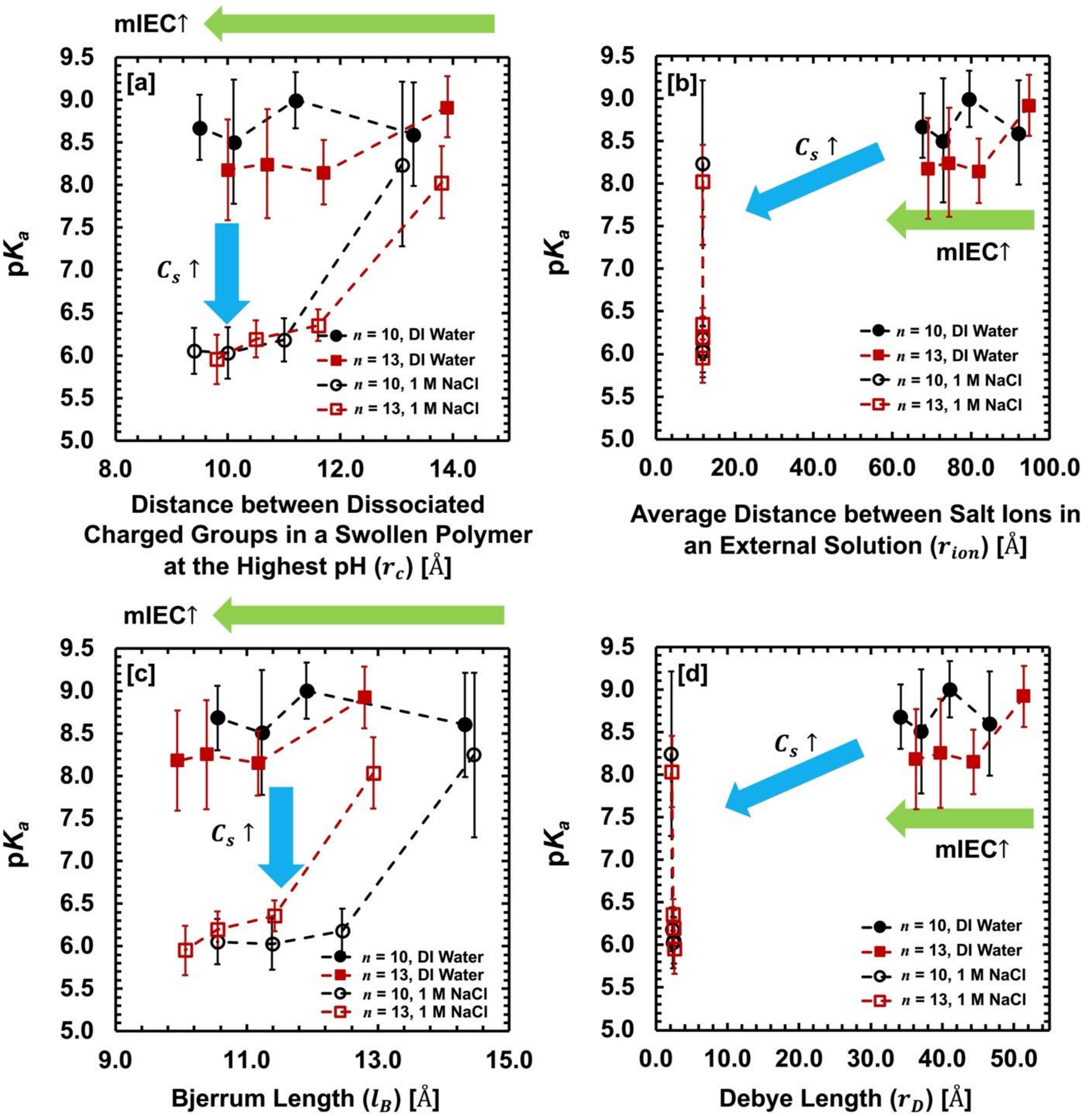

Figure S25. pK_a (via ATR–FTIR analysis) vs. **[a]** the average distance between dissociated charged groups in a swollen polymer (r_c), **[b]** the distance between salt ions in an external solution (r_{ion}), **[c]** Bjerrum length (l_B) and **[d]** Debye screening length (r_D) of AA–PEGDA series in DI water (filled symbols) and 1 M NaCl (aq) solution (unfilled symbols) at the highest pH (pH = 11 – 12). Dashed lines are used to guide the eyes. Error bars are included.

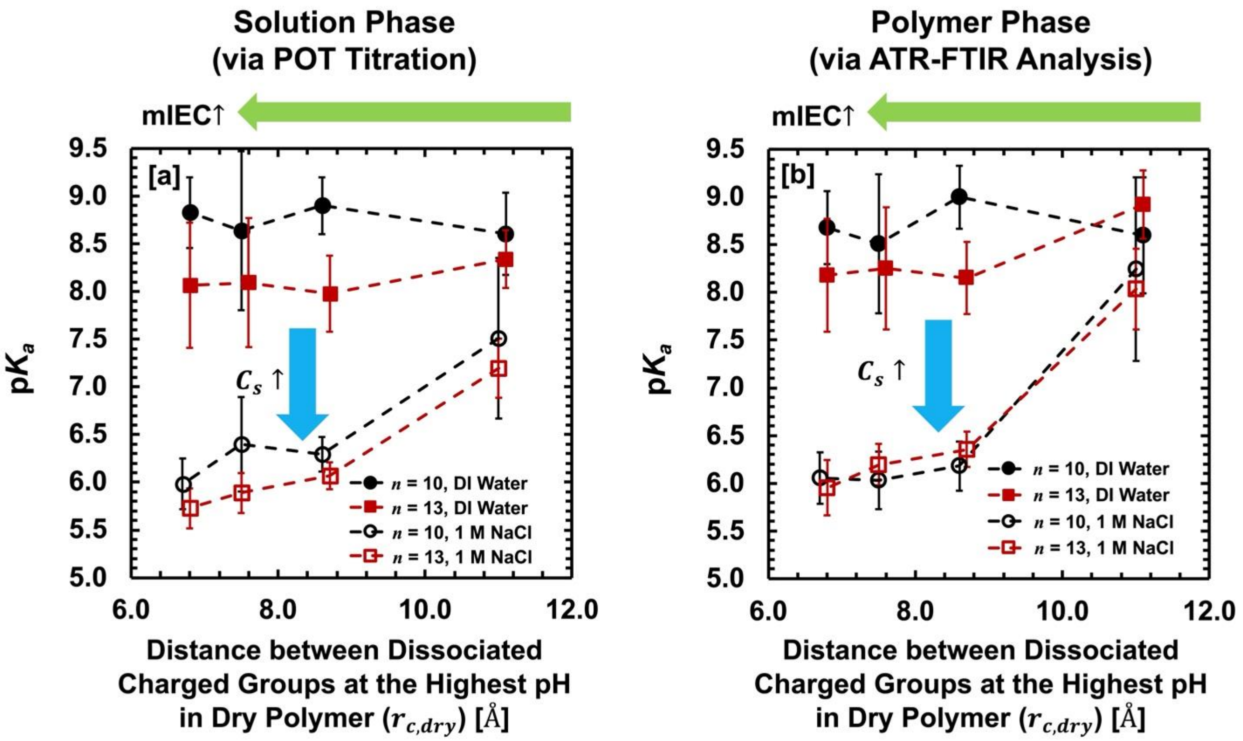

Figure S26. pK_a via **[a]** POT titration and **[b]** ATR-FTIR analysis vs. the average distance between dissociated charged groups at the highest pH (pH = 11 – 12) in dry AA-PEGDA series in DI water and 1 M NaCl (aq) solution.

Table S4. The average distance between dissociated charged groups (r_c) in swollen and dry AA –PEGDA series at the lowest pH (pH = 3 – 5) and the highest pH (pH = 11 – 12) in DI water and 1 M NaCl (aq) solution.

n	10					13				
mIEC [mequiv/g]	0	1	2	3	4	0	1	2	3	4
r_c in a swollen polymer at the highest pH in DI water [Å]	-	13.3 ± 0.6	11.3 ± 0.8	10.1 ± 0.2	9.5 ± 0.2	-	14.0 ± 0.4	11.6 ± 0.2	10.7 ± 0.0	10.0 ± 0.1
r_c in a dry polymer at the highest pH in DI water [Å]	-	11.1 ± 0.1	8.7 ± 0.1	7.5 ± 0.1	6.8 ± 0.1	-	11.1 ± 0.1	8.6 ± 0.1	7.6 ± 0.1	6.8 ± 0.0
r_c in a swollen polymer at the lowest pH in DI water [μm]	-	2.4 ± 1.8	2.7 ± 2.4	1.1 ± 0.6	1.4 ± 0.7	-	1.8 ± 1.2	1.8 ± 1.1	0.9 ± 0.3	1.0 ± 0.4
r_c in a dry polymer at the lowest pH in DI water [µm]	-	2.1 ± 1.6	2.3 ± 2.1	1.0 ± 0.6	1.2 ± 0.6	-	1.5 ± 1.0	1.5 ± 0.9	0.8 ± 0.3	0.8 ± 0.3
r_c in a swollen polymer at the highest pH in 1 M NaCl [Å]	-	13.1 ± 0.1	11.0 ± 0.0	9.9 ± 0.1	9.4 ± 0.1	-	13.8 ± 0.2	11.5 ± 0.1	10.5 ± 0.1	9.8 ± 0.1
r_c in a dry polymer at the highest pH in 1 M NaCl [Å]	-	11.0 ± 0.0	8.7 ± 0.0	7.5 ± 0.0	6.8 ± 0.1	-	11.0 ± 0.1	8.7 ± 0.0	7.5 ± 0.0	6.8 ± 0.0
r_c in a swollen polymer at the lowest pH in 1 M NaCl [nm]	-	50 ± 9	40 ± 4	31 ± 5	28 ± 5	-	42 ± 9	30 ± 3	25 ± 4	20 ± 2
r_c in a dry polymer at the lowest pH in 1 M NaCl [nm]	-	44 ± 8	40 ± 3	28 ± 5	25 ± 4	-	35 ± 8	25 ± 3	22 ± 4	17 ± 2

Table S5. The average distance between salt ions in an external solution (r_{ion}), Bjerrum length (l_B) and Debye screening length (r_D) of AA–PEGDA series in DI water and 1 M NaCl (aq) solution.

n	10					13				
mIEC [mequiv/g]	0	1	2	3	4	0	1	2	3	4
r_{ion} in DI water [Å]	92.3	92.1	79.6	72.9	67.7	95.3	94.7	82.0	74.4	69.1
r_{ion} in 1 M NaCl [Å]	11.8	11.8	11.8	11.8	11.8	11.8	11.8	11.8	11.8	11.8
l_B in DI water [Å]	17.0	14.3	11.9	11.2	10.6	14.6	12.8	11.2	10.4	9.9
l_B in 1 M NaCl [Å]	17.8	14.5	12.5	11.4	10.6	14.9	12.9	11.4	10.6	10.1
r_D in DI water [Å]	42.9	46.6	41.0	37.0	34.2	48.5	51.4	44.3	39.7	36.3
r_D in 1 M NaCl [Å]	1.9	2.1	2.3	2.4	2.5	2.1	2.3	2.4	2.5	2.6

S.3. References

(1) Kim, Y.; Kim, T.; Kang, D. E.; Kracaw, R. B.; Lukaszewski, A. J.; Szymanski, J. S.; Rahman, C. M.; Shaqfeh, M. A.; Tierney, K. M.; Doan, H. Weak polyelectrolyte membranes with a wide ion-exchange capacity (IEC) range and limited water swelling in clean technologies for sustainability. *ACS Applied Polymer Materials* **2024**, *6* (18), 11334-11349. DOI: 10.1021/acsapm.4c01877.

(2) Kim, Y.; Kim, T.; Kang, D. E.; Szymanski, J. S.; Kracaw, R. B.; Lukaszewski, A. J.; Tierney, K. M.; Shaqfeh, M. A.; Rahman, C. M.; Jeung Oh, H. Determination of Carboxyl Dissociation Degree and p K a in Weak Polyelectrolyte Membranes via POT Titration and FTIR Analysis for Clean Technologies in Sustainability. *Macromolecules* **2024**, *57* (22), 10844-10860. DOI: 10.1021/acs.macromol.4c02139.

(3) Müller, M.; Wirth, L.; Urban, B. Determination of the carboxyl dissociation degree and pKa value of mono and polyacid solutions by FTIR titration. *Macromolecular Chemistry and Physics* **2021**, *222* (4), 2000334. DOI: 10.1002/macp.202000334.

(4) Silverstein, R. M.; Bassler, G. C. Spectrometric identification of organic compounds. *Journal of Chemical Education* **1962**, *39* (11), 546. DOI: 10.1021/ed039p546.

(5) Zimudzi, T. J.; Feldman, K. E.; Sturnfield, J. F.; Roy, A.; Hickner, M. A.; Stafford, C. M. Quantifying carboxylic acid concentration in model polyamide desalination membranes via Fourier transform infrared spectroscopy. *Macromolecules* **2018**, *51* (17), 6623-6629. DOI: 10.1021/acs.macromol.8b01194.

(6) Witte, R. P.; Blake, A. J.; Palmer, C.; Kao, W. J. Analysis of poly (ethylene glycol)-diacrylate macromer polymerization within a multicomponent semi-interpenetrating polymer network system. *Journal of Biomedical Materials Research Part A: An Official Journal of The Society for Biomaterials, The Japanese Society for Biomaterials, and The Australian Society for Biomaterials and the Korean Society for Biomaterials* **2004**, *71* (3), 508-518. DOI: 10.1002/jbm.a.30179.

(7) Hamid, Z. A.; Lim, K. Evaluation of UV-crosslinked poly (ethylene glycol) diacrylate/poly (dimethylsiloxane) dimethacrylate hydrogel: properties for tissue engineering application. *Procedia Chemistry* **2016**, *19*, 410-418. DOI: 10.1016/j.proche.2016.03.032.
